# Incidence of hyperkalemia RAASi and SGLT-2i treatment in individuals with diabetic kidney disease: a systematic review and network meta-analysis

**DOI:** 10.3389/fphar.2024.1462965

**Published:** 2025-01-27

**Authors:** Yahui Yuan, Chun Chen, Yuping Lin, Yehao Luo, Zhaojun Yang, Jingyi Guo, Qiaoyun Liu, Lu Sun, Guanjie Fan

**Affiliations:** ^1^ The Second Clinical Medical School, Guangzhou University of Chinese Medicine, Guangzhou, China; ^2^ Department of Endocrinology, The Second Affiliated Hospital of Guangzhou University of Chinese Medicine, Guangzhou, China; ^3^ Department of Endocrinology, Guangdong Provincial Hospital of Chinese Medicine, Guangzhou, China; ^4^ Wangjing Hospital, China Academy of Chinese Medical Sciences, Beijing, China

**Keywords:** diabetic kidney disease, renin-angiotensin-aldosterone system inhibitors, hyperkalemia, SGLT-2i, network meta-analysis

## Abstract

**Background:**

This study aims to evaluate the incidence of hyperkalemia and serum potassium levels associated with the use of sodium-glucose cotransporter-2 inhibitors (SGLT-2i), renin‐angiotensin‐aldosterone system inhibitors (RAASi) and concurrent use of these medications in individuals with diabetic kidney disease (DKD).

**Methods:**

A comprehensive systematic search was performed in EMBASE, the Cochrane Central Register of Controlled Trials (CENTRAL), Web of Science, Scopus, and PubMed database, covering studies up to March 2024. Relevant randomized controlled trials (RCT) included adults with DKD who were treated with SGLT-2i and RAASi or their combination, with a minimum follow-up duration of 12 weeks. The primary outcomes assessed were the incidence of hyperkalemia and serum potassium levels were the primary outcomes assessed. The surface under the cumulative ranking curves (SUCRA) was utilized for ranking purposes.

**Results:**

The study included 36 trials, encompassing 45,120 participants, comparing various interventions. SGLT-2i (SUCRA = 88.5%) was found to significantly reduce the risk of hyperkalemia. In contrast, the combination of ACEI/ARB + MRA (SUCRA = 5.7%) increased the risk of hyperkalemia. However, when SGLT-2i was added to the ACEI/ARB + MRA regimen, the incidence of hyperkalemia was found to decrease. Subgroup analyses on MRA showed that ACEI/ARB + spironolactone posed the highest risk of hyperkalemia. ACEI/ARB + SGLT-2i mitigated serum potassium level.

**Conclusion:**

SGLT-2i was effective in reducing the incidence of hyperkalemia incidence, whereas a combination of ACEI/ARB and MRA might elevate the incidence of hyperkalemia in individuals with DKD.

**Systematic Review Registration:**

https://www.crd.york.ac.uk/PROSPERO/#recordDetails, identifier CRD42024552810.

## 1 Introduction

Diabetic kidney disease (DKD), a major microvascular complications of diabetes mellitus (DM), is characterized by proteinuria and progressive impairment of the renal function caused by diabetes, ultimately leading to end-stage renal disease (ESRD) (McClellan et al., 2011). Approximately 40% of individuals with DM will develop DKD, making it the leading contributor of chronic kidney disease (CKD) globally (Tuttle et al., 2022). As the global prevalence of diabetes is expected to rise by nearly 50% in the subsequent 24 years, from 537 million to 783 million individuals, leading to a corresponding increase in the incidence of DKD (Alicic et al., 2017; Magliano et al., 2021). Effective management of DKD hinges on controlling blood glucose and blood pressure, blocking the renin-angiotensin-aldosterone system (RAAS) with a single agent are cornerstones of treating DKD (de Boer et al., 2020; Lytvyn et al., 2020).

ACEI and ARB are commonly prescribed for DKD patients, due to their proven ability to delay the progression of kidney dysfunction and decrease proteinuria. However, the use of dual RAAS inhibition therapy has been associated with a higher risk of hyperkalemia (Strippoli et al., 2006; Whitlock et al., 2023; Costa et al., 2023). Clinical trials focusing on DKD have indicated that steroid mineralocorticoid receptor antagonists (MRAs) are effective in reducing albuminuria in comparison with placebo, though hyperkalemia remains an important side effect (Barrera-Chimal et al., 2022). Sodium-glucose cotransporter-2 inhibitors (SGLT-2i), a novel class of oral antihyperglycemic medications have been developed to block the majority of glucose reabsorption in the renal proximal tubule, thereby reducing both renal and cardiovascular risks (Cai et al., 2020). Recent research has highlighted the safe use of SGLT-2i in individuals with type 2 diabetes mellitus (T2DM) who are at high cardiovascular risk and have CKD, without an increased risk of hyperkalemia (Albakr et al., 2023).

Hyperkalemia, characterized as an increase serum potassium concentration exceeding 5.0 or 5.5 mmol/L, is a potentially life-threatening electrolyte abnormality. Patients with CKD, DM, heart failure (HF) and those receiving RAASi are particularly prone to developing hyperkalemia (Valdivielso et al., 2021). Individuals with DKD are particularly susceptible to hypoinsulinemia and hyperglycemia, which can further elevate the risk of hyperkalemia (Picard et al., 2020; Montford and Linas, 2017). The effects of hyperkalemia on cardiac function include depolarization, leading to shorter action potentials and an increased risk of cardiovascular morbidity. Additionally, hyperkalemia can manifest as neuromuscular symptoms, metabolic acidosis, and suppression of ammoniagenesis, ultimately raising the likelihood of mortality, progression of CKD, and hospitalization (Palmer et al., 2021). This research aims to evaluate the effect of SGLT-2i, RAASi, and their combination on the incidence of hyperkalemia and serum potassium levels in patients with DKD.

## 2 Methods

This study has been registered in PROSPERO International Prospective Register of Systematic Reviews (Register number: CRD42024552810). The network meta-analysis (NMA) was meticulously designed, conducted and presented in accordance with quality standards for reporting systematic reviews (Hutton et al., 2015).

### 2.1 Literature search

EMBASE, the Cochrane Central Register of Controlled Trials (CENTRAL), Web of science, Scopus and PubMed were systematically searched for relevant English-language literature without time restrictions from inception until March 2024. The search term was predefined and detailed in Supplementary Table S1. Moreover, the articles that are included in the reference list can also be searched manually. Data extraction was independently performed by two authors, with any discrepancies resolved through discussion or by consulting a third author to reach a consensus. The extracted information included details such as: the first author, publication time, location of the study conducted, study duration, age, total number of participants included in the study, clinical trial number, diabetes type, and more.

### 2.2 Eligibility criteria

Eligible trials enrolled adults (≥18 years old) with DKD who were treated with SGLT-2i, angiotensin-converting enzyme inhibitors (ACEI), angiotensin receptor blockers (ARB), renin inhibitor (RI), mineralocorticoid receptor antagonist (MRA) or placebo either as monotherapy or in combination, with a minimum follow-up period of 12 weeks. Our inclusion criteria encompassed randomized controlled trials, *post hoc* analysis, or pooled studies, while conference abstracts and non-English literature were excluded. Additionally, trials with a crossover design were excluded. Each trial had to report at least one outcome related to hyperkalemia or serum potassium levels. Detailed eligibility criteria for inclusion and exclusion in this review are provided in Supplementary Table S2.

### 2.3 Risk of bias assessment

Assessment of the enrolled trials’ methodological quality was conducted with the Cochrane Collaboration Risk of Bias Assessment Tool. Biases such as random-sequence generation, allocation sequence concealment, blinding of participants and clinicians, blinding of outcome assessment, completeness of outcome data, and selective outcome reporting were considered. Studies were labeled as high risk of bias if at least 4 out of 7 items were deemed low risk and no single item was considered high risk. If one item was assessed as high risk, the study was categorized as high risk, while the remaining studies were rated as unclear risk (Higgins et al., 2011; Schwingshackl et al., 2018).

### 2.4 Data synthesis and analysis

#### 2.4.1 Available data description

The characteristics of the studies and populations in all included trials were thoroughly described, including important variables such as mean age, sex ratio, and dropout rates. A network diagram was utilized to illustrate the direct comparisons between different interventions for each outcome. The sample sizes for each intervention were represented by the size of the nodes, and the number of included studies was reflected in the width of the lines.

#### 2.4.2 Transitivity assessment

One of the fundamental assumptions in NMA and indirect comparisons is transitivity. The violation of transitivity poses a risk to the validity of key findings in NMA. To assess the transitivity hypothesis, researchers analyzed the distribution of potential effect modifiers in the existing direct comparisons, including baseline serum potassium, baseline serum creatinine, and age.

#### 2.4.3 Inconsistency assessment

The study utilized both local and global approaches to assess consistency in the results. A loop-specific approach and a side-splitting approach were employed to identify significant local inconsistencies (Dias et al., 2010). Additionally, a design-by-treatment interaction model was used to detect inconsistencies stemming from various potential origins across the network at a global level.

### 2.5 Statistical analysis

An NMA with random effects was conducted to establish the overall effect of one treatment compared to another. The NMA allowed for comparisons of different interventions and established a clinically meaningful ranking. For each outcome (hyperkalemia incidence, serum potassium levels), a league table was utilized to present odds ratios (OR) or mean differences (MDs) along with their corresponding 95% confidence intervals (95% CIs). The surface under the cumulative ranking curve (SUCRA) was used to assess the relative ranking of interventions. The calculations were performed using a frequentist framework in STATA17, and network diagrams were created using the R package “netmeta.”

### 2.6 Subgroup analyses

Subgroup analyses were performed by considering baseline eGFR (<60 mL/min/1.73 m^2^ vs. ≥60 mL/min/1.73 m^2^), sample size (<100 vs. ≥100), and therapy duration (<48 weeks vs. ≥48 weeks).

### 2.7 Publication bias

Adjusted funnel plots for comparison were applied to examine the potential existence of small-study effects when 10 or more studies were available (Egger et al., 1997).

### 2.8 Evidence credibility

The Grading of Recommendations Assessment, Development and Evaluation (GRADE) system was used to assess the credibility of evidence. The CINeMA web tool, available at http://cinema.ispm.ch/, was applied to evaluate the results of the NMA. Within-study risk of bias, reporting bias, indirectness, imprecision, heterogeneity and inconsistency were judged qualitatively (Nikolakopoulou et al., 2020). The level of concerns for each treatment effect of NMA were judged as “no concerns,” “some concerns,” or “major concerns” for each of the six domains (Papakonstantinou et al., 2020).

## 3 Results

### 3.1 Characteristics

**FIGURE 1:**
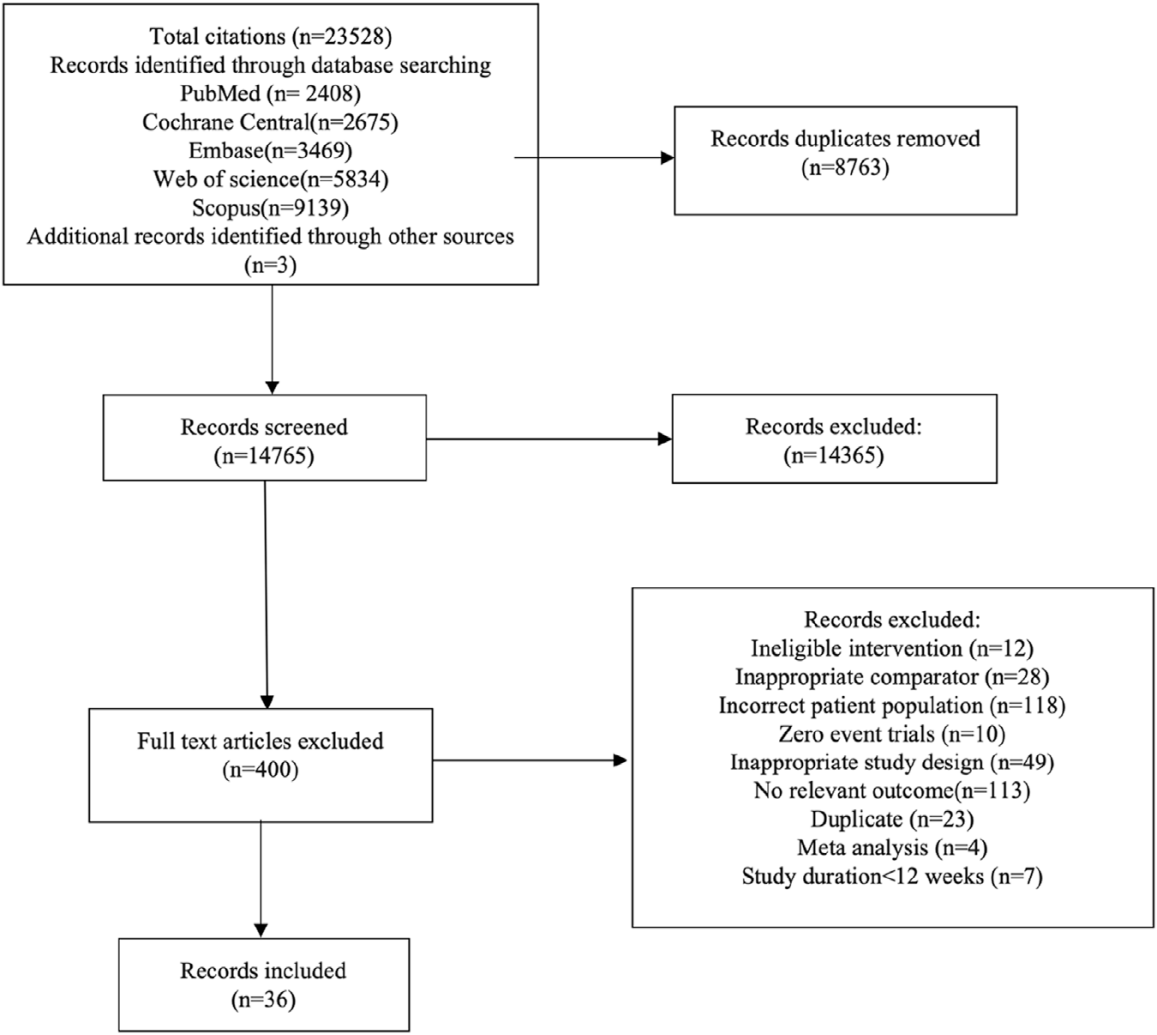
Flow-chart of study selection.

Our literature search identified 23,528 records, from which 400 full-text articles undergoing detailed evaluation (Figure 1). Out of these, 364 were excluded, leading to 36 trials meeting the inclusion criteria and providing sufficient information for the current study. The combined population of DKD patients across all relevant articles was 45,120. Among these trials, 26 were multicenter and predominantly conducted in South America, North America, Central America, Europe, Asia, and other regions. The duration of intervention varied from 12 to 216 weeks, with the average age of enrolled patients falling between 34 and 68.7 years old. Supplementary Table S3 were utilized to summarize the characteristics of the studies meeting the inclusion criteria.

### 3.2 Risk of bias

The risk of bias for each trial is presented in Supplementary Table S4 and Supplementary Figures S1, S2. Main limitations included insufficient reporting on the implementation of blindness among participants, investigators, and outcome assessors in some studies. Among the 36 trials reviewed, 18 trials (50%) were at low risk of randomisation bias, 12 trials (33%) were at low risk of allocation concealment, 24 trials (67%) were at low risk of participant and staff concealment, 17 trials (47%) were at low risk of outcome concealment, 24 trials (67%) were at low risk of incomplete outcome data, 30 trials (83%) were at low risk of selective reporting and 21 studies (58%) were at low risk of other bias. A primary limitation was the insufficient reporting on blinding practices involving participants, investigators, and outcome assessors in some studies.

### 3.3 Network diagrams

**FIGURE 2:**
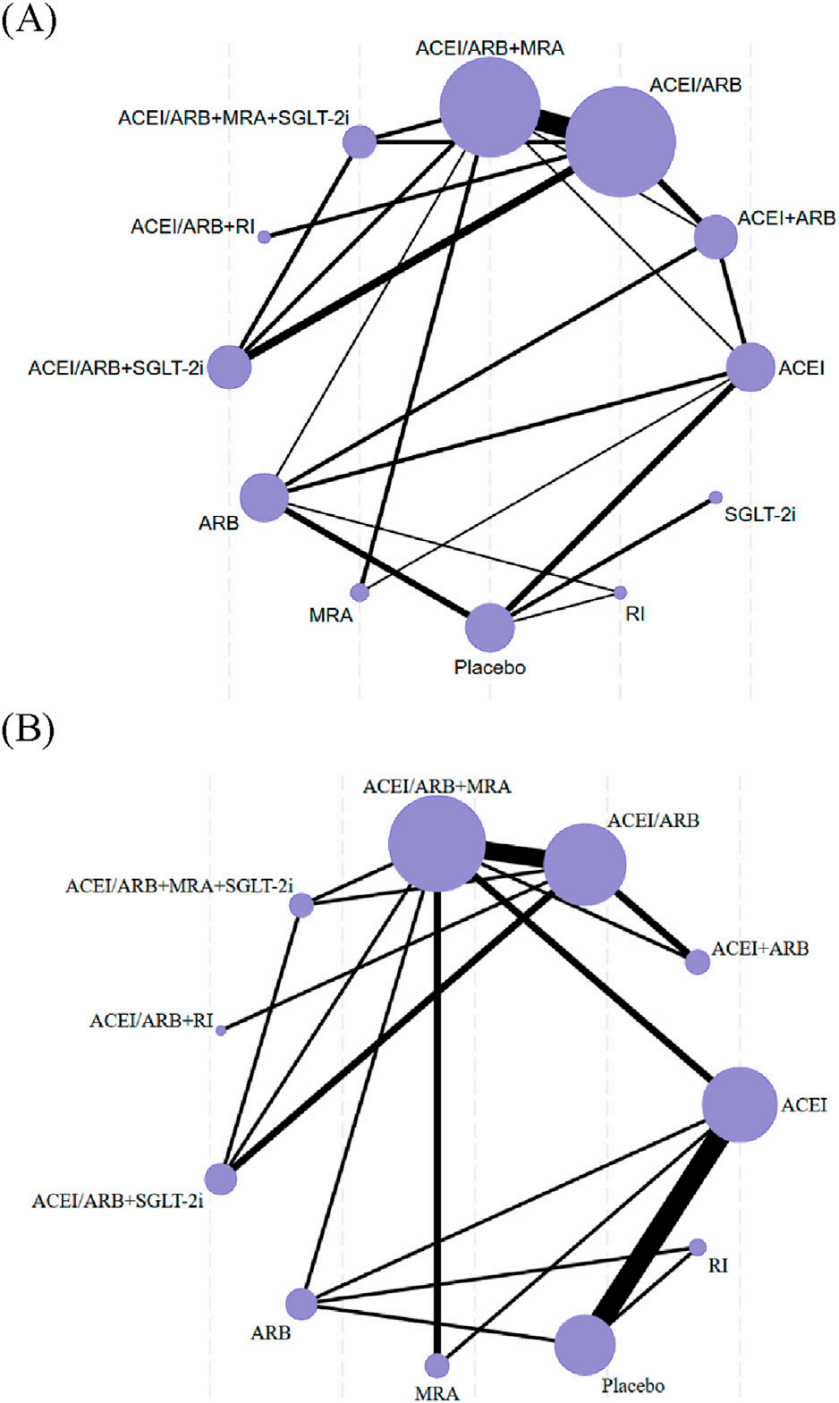
Network diagrams for hyperkalemia **(A)** and serum potassium **(B)**. The size of the nodes is proportional to the total number of participants, and the thickness of the lines is proportional to the number of studies evaluating each direct comparison.

Figure 2 depicted the networks of trials used in the meta-analyses for the risk of hyperkalemia and the level of serum potassium with included studies proportional to the width of lines, and the sample number of each intervention approach proportional to the size of the nodes.

### 3.4 Transitivity

No evidence of transitivity was observed (Supplementary Figures S3–S5). Thus, we concluded that there was no relevant clinical and methodological heterogeneity across treatment comparisons (intransitivity) in the current review.

### 3.5 Inconsistency

The forest plot, presented in Supplementary Figures S6, S7, displayed the pooled effect size for interventions across included studies, along with the findings from the test of inconsistency. The overall test of inconsistency for the risk of hyperkalemia and the level of serum potassium did not show statistical significance (*P* = 0.065 and 0.943, respectively). Evidence of inconsistency was found in the pairwise comparison approach between ACEI + ARB and ACEI/ARB, as well as between ACEI/ARB + MRA along with ACEI/ARB in relation to the risk of hyperkalemia (Supplementary Table S5. The loop-specific method presented some significant inconsistency in the loop formed by ACEI + ARB-ACEI/ARB-ACEI/ARB + MRA for the risk of hyperkalemia (Supplementary Figure S8). No significant differences were observed in serum potassium level between direct and indirect results (Supplementary Table S6; Supplementary Figure S9). Furthermore, a notable inconsistency was found in the design-by-treatment model for the risk of hyperkalemia (*P* = 0.029), but not for serum potassium level (*P* = 0.93).

### 3.6 Contribution matrix

The contribution matrix showed to which extent the direct and indirect comparisons of the various interventions were contributory to the overall effect. The primary contributions to the effects of this study derived from indirect comparisons (Supplementary Table S7).

### 3.7 Table league

Table 1 showed the results of the NMA for the risk of hyperkalemia and the level of serum potassium. The analysis revealed that ACEI/ARB (OR: 1.95; 95% CI: 1.19–3.18), ACEI/ARB + SGLT-2i (OR: 2.69; 95% CI: 1.44–5.02), placebo (OR: 2.54; 95% CI: 1.04–6.18), and SGLT-2i (OR: 4.12; 95% CI: 1.43–11.87) were more effective than ACEI + ARB in reducing hyperkalemia incidence. Additionally, ACEI/ARB + MRA + SGLT-2i (OR: 3.27; 95% CI: 1.65–6.49), ACEI/ARB + RI (OR: 2.29; 95% CI: 1.15–4.57), ACEI/ARB + SGLT-2i (OR: 4.21; 95% CI: 2.34–7.58), placebo (OR: 3.97; 95% CI: 1.37–11.51) and SGLT-2i (OR: 6.44; 95% CI: 1.91–21.75) were found to be more effective in reducing hyperkalemia incidence compared to ACEI/ARB + MRA. Notably, SGLT-2i demonstrated greater efficacy than ARB (OR: 3.72; 95% CI: 1.70–8.13) and ACEI (OR: 2.77; 95% CI: 1.02–7.51) in reducing the incidence of hyperkalemia.

**TABLE 1:** League table showing the results of the network meta-analysis comparing the effects (MD or OR) of all interventions and 95% CI on hyperkalemia (upper-right) and serum potassium (bottom-left) in individuals with DKD.

SGLT-2i	1.20 (0.07, 21.57)	1.62 (0.92, 2.87)	2.84 (0.30, 26.75)	3.72 (1.70, 8.13)	1.53 (0.47, 5.03)	2.81 (0.83, 9.55)	1.97 (0.51, 7.56)	6.44 (1.91, 21.75)	2.12 (0.68, 6.60)	4.12 (1.43, 11.87)	2.77 (1.02, 7.51)
——	RI	1.35 (0.08, 23.04)	2.37 (0.07, 81.31)	3.10 (0.18, 52.74)	1.28 (0.06, 25.17)	2.35 (0.12, 46.9)	1.64 (0.08, 34.45)	5.38 (0.27, 106.62)	1.77 (0.09, 34.05)	3.44 (0.18, 64.39)	2.31 (0.12, 42.88)
——	0.01 (−0.16, 0.17)	Placebo	1.75 (0.20, 15.25)	**2.29 (1.34, 3.92)**	0.94 (0.33, 2.68)	1.73 (0.59, 5.12)	1.21 (0.36, 4.08)	**3.97 (1.37, 11.51)**	1.30 (0.49, 3.48)	**2.54 (1.04, 6.18)**	1.71 (0.75, 3.87)
——	−0.16 (−0.41, 0.09)	−0.17 (−0.38, 0.04)	MRA	1.31 (0.16, 10.97)	0.54 (0.07, 4.13)	0.99 (0.13, 7.82)	0.69 (0.09, 5.55)	2.27 (0.31, 16.33)	0.74 (0.10, 5.48)	1.45 (0.19, 11.02)	0.97 (0.12, 7.96)
——	−0.11 (−0.28, 0.06)	−0.12 (−0.27, 0.03)	0.05 (−0.16, 0.26)	ARB	0.41 (0.16, 1.07)	0.76 (0.28, 2.05)	0.53 (0.17, 1.64)	1.73 (0.66, 4.58)	0.57 (0.24, 1.38)	1.11 (0.51, 2.42)	0.75 (0.34, 1.64)
——	−0.14 (−0.37, 0.09)	−0.15 (−0.35, 0.05)	0.02 (−0.19, 0.24)	−0.03 (−0.21, 0.15)	ACEI/ARB+SGLT-2i	1.84 (0.99, 3.40)	1.29 (0.57, 2.88)	**4.21 (2.34, 7.58)**	1.38 (0.93, 2.06)	**2.69 (1.44, 5.02)**	1.81 (0.68, 4.80)
——	−0.21 (−0.55, 0.14)	−0.21 (−0.54, 0.11)	−0.04 (−0.38, 0.29)	−0.10 (−0.41, 0.22)	−0.07 (−0.36, 0.23)	ACEI/ARB+RI	0.70 (0.29, 1.70)	**2.29 (1.15, 4.57)**	0.75 (0.46, 1.24)	1.46 (0.73, 2.92)	0.98 (0.36, 2.73)
——	**−0.33 (**−**0.58,** −**0.08)**	**−0.34 (**−**0.56,** −**0.12)**	−0.17 (−0.40, 0.06)	**−0.22 (**−**0.43,** −**0.02)**	**−0.19 (**−**0.36,** −**0.03)**	−0.13 (−0.44, 0.18)	ACEI/ARB+MRA+SGLT-2i	**3.27 (1.65, 6.49)**	1.08 (0.53, 2.17)	2.09 (0.89, 4.92)	1.41 (0.44, 4.46)
——	**−0.32 (**−**0.51,** −**0.12)**	**−0.33 (**−**0.48,** −**0.17)**	−0.16 (−0.33, 0.02)	**−0.21 (**−**0.34,** −**0.07)**	**−0.18 (**−**0.30,** −**0.06)**	−0.11 (−0.40, 0.17)	0.02 (−0.14, 0.17)	ACEI/ARB+MRA	0.33 (0.22, 0.49)	0.64 (0.34, 1.20)	0.43 (0.16, 1.16)
——	−0.12 (−0.33, 0.09)	−0.12 (−0.30, 0.05)	0.05 (−0.15, 0.24)	−0.01 (−0.16, 0.15)	0.02 (−0.08, 0.13)	0.09 (−0.18, 0.36)	**0.22 (0.07, 0.37)**	**0.20 (0.12, 0.28)**	ACEI/ARB	**1.95 (1.19, 3.18)**	1.31 (0.53, 3.25)
——	**−0.30 (**−**0.55,** −**0.06)**	**−0.31 (**−**0.53,** −**0.09)**	−0.14 (−0.38, 0.09)	−0.20 (−0.40, 0.01)	−0.16 (−0.34, 0.01)	−0.10 (−0.41, 0.21)	0.03 (−0.18, 0.23)	0.01 (−0.14, 0.17)	**−0.19 (**−**0.33,** −**0.05)**	ACEI+ARB	0.67 (0.30, 1.51)
——	−0.13 (−0.31, 0.04)	**−0.14 (-0.23,** −**0.05)**	0.03 (−0.18, 0.23)	−0.03 (−0.17, 0.12)	0.01 (−0.18, 0.20)	0.07 (−0.25, 0.39)	0.20 (−0.01, 0.41)	**0.18 (0.04, 0.33)**	−0.02 (−0.18, 0.15)	0.17 (−0.04, 0.38)	ACEI

The values upper-right the therapeutic drugs correspond to the OR and 95% CI in the incidence of hyperkalemia between the row and columns (e.g., the OR in the incidence of hyperkalemia between ACEI/ARB + MRA and ACEI/ARB + MRA + SGLT-2i is 3.27). The values bottom-left the therapeutic drugs correspond to the MD and 95% CI in serum potassium levels (mmol/L) between the column and the row (e.g., the MD serum potassium levels between ACEI/ARB + MRA and ACEI/ARB is 0.2 mmol/L). Bold values indicate comparisons with significant differences.

In terms of lowering serum potassium levels, ACEI/ARB + SGLT-2i (MD: −0.19 mmol/L; 95% CI: −0.36 to −0.03), ACEI/ARB (MD: 0.22 mmol/L; 95% CI: 0.07–0.37), ARB (MD: −0.22 mmol/L; 95% CI: −0.43 to −0.02), and RI (MD: −0.33 mmol/L; 95% CI: −0.58 to −0.08) were more effective compared to ACEI/ARB + MRA + SGLT-2i. Similarly, ACEI/ARB + SGLT-2i (MD: −0.18 mmol/L; 95% CI: −0.30 to −0.06), ACEI/ARB (MD: 0.20 mmol/L; 95% CI: 0.12–0.28), ACEI (MD: 0.18 mmol/L; 95% CI: 0.04–0.33), ARB (MD: −0.21 mmol/L; 95% CI: −0.34 to −0.07), and RI (MD: −0.32 mmol/L; 95% CI: −0.51 to −0.12) achieved lower potassium levels compared to ACEI/ARB + MRA. Furthermore, ACEI or ARB alone (MD: −0.19 mmol/L; 95% CI: −0.33 to −0.05) were more effective in reducing serum potassium levels than the combination of ACEI + ARB.

### 3.8 SUCRA

**FIGURE 3:**
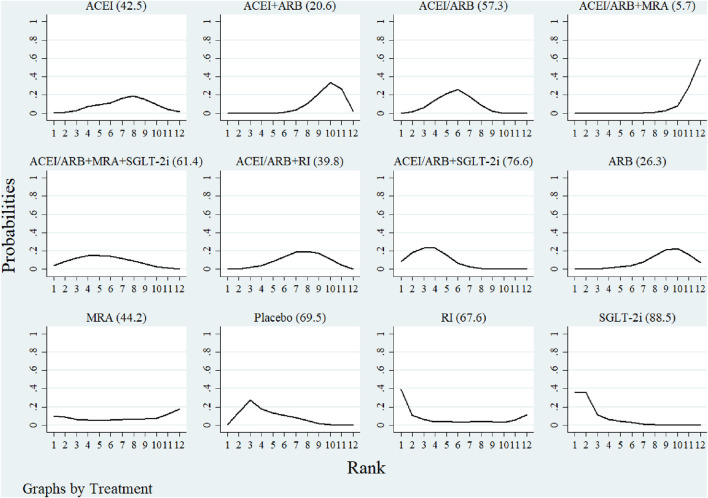
Ranking of hyperkalemia by SUCRA. The larger the SUCRA value, the better the result.

**FIGURE 4:**
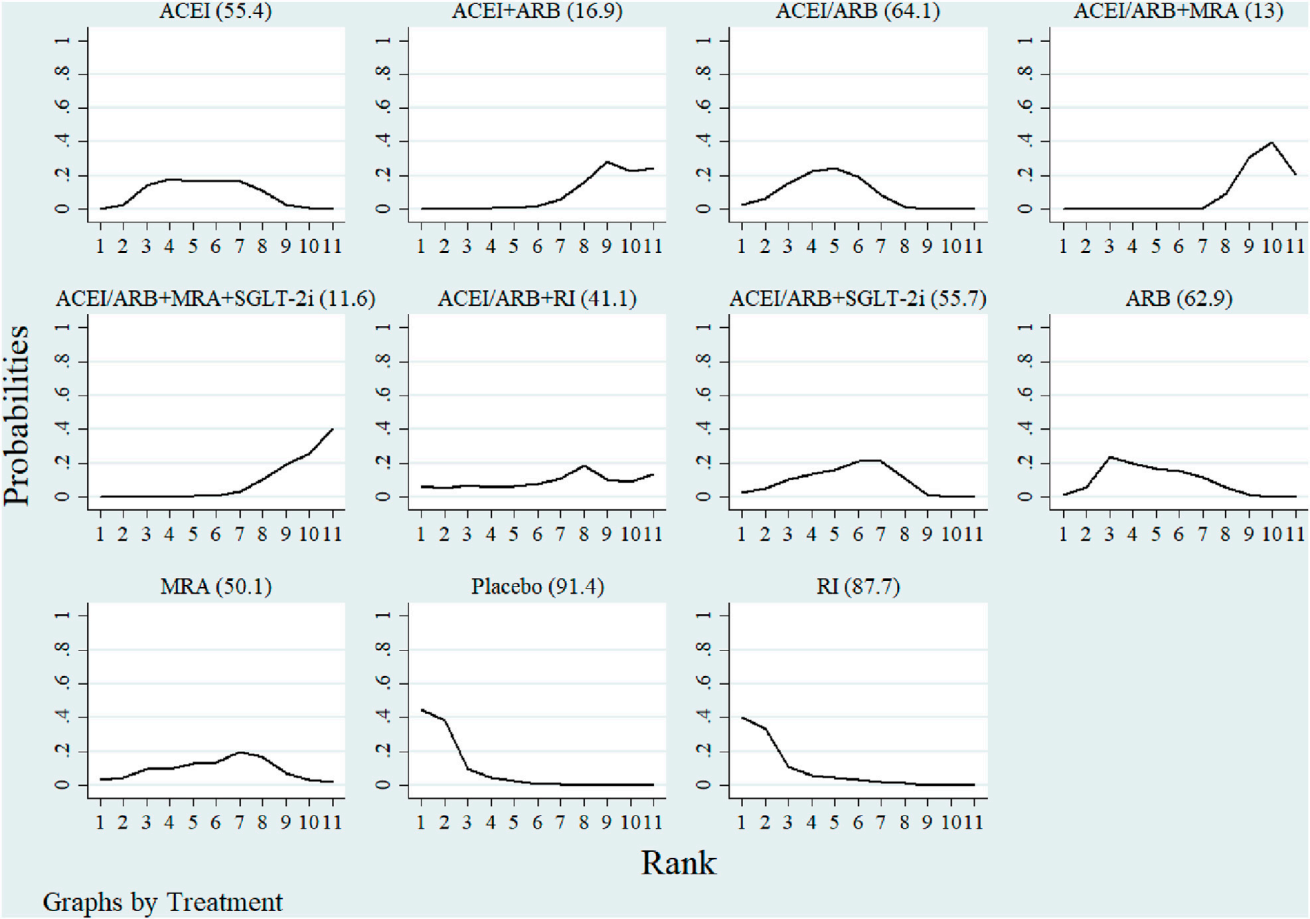
Ranking of serum potassium by SUCRA. The larger the SUCRA value, the better the result.

Figures 3, 4 showed the SUCRA values for hyperkalemia incidence and serum potassium levels. In terms of the risk of hyperkalemia, SGLT-2i (88.5%), ACEI/ARB + SGLT-2i (76.6%), and RI (67.6%) were identified as the top three most beneficial interventions. Regarding serum potassium levels, the placebo had the highest probability of being the most effective (91.4%), followed by RI (87.7%), and then ARB (62.9%).

### 3.9 Subgroup analyses

We conducted subgroup analyses to study the effect of eGFR, study duration, sample size, and mean age. The results of these subgroup analyses were consistent with the main analyses (Supplementary Tables S8–S10). Furthermore, a subgroup analysis on MRA was performed to evaluate hyperkalemia incidence, presented in Supplementary Table S11. The findings indicated that when used in combination with ACEI or ARB, like spironolactone (OR: 0.06; 95% CI: 0.02–0.23), esaxerenone (OR: 0.23; 95% CI: 0.12–0.42), and finerenone (OR: 0.45; 95% CI: 0.40–0.51), there was an increased frequency of hyperkalemia compared to ACEI or ARB alone. Spironolactone showed the least effectiveness in reducing the incidence of hyperkalemia. Moreover, the concurrent use of finerenone and ACEI or ARB was found to significantly decrease the risk of hyperkalemia when compared to spironolactone with ACEI/ARB (OR 0.14, 95% CI: 0.04–0.52).

### 3.10 Publication bias

There was no asymmetry in the funnel plots for either hyperkalemia or serum potassium levels (Supplementary Figures S10, S11).

### 3.11 Confidence of evidence

The confidence of evidence was low or very low for most of the comparisons regarding the risk of hyperkalemia (Supplementary Table S12). The confidence of evidence was moderate or very low regarding the level of serum potassium level (Supplementary Table S13).

## 4 Discussion

### 4.1 Principal findings

The NMA was used to investigate the effect of therapeutic drugs (ACEI, ARB, ACEI/ARB, ACEI + ARB, SGLT-2i, RI, MRA, Placebo, ACEI/ARB + SGLT-2i, ACEI/ARB + RI, ACEI/ARB + MRA, ACEI/ARB + MRA + SGLT-2i) on serum potassium levels in individuals with DKD. Results from this study demonstrated that ACEI/ARB combined with MRA was the least effective in reducing the incidence of hyperkalemia, whereas SGLT-2i emerged as the most effective drug. Interestingly, SGLT-2i even showed potential in partially counteracting the elevated risk associated with MRA combination therapy. Similarly, combined use of ACEI/ARB along with MRA resulted in a significant rise in serum potassium levels. Our findings offer valuable insights into the intricate interplay between drug classes and their impact on electrolyte homeostasis. Nevertheless, the confidence of the evidence ranged from very low to moderate for many of the comparisons, highlighting the necessity for additional high-quality studies to strengthen its credibility.

### 4.2 Comparison with previous studies and reasons for difference

Of note, combined use of ACEI and ARB elevated the incidence of hyperkalemia and the level serum potassium in patients with DKD when compared to using ACEI or ARB alone. Likewise, the previous studies found ACEI plus ARB dual therapy may result in a greater likelihood of hyperkalemia compared to using either medication alone (Whitlock et al., 2023; Nishida et al., 2012). The important mechanisms leading to hyperkalemia while on ACEI or ARB include decreased aldosterone levels, reduced sodium delivery to the distal nephron, impaired collecting duct function, and excessive potassium intake (Raebel, 2012). Therefore, it is not recommended to use ACEI and ARB together.

When RI aliskiren is combined with an ACEI or ARB, there is a notable rise in the likelihood of hyperkalemia (Parving et al., 2012). Yet, our research discovered that ACEI/ARB plus RI had a lower risk of hyperkalemia when compared to combining ACEI/ARB with MRA. This aligns with the finding from a study that observed no elevated risk of hospitalization due to hyperkalemia among elderly patients (aged 66 and older) receiving treatment with either ACEi or ARB, when aliskiren was used (Gilbert et al., 2013). In addition, small fluctuations in serum potassium levels were typically minor and not likely to have a meaningful impact on patients with heart failure or CKD (Weir and Rolfe, 2010). Conversely, significant decreases in serum potassium levels were observed with RI alone in this study. The different effects of RI and MRA on hyperkalemia could be attributed to different mechanisms of action. RI disrupts angiotensin II-induced stimulation of aldosterone secretion, leading to an increase in serum potassium levels, while MRA blocks the interaction between aldosterone and its receptor (Weir and Rolfe, 2010). Moreover, it is important to interpret these results cautiously, as only one article was available for RI alone. Therefore, further research is needed to confirm these findings and to fully understand the mechanisms underlying the effects of RI and MRA on serum potassium levels and hyperkalemia risk in patients with DKD.

The results of this study indicated that SGLT-2i alone had the lowest risk of hyperkalemia in patients with DKD, which is consistent with previous studies (Luo et al., 2023; Yang et al., 2022). The combination of ACEI/ARB and SGLT-2i was found to be linked to a reduced risk of hyperkalemia in comparison to ACEI plus ARB or ACEI/ARB plus MRA and did not increase the incidence of hyperkalemia compared to placebo, in line with previous research (Kaze et al., 2022; Pozzi et al., 2024). Previous research has shown that MRA added to SGLT-2i reduced the risk of hyperkalemia in patients with heart failure and reduced ejection fraction compared to MRA alone (Ahmed et al., 2023; Kristensen et al., 2020). The risk of hyperkalemia was significantly lower with the combination of ACEI/ARB and SGLT-2i plus MRA compared to ACEI/ARB plus MRA, suggesting a reversal of hyperkalemia occurrence in DKD patients by SGLT-2i, which is supported by a meta-analysis (Luo et al., 2023). However, SGLT-2i did not reverse absolute changes in serum potassium induced by the concurrent use of ACEI/ARB plus MRA. Possible reasons for this inconsistency could be related to differences in sample sizes (44249 for hyperkalemia and 17877 for serum potassium) and heterogeneity between studies. Therefore, a more thorough evaluation of individual risk factors and appropriate management strategies may be necessary in clinical practice. Additional investigation is necessary to ascertain if SGLT-2i can counteract the effects of MRA on serum potassium levels. SGLT-2i regulate potassium levels in a unique manner when compared to ACEI, ARB, or MRA. These mechanisms involve increased negative charge in the renal tubules (De La Flor et al., 2023; Neuen et al., 2022), a slight elevation in aldosterone levels (Lytvyn et al., 2022), and long-term sustained use of SGLT-2i for kidney function preservation, which could explain their counterbalancing effect on the risk of drug-induced hyperkalemia. These findings underscore the potential of SGLT-2i as a promising therapeutic option for DKD patients, not only for their well-established renal and cardiovascular benefits but also for their favorable effect on potassium homeostasis.

Our research revealed that the concomitant use of ACEI/ARB along with MRA posed the highest risk of hyperkalemic adverse events in patients with DKD. MRA alone did not increase the risk of hyperkalemia, but when combined with ACEI/ARB, it significantly elevated the risk, consistent with previous research (Luo et al., 2023). The similar results were observed in the absolute changes in serum potassium. The significant elevation in the risk of hyperkalemia observed with ACEI/ARB plus MRA highlights the need for careful monitoring and individualized dosing strategies when employing this combination therapy. Subgroup analyses on MRA indicated that the concurrent use of ACEI/ARB plus spironolactone was the most pronounced in increasing the risk of hyperkalemia. A previous study has shown that the risk of hyperkalemia was elevated by ACEI/ARB together with spironolactone, necessitating careful monitoring of patients’ serum potassium levels (Liu et al., 2018). Novel non-steroidal MRAs were associated with a lower risk of hyperkalemia compared to steroidal MRAs due to various factors such as tissue distribution, receptor inactivation, drug half-life and other factors (Barrera-Chimal et al., 2022). The previous study found that combination therapy with SGLT-2i could lower the hyperkalemia risk associated with finerenone (Agarwal et al., 2022; Shaikh et al., 2022). The SGLT-2i could counteract the hyperkalemia induced by ACEI/ARB plus finerenone or esaxerenone in this study. Therefore, clinicians should consider these findings when selecting the appropriate therapy for individual patients, balancing the potential benefits and risks of each treatment option.

Our study systematically assessed the impact of SGLT-2i and RAAS inhibitors on the incidence of hyperkalemia in patients with DKD, providing a detailed comparison of different drug combinations. This meta-analysis clearly reveals the effectiveness of SGLT-2i in reducing the risk of hyperkalemia, contrasting sharply with the increased risk of hyperkalemia associated with traditional RAAS inhibitor combinations. Although recent research also addresses the use of these medications, our study offers a more comprehensive and precise ranking of drug effects by including multiple randomized controlled trials and employing a network meta-analysis approach. Furthermore, our study particularly highlights the significant increase in the risk of hyperkalemia when using the ACEI/ARB + MRA combination, especially when combined with SGLT-2i, which was found to have a protective effect against hyperkalemia. This finding provides crucial evidence for the selection of drug combinations in clinical practice and offers a new perspective for optimizing treatment strategies for patients with DKD.

### 4.3 Strengths and limitations of the study

NMA methods, which are a combination of direct and indirect comparison simultaneously, were used in this systematic review. Another strength included identifying inconsistencies and assessing the confidence of evidence. Moreover, this study demonstrated that SGLT-2i presented a reduced risk of hyperkalemia and partially counteracted the hyperkalemia induced by the concomitant use of ACEI/ARB along with MRA in individuals with DKD. These findings are valuable for evidence-based decision-making in managing individuals with DKD.

The primary limitation of this review was the relatively small number and quality of included studies, with only 36 trials and 10 assessed at low risk of bias. Another significant limitation was the varying definitions of hyperkalemia in the original literature, with some studies using thresholds of over 5.5 mmol/L and others over 6 mmol/L. In addition, while our results contribute significantly to the understanding of drug effects on potassium levels in DKD, the statistical inconsistency in hyperkalemia, which also presented in the GRADE rating, reducing the confidence in the effect estimates and rankings. Consequently, our findings should be interpreted with caution and serve as a catalyst for further high-quality research in this area.

Our findings suggest that SGLT-2i is effective in reducing the incidence of hyperkalemia in individuals with DKD, whereas combinations of ACEI/ARB and MRA may elevate this risk. However, these pharmacological effects must be considered alongside the social determinants of health and social capital that influence disease management (Costa et al., 2022). Therefore, future research should prioritize the integration of social factors into clinical trials and observational studies to provide a more holistic understanding of DKD management.

### 4.4 Conclusion

SGLT-2i was found to be the most effective therapeutic drugs for lowering the risk of hyperkalemia in individuals with DKD, while MRA has been linked to a heightened risk of hyperkalemia. The potential of SGLT-2i to reverse the elevation of serum potassium induced by ACEI/ARB combined with MRA is still under investigation. The study found no significant evidence of transitivity or inconsistency in the clinical and methodological heterogeneity across the treatment comparisons, suggesting that the observed effects are robust. However, the confidence in the evidence for hyperkalemia risk was generally low or very low, and moderate to very low for serum potassium levels, indicating that further research is needed to strengthen these findings.

## Data Availability

The original contributions presented in the study are included in the article/Supplementary Material, further inquiries can be directed to the corresponding author.
