# Supplementary material for: Incidence of hyperkalemia RAASi and SGLT-2i treatment in individuals with diabetic kidney disease: a systematic review and network meta-analysis
Source: Front Pharmacol. 2025 Jan 27;15:1462965. doi: 10.3389/fphar.2024.1462965 (PMC11808247; doi:10.3389/fphar.2024.1462965)
Supplement: Supplementary file 1 [file DataSheet1.docx]

**Supplementary**

**Supplementary Table S1: Search terms**

| **Pubmed** | |
| --- | --- |
| #1 | (((((((((Renin inhibitors[Title/Abstract]) OR (Renin[Title/Abstract])) OR (aliskiren[Title/Abstract])) OR (rasilez[Title/Abstract])) OR (Tekturna[Title/Abstract])) OR (ciprokiren[Title/Abstract])) OR (ditekiren[Title/Abstract])) OR (enalkiren[Title/Abstract])) OR (remikiren[Title/Abstract])) OR (terlakiren[Title/Abstract]) |
| #2 | (((((((((((((((((((((Angiotensin-Converting Enzyme Inhibitors[MeSH Terms]) OR (Angiotensin Converting Enzyme Inhibitors[Title/Abstract])) OR (Enzyme Inhibitors, Angiotensin-Converting[Title/Abstract])) OR (Inhibitor, Angiotensin-Converting Enzyme[Title/Abstract])) OR (Inhibitor, Angiotensin Converting Enzyme[Title/Abstract])) OR (Inhibitor, Kininase II[Title/Abstract])) OR (Kininase II Antagonists[Title/Abstract])) OR (Kininase II Inhibitor[Title/Abstract])) OR (Angiotensin-Converting Enzyme Antagonists[Title/Abstract])) OR (Angiotensin Converting Enzyme Antagonists[Title/Abstract])) OR (Enzyme Antagonists, Angiotensin-Converting[Title/Abstract])) OR (II Inhibitor, Kininase[Title/Abstract])) OR (Antagonists, Kininase II[Title/Abstract])) OR (Inhibitor, ACE[Title/Abstract])) OR (ACE Inhibitor[Title/Abstract])) OR (Angiotensin I-Converting Enzyme Inhibitor[Title/Abstract])) OR (Angiotensin I Converting Enzyme Inhibitor[Title/Abstract])) OR (Angiotensin Converting Enzyme Inhibitor[Title/Abstract])) OR (Angiotensin-Converting Enzyme Inhibitor[Title/Abstract])) OR (Enzyme Inhibitor, Angiotensin-Converting[Title/Abstract])) OR (Antagonists, Angiotensin-Converting Enzyme[Title/Abstract])) OR (Antagonists, Angiotensin Converting Enzyme[Title/Abstract]) |
| #3 | (((((((((((((((((((((((((((((((((((((((((((((((((((((((((((((((((((((((((((((((((((Captopril[Title/Abstract]) OR (Capoten[Title/Abstract])) OR (Enalapril[Title/Abstract])) OR (Renitec[Title/Abstract])) OR (Renitek[Title/Abstract])) OR (Enalapril Maleate[Title/Abstract])) OR (Lisinopril[Title/Abstract])) OR (Lysinopril[Title/Abstract])) OR (Prinivil[Title/Abstract])) OR (Zestril[Title/Abstract])) OR (Perindopril[Title/Abstract])) OR (Pirindopril[Title/Abstract])) OR (Perindopril[Title/Abstract])) OR (Erbumine[Title/Abstract])) OR (Perstarium[Title/Abstract])) OR (Quinapril[Title/Abstract])) OR (Accupril[Title/Abstract])) OR (Quinapril Hydrochloride[Title/Abstract])) OR (Ramipril[Title/Abstract])) OR (Vesdil[Title/Abstract])) OR (Triatec[Title/Abstract])) OR (Altace[Title/Abstract])) OR (Ramace[Title/Abstract])) OR (Tritace[Title/Abstract])) OR (Acovil[Title/Abstract])) OR (Delix[Title/Abstract])) OR (Carasel[Title/Abstract])) OR (Delapril[Title/Abstract])) OR (Derapril[Title/Abstract])) OR (Benazepril[Title/Abstract])) OR (benzazepril[Title/Abstract])) OR (Labopal[Title/Abstract])) OR (Lotensin[Title/Abstract])) OR (Cibacen[Title/Abstract])) OR (Briem[Title/Abstract])) OR (benazepril hydrochloride[Title/Abstract])) OR (cilazapril[Title/Abstract])) OR (Cilazapril Monohydrobromide[Title/Abstract])) OR (Cilazapril, Anhydrous[Title/Abstract])) OR (Cilazapril Anhydrous[Title/Abstract])) OR (Cilazapril Monohydrate[Title/Abstract])) OR (Cilazapril Hydrate[Title/Abstract])) OR (Inhibace[Title/Abstract])) OR (Fosinopril[Title/Abstract])) OR (Fosenopril[Title/Abstract])) OR (Fosinil[Title/Abstract])) OR (Fosinopril Sodium[Title/Abstract])) OR (Tenso Stop[Title/Abstract])) OR (Monopril[Title/Abstract])) OR (Fosinorm[Title/Abstract])) OR (Newace[Title/Abstract])) OR (Staril[Title/Abstract])) OR (Moexipril[Title/Abstract])) OR (Fempress[Title/Abstract])) OR (moexipril hydrochloride[Title/Abstract])) OR (Univasc[Title/Abstract])) OR (Moex[Title/Abstract])) OR (Perdix[Title/Abstract])) OR (Spirapril[Title/Abstract])) OR (spirapril hydrochloride[Title/Abstract])) OR (Renpress[Title/Abstract])) OR (Quadropril[Title/Abstract])) OR (Pentopril[Title/Abstract])) OR (Trandolapril[Title/Abstract])) OR (Odrik[Title/Abstract])) OR (Mavik[Title/Abstract])) OR (Gopten[Title/Abstract])) OR (Temocapril[Title/Abstract])) OR (Imidapril[Title/Abstract])) OR (imidapril hydrochloride[Title/Abstract])) OR (alacepril[Title/Abstract])) OR (aracepril[Title/Abstract])) OR (altiopril[Title/Abstract])) OR (ceranapril[Title/Abstract])) OR (idrapril[Title/Abstract])) OR (indolapril[Title/Abstract])) OR (Indolapril hydrochloride[Title/Abstract])) OR (Libenzapril[Title/Abstract])) OR (Rentiapril[Title/Abstract])) OR (Utibapril[Title/Abstract])) OR (Zabicipril[Title/Abstract])) OR (Zofenopril[Title/Abstract])) OR (Zofenil[Title/Abstract])) OR (Tanatril[Title/Abstract]) |
| #4 | ((((((((Angiotensin Receptor Antagonists[MeSH Terms]) OR (Antagonist, Angiotensin Receptor[Title/Abstract])) OR (Receptor Antagonist, Angiotensin[Title/Abstract])) OR (Angiotensin Receptor Blocker[Title/Abstract])) OR (Receptor Blocker, Angiotensin[Title/Abstract])) OR (Blocker, Angiotensin Receptor[Title/Abstract])) OR (Angiotensin Receptor Antagonist[Title/Abstract])) OR (Angiotensin II Receptor Antagonist[Title/Abstract])) OR (Angiotensin II Receptor Blocker[Title/Abstract]) |
| #5 | ((((((((((((((((((((((((((((((((Azilsartan[Title/Abstract]) OR (Candesartan[Title/Abstract])) OR (Eprosartan[Title/Abstract])) OR (Teveten[Title/Abstract])) OR (Irbesartan[Title/Abstract])) OR (Avapro[Title/Abstract])) OR (Karvea[Title/Abstract])) OR (Aprovel[Title/Abstract])) OR (Losartan[Title/Abstract])) OR (Cozaar[Title/Abstract])) OR (Losartan Potassium[Title/Abstract])) OR (Potassium[Title/Abstract])) OR (Losartan[Title/Abstract])) OR (Losartan Monopotassium Salt[Title/Abstract])) OR (Monopotassium Salt[Title/Abstract])) OR (Losartan[Title/Abstract])) OR (Salt, Losartan Monopotassium[Title/Abstract])) OR (Olmesartan[Title/Abstract])) OR (omesartan[Title/Abstract])) OR (tasosartan[Title/Abstract])) OR (Telmisartan[Title/Abstract])) OR (Pritor[Title/Abstract])) OR (Micardis[Title/Abstract])) OR (Valsartan[Title/Abstract])) OR (Diovan[Title/Abstract])) OR (Tareg[Title/Abstract])) OR (Nisis[Title/Abstract])) OR (Provas[Title/Abstract])) OR (Vals[Title/Abstract])) OR (saprisartan potassium[Title/Abstract])) OR (pratosartan[Title/Abstract])) OR (milfasartan[Title/Abstract])) OR (fimasartan[Title/Abstract]) |
| #6 | ((((((((((Mineralocorticoid Receptor Antagonists[MeSH Terms]) OR (Antagonist, Mineralocorticoid Receptor[Title/Abstract])) OR (Receptor Antagonist, Mineralocorticoid[Title/Abstract])) OR (Mineralocorticoid Antagonist[Title/Abstract])) OR (Antagonist, Mineralocorticoid[Title/Abstract])) OR (Aldosterone Receptor Antagonist[Title/Abstract])) OR (Antagonist, Aldosterone Receptor[Title/Abstract])) OR (Receptor Antagonist, Aldosterone[Title/Abstract])) OR (Mineralocorticoid Receptor Antagonist[Title/Abstract])) OR (Aldosterone Antagonist[Title/Abstract])) OR (Antagonist, Aldosterone[Title/Abstract]) |
| #7 | (((((((((((((((((((Finerenone[Title/Abstract]) OR (kerendia[Title/Abstract])) OR (BAY 94-8862[Title/Abstract])) OR (esaxerenone[Title/Abstract])) OR (CS-3150[Title/Abstract])) OR (Apararenone[Title/Abstract])) OR (MT-3995[Title/Abstract])) OR (Eplerenone[Title/Abstract])) OR (Eplerenon[Title/Abstract])) OR (Inspra[Title/Abstract])) OR (Spironolactone[Title/Abstract])) OR (Spirolactone[Title/Abstract])) OR (Veroshpiron[Title/Abstract])) OR (Verospirone[Title/Abstract])) OR (Spiractin[Title/Abstract])) OR (Spirogamma[Title/Abstract])) OR (Spirolang[Title/Abstract])) OR (Aldactone[Title/Abstract])) OR (Verospiron[Title/Abstract])) OR (Aldactone A[Title/Abstract]) |
| #8 | (((((((((((((Sodium-Glucose Transporter 2 Inhibitors[MeSH Terms]) OR (Sodium Glucose Transporter 2 Inhibitors[Title/Abstract])) OR (SGLT-2 Inhibitors[Title/Abstract])) OR (SGLT 2 Inhibitors[Title/Abstract])) OR (SGLT2 Inhibitors[Title/Abstract])) OR (Sodium-Glucose Transporter 2 Inhibitor[Title/Abstract])) OR (Sodium Glucose Transporter 2 Inhibitor[Title/Abstract])) OR (SGLT2 Inhibitor[Title/Abstract])) OR (Inhibitor, SGLT2[Title/Abstract])) OR (Gliflozins[Title/Abstract])) OR (Gliflozin[Title/Abstract])) OR (SGLT-2 Inhibitor[Title/Abstract])) OR (Inhibitor, SGLT-2[Title/Abstract])) OR (SGLT 2 Inhibitor[Title/Abstract]) |
| #9 | ((((((((((((((((((((((Dapagliflozin[Title/Abstract]) OR (Farxiga[Title/Abstract])) OR (Forxiga[Title/Abstract])) OR (Canagliflozin[Title/Abstract])) OR (Invokana[Title/Abstract])) OR (Canagliflozin Hemihydrate[Title/Abstract])) OR (Canagliflozin, Anhydrous[Title/Abstract])) OR (Empagliflozin[Title/Abstract])) OR (Jardiance[Title/Abstract])) OR (Ipragliflozin[Title/Abstract])) OR (Suglat[Title/Abstract])) OR (luseogliflozin[Title/Abstract])) OR (Lusefi[Title/Abstract])) OR (remogliflozin[Title/Abstract])) OR (Remogliflozin[Title/Abstract])) OR (Topogliflozin[Title/Abstract])) OR (Sergliflozin[Title/Abstract])) OR (ertugliflozin[Title/Abstract])) OR (tofogliflozin[Title/Abstract])) OR (Bexagliflozin[Title/Abstract])) OR (henagliflozin[Title/Abstract])) OR (licogliflozin[Title/Abstract])) OR (sotagliflozin[Title/Abstract]) |
| #10 | #1 OR #2 OR #3 OR #4 OR #5 OR #6 OR #7 OR #8 OR #9 |
| #11 | (((((((((Diabetic Nephropathies[MeSH Terms]) OR (Nephropathies, Diabetic[Title/Abstract])) OR (Nephropathy, Diabetic[Title/Abstract])) OR (Diabetic Nephropathy[Title/Abstract])) OR (Diabetic Kidney Disease[Title/Abstract])) OR (Diabetic Kidney Diseases[Title/Abstract])) OR (Kidney Disease, Diabetic[Title/Abstract])) OR (Kidney Diseases, Diabetic[Title/Abstract])) OR (Diabetic Glomerulosclerosis[Title/Abstract])) OR (Glomerulosclerosis, Diabetic[Title/Abstract]) |
| #12 | ((Diabetes Mellitus[Title/Abstract]) OR (Diabetes[Title/Abstract])) OR (Diabetic[Title/Abstract]) |
| #13 | (Albuminuria[Title/Abstract]) OR (Proteinuria[Title/Abstract]) |
| #14 | #12 AND #13 |
| #15 | #11 OR #14 |
| #16 | ((((((((((((randomized controlled trial[Publication Type]) OR (controlled clinical trial[Publication Type])) OR (randomized controlled trial[Title/Abstract])) OR (random[Title/Abstract])) OR (randomly[Title/Abstract])) OR (randomized[Title/Abstract])) OR (randomised[Title/Abstract])) OR (trial[Title/Abstract])) OR (clinical trial[Title/Abstract])) OR (Clinical Trials as Topic[Title/Abstract])) OR (RCT[Title/Abstract])) OR (RCTs[Title/Abstract])) OR (placebo[Title/Abstract]) |
| #17 | #10 AND #15 AND #16 |

| **Cochrane CENTRAL** | |
| --- | --- |
| #1 | (Renin inhibitors): ti,ab,kw OR (Renin inhibitors):ti,ab,kw OR (Renin):ti,ab,kw OR (aliskiren):ti,ab,kw OR (rasilez):ti,ab,kw OR (Tekturna):ti,ab,kw OR (ciprokiren):ti,ab,kw OR (ditekiren):ti,ab,kw OR (enalkiren):ti,ab,kw OR (remikiren):ti,ab,kw OR (terlakiren):ti,ab,kw |
| #2 | MeSH descriptor: [Angiotensin-Converting Enzyme Inhibitors] explode all trees |
| #3 | (Angiotensin Converting Enzyme Inhibitors):ti,ab,kw OR (Enzyme Inhibitors, Angiotensin-Converting):ti,ab,kw OR (Inhibitor, Angiotensin-Converting Enzyme):ti,ab,kw OR (Inhibitor, Angiotensin Converting Enzyme):ti,ab,kw OR (Inhibitor, Kininase II):ti,ab,kw OR (Kininase II Antagonists):ti,ab,kw OR (Kininase II Inhibitor):ti,ab,kw OR (Angiotensin-Converting Enzyme Antagonists):ti,ab,kw OR (Angiotensin Converting Enzyme Antagonists):ti,ab,kw OR (Enzyme Antagonists, Angiotensin-Converting):ti,ab,kw OR (II Inhibitor, Kininase):ti,ab,kw OR (Antagonists, Kininase II):ti,ab,kw OR (Inhibitor, ACE):ti,ab,kw OR (ACE Inhibitor):ti,ab,kw OR ("angiotensin I-converting enzyme inhibitor"):ti,ab,kw OR ("angiotensin I converting enzyme inhibitor"):ti,ab,kw OR ("angiotensin converting enzyme inhibitor"):ti,ab,kw OR ("angiotensin-converting enzyme inhibitor"):ti,ab,kw OR (Enzyme Inhibitor, Angiotensin-Converting):ti,ab,kw OR (Antagonists, Angiotensin-Converting Enzyme):ti,ab,kw OR (Antagonists, Angiotensin Converting Enzyme):ti,ab,kw |
| #4 | (Captopril):ti,ab,kw OR (Capoten):ti,ab,kw OR (Enalapril):ti,ab,kw OR (Renitec):ti,ab,kw OR (Renitek):ti,ab,kw OR ("enalapril maleate"):ti,ab,kw OR (Lisinopril):ti,ab,kw OR ("lysinopril"):ti,ab,kw OR (Prinivil):ti,ab,kw OR (Zestril):ti,ab,kw OR ("perindopril"):ti,ab,kw OR ("pirindopril"):ti,ab,kw OR (Perindopril Erbumine):ti,ab,kw OR (Perstarium):ti,ab,kw OR ("quinapril"):ti,ab,kw OR (Accupril):ti,ab,kw OR (Quinapril Hydrochloride):ti,ab,kw OR ("ramipril"):ti,ab,kw OR (Vesdil):ti,ab,kw OR (Triatec):ti,ab,kw OR (Altace):ti,ab,kw OR (Ramace):ti,ab,kw OR (Tritace):ti,ab,kw OR (Acovil):ti,ab,kw OR (Delix):ti,ab,kw OR (Carasel):ti,ab,kw OR ("delapril"):ti,ab,kw OR ("derapril"):ti,ab,kw OR ("benazepril"):ti,ab,kw OR (benzazepril):ti,ab,kw OR (Labopal):ti,ab,kw OR ("lotensin"):ti,ab,kw OR (Cibacen):ti,ab,kw OR (Briem):ti,ab,kw OR (benazepril hydrochloride):ti,ab,kw OR (cilazapril):ti,ab,kw OR (Cilazapril Monohydrobromide):ti,ab,kw OR (Cilazapril, Anhydrous):ti,ab,kw OR (Cilazapril Anhydrous):ti,ab,kw OR (Cilazapril Monohydrate):ti,ab,kw OR (Cilazapril Hydrate):ti,ab,kw OR (Inhibace):ti,ab,kw OR ("fosinopril"):ti,ab,kw OR ("fosenopril"):ti,ab,kw OR (Fosinil):ti,ab,kw OR (Fosinopril Sodium):ti,ab,kw OR (Tenso Stop):ti,ab,kw OR (Monopril):ti,ab,kw OR (Fosinorm):ti,ab,kw OR (Newace):ti,ab,kw OR (Staril):ti,ab,kw OR (Moexipril):ti,ab,kw OR (Fempress):ti,ab,kw OR (moexipril hydrochloride):ti,ab,kw OR (Univasc):ti,ab,kw OR (Moex):ti,ab,kw OR ("Perdix"):ti,ab,kw OR ("spirapril"):ti,ab,kw OR (spirapril hydrochloride):ti,ab,kw OR (Renpress):ti,ab,kw OR (Quadropril):ti,ab,kw OR ("pentopril"):ti,ab,kw OR ("trandolapril"):ti,ab,kw OR (Odrik):ti,ab,kw OR (Mavik):ti,ab,kw OR (Gopten):ti,ab,kw OR ("temocapril"):ti,ab,kw OR ("imidapril"):ti,ab,kw OR (imidapril hydrochloride):ti,ab,kw OR (alacepril):ti,ab,kw OR (aracepril):ti,ab,kw OR (altiopril):ti,ab,kw OR (ceranapril):ti,ab,kw OR (idrapril):ti,ab,kw OR (indolapril):ti,ab,kw OR (Indolapril hydrochloride):ti,ab,kw OR ("libenzapril"):ti,ab,kw OR ("rentiapril"):ti,ab,kw OR ("utibapril"):ti,ab,kw OR ("zabicipril"):ti,ab,kw OR ("zofenopril"):ti,ab,kw OR (Zofenil):ti,ab,kw OR (Tanatril):ti,ab,kw |
| #5 | #2 OR #3 OR #4 |
| #6 | MeSH descriptor: [Angiotensin Receptor Antagonists] explode all trees |
| #7 | (Antagonist, Angiotensin Receptor):ti,ab,kw OR (Receptor Antagonist, Angiotensin):ti,ab,kw OR ("angiotensin receptor blocker"):ti,ab,kw OR ("angiotensin-receptor blocker"):ti,ab,kw OR ("angiotensin-receptor blockers"):ti,ab,kw OR (Receptor Blocker, Angiotensin):ti,ab,kw OR (Blocker, Angiotensin Receptor):ti,ab,kw OR (Angiotensin Receptor Antagonist):ti,ab,kw OR (Angiotensin II Receptor Antagonist):ti,ab,kw OR ("angiotensin II receptor blocker"):ti,ab,kw |
| #8 | (Azilsartan):ti,ab,kw OR ("candesartan"):ti,ab,kw OR ("eprosartan"):ti,ab,kw OR (Teveten):ti,ab,kw OR ("irbesartan"):ti,ab,kw OR (Avapro):ti,ab,kw OR (Karvea):ti,ab,kw OR (Aprovel):ti,ab,kw OR ("losartan"):ti,ab,kw OR ("COZAAR"):ti,ab,kw OR (Losartan Potassium):ti,ab,kw OR ("potassium"):ti,ab,kw OR ("losartan"):ti,ab,kw OR (Losartan Monopotassium Salt):ti,ab,kw OR (Monopotassium Salt):ti,ab,kw  OR (Losartan):ti,ab,kw OR (Salt, Losartan Monopotassium):ti,ab,kw OR ("olmesartan"):ti,ab,kw OR (tasosartan):ti,ab,kw OR ("telmisartan"):ti,ab,kw OR (Pritor):ti,ab,kw OR (Micardis):ti,ab,kw OR ("valsartan"):ti,ab,kw OR (Diovan):ti,ab,kw OR (Tareg):ti,ab,kw  OR (Nisis):ti,ab,kw OR (Provas):ti,ab,kw OR (Vals):ti,ab,kw OR (saprisartan potassium):ti,ab,kw OR (pratosartan):ti,ab,kw OR (milfasartan):ti,ab,kw OR (fimasartan):ti,ab,kw |
| #9 | #6 OR #7 OR #8 |
| #10 | MeSH descriptor: [Mineralocorticoid Receptor Antagonists] explode all trees |
| #11 | (Antagonist, Mineralocorticoid Receptor):ti,ab,kw OR (Receptor Antagonist, Mineralocorticoid):ti,ab,kw OR (Mineralocorticoid Antagonist):ti,ab,kw OR (Antagonist, Mineralocorticoid):ti,ab,kw OR (Aldosterone Receptor Antagonist):ti,ab,kw OR (Antagonist, Aldosterone Receptor):ti,ab,kw OR (Receptor Antagonist, Aldosterone):ti,ab,kw OR (Mineralocorticoid Receptor Antagonist):ti,ab,kw OR (Aldosterone Antagonist):ti,ab,kw OR (Antagonist, Aldosterone):ti,ab,kw |
| #12 | (Finerenone):ti,ab,kw OR (kerendia):ti,ab,kw OR (esaxerenone):ti,ab,kw OR (CS-3150):ti,ab,kw OR (Apararenone):ti,ab,kw OR (MT-3995):ti,ab,kw OR (Eplerenone):ti,ab,kw OR (Eplerenon):ti,ab,kw OR (Inspra):ti,ab,kw OR (Spironolactone):ti,ab,kw OR (Spirolactone):ti,ab,kw OR (Veroshpiron):ti,ab,kw OR (Verospirone):ti,ab,kw OR (Spiractin):ti,ab,kw OR (Spirogamma):ti,ab,kw OR (Spirolang):ti,ab,kw OR (Aldactone):ti,ab,kw OR (Verospiron):ti,ab,kw OR (Aldactone A):ti,ab,kw |
| #13 | #10 OR #11 OR #12 |
| #14 | MeSH descriptor: [Sodium-Glucose Transporter 2 Inhibitors] explode all trees |
| #15 | (Sodium Glucose Transporter 2 Inhibitors):ti,ab,kw OR (SGLT-2 Inhibitors):ti,ab,kw OR (SGLT 2 Inhibitors):ti,ab,kw OR (SGLT2 Inhibitors):ti,ab,kw OR (Sodium-Glucose Transporter 2 Inhibitor):ti,ab,kw OR (Sodium Glucose Transporter 2 Inhibitor):ti,ab,kw OR (SGLT2 Inhibitor):ti,ab,kw OR (Inhibitor, SGLT2):ti,ab,kw OR (sodium glucose transporter ii inhibitor):ti,ab,kw OR (Gliflozins):ti,ab,kw OR (Gliflozin):ti,ab,kw OR (SGLT-2 Inhibitor):ti,ab,kw OR (Inhibitor, SGLT-2):ti,ab,kw OR (SGLT 2 Inhibitor):ti,ab,kw OR (Dapagliflozin):ti,ab,kw OR (Farxiga):ti,ab,kw OR (Forxiga):ti,ab,kw OR (Canagliflozin):ti,ab,kw OR (Invokana):ti,ab,kw OR (Canagliflozin Hemihydrate):ti,ab,kw OR (Canagliflozin, Anhydrous):ti,ab,kw OR (Empagliflozin):ti,ab,kw OR (Jardiance):ti,ab,kw OR (Ipragliflozin):ti,ab,kw OR (Suglat):ti,ab,kw OR (luseogliflozin):ti,ab,kw OR (Lusefi):ti,ab,kw OR (remogliflozin):ti,ab,kw OR (Topogliflozin):ti,ab,kw OR (Sergliflozin):ti,ab,kw OR (ertugliflozin):ti,ab,kw OR (tofogliflozin):ti,ab,kw OR (Bexagliflozin):ti,ab,kw OR (henagliflozin):ti,ab,kw OR (licogliflozin):ti,ab,kw OR (sotagliflozin):ti,ab,kw |
| #16 | #14 OR #15 |
| #17 | #1 OR #5 OR #9 OR #13 OR #16 |
| #18 | MeSH descriptor: [Diabetic Nephropathies] explode all trees |
| #19 | (Nephropathy, Diabetic):ti,ab,kw OR (Nephropathies, Diabetic):ti,ab,kw OR ("diabetic nephropathy"):ti,ab,kw OR (Diabetic Kidney Disease):ti,ab,kw OR (Diabetic Kidney Diseases):ti,ab,kw OR (Kidney Disease, Diabetic):ti,ab,kw OR (Kidney Diseases, Diabetic):ti,ab,kw OR ("diabetic glomerulosclerosis"):ti,ab,kw OR (Glomerulosclerosis, Diabetic):ti,ab,kw |
| #20 | #18 OR #19 |
| #21 | (Diabetes Mellitus):ti,ab,kw OR (Diabete):ti,ab,kw OR ("diabetic"):ti,ab,kw |
| #22 | ("albuminuria"):ti,ab,kw OR ("proteinuria"):ti,ab,kw |
| #23 | #21 AND #22 |
| #24 | #20 OR #23 |
| #25 | (randomized controlled trial):ti,ab,kw OR (controlled clinical trial):ti,ab,kw OR ("randomized-controlled trial"):ti,ab,kw OR ("randomized-controlled trials"):ti,ab,kw OR (random):ti,ab,kw  OR (randomly):ti,ab,kw OR (randomized):ti,ab,kw OR (randomised):ti,ab,kw OR (trial):ti,ab,kw OR (clinical trial):ti,ab,kw  OR (Clinical Trials):ti,ab,kw OR (RCT):ti,ab,kw OR (RCTs):ti,ab,kw OR (placebo):ti,ab,kw |
| #26 | #17 AND #23 AND #25 |

| **Embase** | |
| --- | --- |
| #1 | 'renin inhibitors':ab,kw,ti OR 'renin':ab,kw,ti OR 'aliskiren':ab,kw,ti OR 'rasilez':ab,kw,ti OR 'tekturna':ab,kw,ti OR 'ciprokiren':ab,kw,ti OR 'ditekiren':ab,kw,ti OR 'enalkiren':ab,kw,ti OR 'remikiren':ab,kw,ti OR 'terlakiren':ab,kw,ti |
| #2 | 'angiotensin-converting enzyme inhibitors':ab,kw,ti OR 'angiotensin converting enzyme inhibitors':ab,kw,ti OR 'enzyme inhibitors, angiotensin-converting':ab,kw,ti OR 'inhibitor, angiotensin-converting enzyme':ab,kw,ti OR 'inhibitor, angiotensin converting enzyme':ab,kw,ti OR 'inhibitor, kininase ii':ab,kw,ti OR 'kininase ii antagonists':ab,kw,ti OR 'kininase ii inhibitor':ab,kw,ti OR 'angiotensin-converting enzyme antagonists':ab,kw,ti OR 'angiotensin converting enzyme antagonists':ab,kw,ti OR 'enzyme antagonists, angiotensin-converting':ab,kw,ti OR 'ii inhibitor, kininase':ab,kw,ti OR 'antagonists, kininase ii':ab,kw,ti OR 'inhibitor, ace':ab,kw,ti OR 'ace inhibitor':ab,kw,ti OR 'angiotensin i-converting enzyme inhibitor':ab,kw,ti OR 'angiotensin i converting enzyme inhibitor':ab,kw,ti OR 'angiotensin converting enzyme inhibitor':ab,kw,ti OR 'angiotensin-converting enzyme inhibitor':ab,kw,ti OR 'enzyme inhibitor, angiotensin-converting':ab,kw,ti OR 'antagonists, angiotensin-converting enzyme':ab,kw,ti OR 'antagonists, angiotensin converting enzyme':ab,kw,ti |
| #3 | 'captopril':ab,kw,ti OR 'capoten':ab,kw,ti OR 'enalapril':ab,kw,ti OR 'renitec':ab,kw,ti OR 'renitek':ab,kw,ti OR 'enalapril maleate':ab,kw,ti OR 'lisinopril':ab,kw,ti OR 'lysinopril':ab,kw,ti OR 'prinivil':ab,kw,ti OR 'zestril':ab,kw,ti OR 'perindopril':ab,kw,ti OR 'pirindopril':ab,kw,ti OR 'perindopril erbumine':ab,kw,ti OR 'perstarium':ab,kw,ti OR 'quinapril':ab,kw,ti OR 'accupril':ab,kw,ti OR 'quinapril hydrochloride':ab,kw,ti OR 'ramipril':ab,kw,ti OR 'vesdil':ab,kw,ti OR 'triatec':ab,kw,ti OR 'altace，ramace':ab,kw,ti OR 'tritace':ab,kw,ti OR 'acovil':ab,kw,ti OR 'delix':ab,kw,ti OR 'carasel':ab,kw,ti OR 'delapril':ab,kw,ti OR 'derapril':ab,kw,ti OR 'benazepril':ab,kw,ti OR 'benzazepril':ab,kw,ti OR 'labopal':ab,kw,ti OR 'lotensin':ab,kw,ti OR 'cibacen':ab,kw,ti OR 'briem':ab,kw,ti OR 'benazepril hydrochloride':ab,kw,ti OR 'cilazapril':ab,kw,ti OR 'cilazapril monohydrobromide':ab,kw,ti OR 'cilazapril, anhydrous':ab,kw,ti OR 'cilazapril anhydrous':ab,kw,ti OR 'cilazapril monohydrate':ab,kw,ti OR 'cilazapril hydrate':ab,kw,ti OR 'inhibace':ab,kw,ti OR 'fosinopril':ab,kw,ti OR 'fosenopril':ab,kw,ti OR 'fosinil':ab,kw,ti OR 'fosinopril sodium':ab,kw,ti OR 'tenso stop':ab,kw,ti OR 'monopril':ab,kw,ti OR 'fosinorm':ab,kw,ti OR 'newace':ab,kw,ti OR 'staril':ab,kw,ti OR 'moexipril':ab,kw,ti OR 'fempress':ab,kw,ti OR 'moexipril hydrochloride':ab,kw,ti OR 'univasc':ab,kw,ti OR 'moex':ab,kw,ti OR 'perdix':ab,kw,ti OR 'spirapril':ab,kw,ti OR 'spirapril hydrochloride':ab,kw,ti OR 'renpress':ab,kw,ti OR 'quadropril':ab,kw,ti OR 'pentopril':ab,kw,ti OR 'trandolapril':ab,kw,ti OR 'odrik':ab,kw,ti OR 'mavik':ab,kw,ti OR 'gopten':ab,kw,ti OR 'temocapril':ab,kw,ti OR 'imidapril':ab,kw,ti OR 'imidapril hydrochloride':ab,kw,ti OR 'alacepril':ab,kw,ti OR 'aracepril':ab,kw,ti OR 'altiopril':ab,kw,ti OR 'ceranapril':ab,kw,ti OR 'idrapril':ab,kw,ti OR 'indolapril':ab,kw,ti OR 'indolapril hydrochloride':ab,kw,ti OR 'libenzapril':ab,kw,ti OR 'rentiapril':ab,kw,ti OR 'utibapril':ab,kw,ti OR 'zabicipril':ab,kw,ti OR 'zofenopril':ab,kw,ti OR 'zofenil':ab,kw,ti OR 'tanatril' |
| #4 | #2 OR #3 |
| #5 | 'angiotensin receptor antagonists':ab,kw,ti OR 'antagonist, angiotensin receptor':ab,kw,ti OR 'receptor antagonist, angiotensin':ab,kw,ti OR 'angiotensin receptor blocker':ab,kw,ti OR 'receptor blocker, angiotensin':ab,kw,ti OR 'blocker, angiotensin receptor':ab,kw,ti OR 'angiotensin receptor antagonist':ab,kw,ti OR 'angiotensin ii receptor antagonist':ab,kw,ti OR 'angiotensin ii receptor blocker':ab,kw,ti |
| #6 | 'azilsartan':ab,kw,ti OR 'candesartan':ab,kw,ti OR 'eprosartan':ab,kw,ti OR 'teveten':ab,kw,ti OR 'irbesartan':ab,kw,ti OR 'avapro':ab,kw,ti OR 'karvea':ab,kw,ti OR 'aprovel':ab,kw,ti OR 'cozaar':ab,kw,ti OR 'losartan potassium':ab,kw,ti OR 'potassium':ab,kw,ti OR 'losartan monopotassium salt':ab,kw,ti OR 'monopotassium salt':ab,kw,ti OR 'losartan':ab,kw,ti OR 'salt, losartan monopotassium':ab,kw,ti OR 'olmesartan':ab,kw,ti OR 'omesartan':ab,kw,ti OR 'tasosartan':ab,kw,ti OR 'telmisartan':ab,kw,ti OR 'pritor':ab,kw,ti OR 'micardis':ab,kw,ti OR 'valsartan':ab,kw,ti OR 'diovan':ab,kw,ti OR 'tareg':ab,kw,ti OR 'nisis':ab,kw,ti OR 'provas':ab,kw,ti OR 'vals':ab,kw,ti OR 'saprisartan potassium':ab,kw,ti OR 'pratosartan':ab,kw,ti OR 'milfasartan':ab,kw,ti OR 'fimasartan':ab,kw,ti |
| #7 | 'mineralocorticoid receptor antagonists':ab,kw,ti OR 'antagonist, mineralocorticoid receptor':ab,kw,ti OR 'receptor antagonist, mineralocorticoid':ab,kw,ti OR 'mineralocorticoid antagonist':ab,kw,ti OR 'antagonist, mineralocorticoid':ab,kw,ti OR 'aldosterone receptor antagonist':ab,kw,ti OR 'antagonist, aldosterone receptor':ab,kw,ti OR 'receptor antagonist, aldosterone':ab,kw,ti OR 'mineralocorticoid receptor antagonist':ab,kw,ti OR 'aldosterone antagonist':ab,kw,ti OR 'antagonist, aldosterone':ab,kw,ti |
| #8 | 'finerenone':ab,kw,ti OR 'kerendia':ab,kw,ti OR 'bay 94-8862':ab,kw,ti OR 'esaxerenone':ab,kw,ti OR 'cs-3150':ab,kw,ti OR 'apararenone':ab,kw,ti OR 'mt-3995':ab,kw,ti OR 'eplerenone':ab,kw,ti OR 'eplerenon':ab,kw,ti OR 'inspra':ab,kw,ti OR 'spironolactone':ab,kw,ti OR 'spirolactone':ab,kw,ti OR 'veroshpiron':ab,kw,ti OR 'verospirone':ab,kw,ti OR 'spiractin':ab,kw,ti OR 'spirogamma':ab,kw,ti OR 'spirolang':ab,kw,ti OR 'aldactone':ab,kw,ti OR 'verospiron':ab,kw,ti OR 'aldactone a':ab,kw,ti |
| #9 | 'sodium-glucose transporter 2 inhibitors':ab,kw,ti OR 'sodium glucose transporter 2 inhibitors':ab,kw,ti OR 'sglt-2 inhibitors':ab,kw,ti OR 'sglt 2 inhibitors':ab,kw,ti OR 'sglt2 inhibitors':ab,kw,ti OR 'sodium-glucose transporter 2 inhibitor':ab,kw,ti OR 'sodium glucose transporter 2 inhibitor':ab,kw,ti OR 'sglt2 inhibitor':ab,kw,ti OR 'inhibitor, sglt2':ab,kw,ti OR 'sodium?glucose?transporter?ii?inhibitor':ab,kw,ti OR 'gliflozins':ab,kw,ti OR 'gliflozin':ab,kw,ti OR 'sglt-2 inhibitor':ab,kw,ti OR 'inhibitor, sglt-2':ab,kw,ti OR 'sglt 2 inhibitor':ab,kw,ti |
| #10 | 'dapagliflozin':ab,kw,ti OR 'farxiga':ab,kw,ti OR 'forxiga':ab,kw,ti OR 'canagliflozin':ab,kw,ti OR 'invokana':ab,kw,ti OR 'canagliflozin hemihydrate':ab,kw,ti OR 'canagliflozin, anhydrous':ab,kw,ti OR 'empagliflozin':ab,kw,ti OR 'jardiance':ab,kw,ti OR 'ipragliflozin':ab,kw,ti OR 'suglat':ab,kw,ti OR 'luseogliflozin':ab,kw,ti OR 'lusefi':ab,kw,ti OR 'remogliflozin':ab,kw,ti OR 'topogliflozin':ab,kw,ti OR 'sergliflozin':ab,kw,ti OR 'ertugliflozin':ab,kw,ti OR 'tofogliflozin':ab,kw,ti OR 'bexagliflozin':ab,kw,ti OR 'henagliflozin':ab,kw,ti OR 'licogliflozin':ab,kw,ti OR 'sotagliflozin':ab,kw,ti |
| #11 | #1 OR #4 OR #5 OR #6 OR #7 OR #8 OR #9 OR #10 |
| #12 | 'diabetic nephropathies':ab,kw,ti OR 'nephropathies, diabetic':ab,kw,ti OR 'nephropathy, diabetic':ab,kw,ti OR 'diabetic nephropathy':ab,kw,ti OR 'diabetic kidney disease':ab,kw,ti OR 'diabetic kidney diseases':ab,kw,ti OR 'kidney disease, diabetic':ab,kw,ti OR 'kidney diseases, diabetic':ab,kw,ti OR 'diabetic glomerulosclerosis':ab,kw,ti OR 'glomerulosclerosis, diabetic':ab,kw,ti |
| #13 | 'diabetes mellitus':ab,kw,ti OR 'diabetes':ab,kw,ti OR 'diabetic':ab,kw,ti |
| #14 | 'albuminuria':ab,kw,ti OR 'proteinuria':ab,kw,ti |
| #15 | #13 AND #14 |
| #16 | #12 OR #15 |
| #17 | 'controlled clinical trial':ab,kw,ti OR 'randomized controlled trial':ab,kw,ti OR 'random':ab,kw,ti OR 'randomly':ab,kw,ti OR 'randomized':ab,kw,ti OR 'randomised':ab,kw,ti OR 'trial':ab,kw,ti OR 'clinical trial':ab,kw,ti OR 'clinical trials':ab,kw,ti OR 'rct':ab,kw,ti OR 'rcts':ab,kw,ti OR 'placebo':ab,kw,ti |
| #18 | #11 AND #16 AND #17 |

| **Web of science** | |
| --- | --- |
| #1 | (((((((((TS=(Renin inhibitors)) OR TS=(Renin)) OR TS=(aliskiren)) OR TS=(rasilez)) OR TS=(Tekturna)) OR TS=(ciprokiren)) OR TS=(ditekiren)) OR TS=(enalkiren)) OR TS=(remikiren)) OR TS=(terlakiren) |
| #2 | (((((((((((((((((((((TS=(Angiotensin-Converting Enzyme Inhibitors)) OR TS=(Angiotensin Converting Enzyme Inhibitors)) OR TS=(Enzyme Inhibitors, Angiotensin-Converting)) OR TS=(Inhibitor, Angiotensin-Converting  Enzyme)) OR TS=(Inhibitor, Angiotensin Converting Enzyme)) OR TS=(Inhibitor, Kininase II)) OR TS=(Kininase II Antagonists)) OR TS=(Kininase II Inhibitor)) OR TS=(Angiotensin-Converting Enzyme Antagonists)) OR TS=(Angiotensin Converting Enzyme Antagonists)) OR TS=(Enzyme Antagonists, Angiotensin-Converting)) OR TS=(II Inhibitor, Kininase)) OR TS=(Antagonists, Kininase II)) OR TS=(Inhibitor, ACE)) OR TS=(ACE Inhibitor)) OR TS=(Angiotensin I-Converting Enzyme Inhibitor)) OR TS=(Angiotensin I Converting Enzyme Inhibitor)) OR TS=(Angiotensin Converting Enzyme Inhibitor)) OR TS=(Angiotensin-Converting Enzyme Inhibitor)) OR TS=(Enzyme Inhibitor, Angiotensin-Converting)) OR TS=(Antagonists, Angiotensin-Converting Enzyme)) OR TS=(Antagonists, Angiotensin Converting Enzyme) |
| #3 | ((((((((((((((((((((((((((((((((((((((((((((((((((((((((((((((((((((((((((((((((((((((((((((((((((((((((TS=(Angiotensin-Converting Enzyme Inhibitors)) OR TS=(Angiotensin Converting Enzyme Inhibitors)) OR TS=(Enzyme Inhibitors, Angiotensin-Converting)) OR TS=(Inhibitor, Angiotensin-Converting  Enzyme)) OR TS=(Inhibitor, Angiotensin Converting Enzyme)) OR TS=(Inhibitor, Kininase II)) OR TS=(Kininase II Antagonists)) OR TS=(Kininase II Inhibitor)) OR TS=(Angiotensin-Converting Enzyme Antagonists)) OR TS=(Angiotensin Converting Enzyme Antagonists)) OR TS=(Enzyme Antagonists, Angiotensin-Converting)) OR TS=(II Inhibitor, Kininase)) OR TS=(Antagonists, Kininase II)) OR TS=(Inhibitor, ACE)) OR TS=(ACE Inhibitor)) OR TS=(Angiotensin I-Converting Enzyme Inhibitor)) OR TS=(Angiotensin I Converting Enzyme Inhibitor)) OR TS=(Angiotensin Converting Enzyme Inhibitor)) OR TS=(Angiotensin-Converting Enzyme Inhibitor)) OR TS=(Enzyme Inhibitor, Angiotensin-Converting)) OR TS=(Antagonists, Angiotensin-Converting Enzyme)) OR TS=(Antagonists, Angiotensin Converting Enzyme)) OR TS=(Captopril)) OR TS=(Capoten)) OR TS=(Enalapril)) OR TS=(Renitec)) OR TS=(Renitek)) OR TS=(Enalapril Maleate)) OR TS=(Lisinopril)) OR TS=(Lysinopril)) OR TS=(Prinivil)) OR TS=(Zestril)) OR TS=(Perindopril)) OR TS=(Pirindopril)) OR TS=(Perindopril Erbumine)) OR TS=(Perstarium)) OR TS=(Quinapril)) OR TS=(Accupril)) OR TS=(Quinapril Hydrochloride)) OR TS=(Ramipril)) OR TS=(Vesdil)) OR TS=(Triatec)) OR TS=(Altace)) OR TS=(Ramace)) OR TS=(Tritace)) OR TS=(Acovil)) OR TS=(Delix)) OR TS=(Carasel)) OR TS=(Delapril)) OR TS=(Derapril)) OR TS=(Benazepril)) OR TS=(benzazepril)) OR TS=(Labopal)) OR TS=(Lotensin)) OR TS=(Cibacen)) OR TS=(Briem)) OR TS=(benazepril hydrochloride)) OR TS=(cilazapril)) OR TS=(Cilazapril Monohydrobromide)) OR TS=(Cilazapril, Anhydrous)) OR TS=(Cilazapril Anhydrous)) OR TS=(Cilazapril Monohydrate)) OR TS=(Cilazapril Hydrate)) OR TS=(Inhibace)) OR TS=(Fosinopril)) OR TS=(Fosenopril)) OR TS=(Fosinil)) OR TS=(Fosinopril Sodium)) OR TS=(Tenso Stop)) OR TS=(Monopril)) OR TS=(Fosinorm)) OR TS=(Newace)) OR TS=(Staril)) OR TS=(Moexipril)) OR TS=(Fempress)) OR TS=(moexipril hydrochloride)) OR TS=(Univasc)) OR TS=(Moex)) OR TS=(Perdix)) OR TS=(Spirapril)) OR TS=(spirapril hydrochloride)) OR TS=(Renpress)) OR TS=(Quadropril)) OR TS=(Pentopril)) OR TS=(Trandolapril)) OR TS=(Odrik)) OR TS=(Mavik)) OR TS=(Gopten)) OR TS=(Temocapril)) OR TS=(Imidapril)) OR TS=(imidapril hydrochloride)) OR TS=(alacepril)) OR TS=(aracepril)) OR TS=(altiopril)) OR TS=(ceranapril)) OR TS=(idrapril)) OR TS=(indolapril)) OR TS=( Indolapril hydrochloride)) OR TS=(Libenzapril)) OR TS=(Rentiapril)) OR TS=(Utibapril)) OR TS=(Zabicipril)) OR TS=(Zofenopril)) OR TS=( Zofenil)) OR TS=(Tanatril) |
| #4 | (((((((((((((((((((((((((((((((((((((((((TS=(Angiotensin Receptor Antagonists)) OR TS=(Antagonist, Angiotensin Receptor)) OR TS=(Receptor Antagonist, Angiotensin)) OR TS=(Angiotensin Receptor Blocker)) OR TS=(Receptor Blocker, Angiotensin)) OR TS=(Blocker, Angiotensin Receptor)) OR TS=(Angiotensin Receptor Antagonist)) OR TS=(Angiotensin II Receptor Antagonist)) OR TS=(Angiotensin II Receptor Blocker)) OR TS=(Azilsartan)) OR TS=(Candesartan)) OR TS=(Eprosartan)) OR TS=(Teveten)) OR TS=(Irbesartan)) OR TS=(Avapro)) OR TS=(Karvea)) OR TS=(Aprovel)) OR TS=(Losartan)) OR TS=(Cozaar)) OR TS=(Losartan Potassium)) OR TS=(Potassium)) OR TS=(Losartan)) OR TS=(Losartan Monopotassium Salt)) OR TS=(Monopotassium Salt)) OR TS=( Losartan)) OR TS=(Salt, Losartan Monopotassium)) OR TS=(Olmesartan)) OR TS=(omesartan)) OR TS=(tasosartan)) OR TS=(Telmisartan)) OR TS=(Pritor)) OR TS=(Micardis)) OR TS=(Valsartan)) OR TS=(Diovan)) OR TS=(Tareg)) OR TS=(Nisis)) OR TS=(Provas)) OR TS=(Vals)) OR TS=(saprisartan potassium)) OR TS=(pratosartan)) OR TS=(milfasartan)) OR TS=(fimasartan) |
| #5 | ((((((((((((((((((((((((((((((TS=(Mineralocorticoid Receptor Antagonists)) OR TS=(Antagonist, Mineralocorticoid Receptor)) OR TS=(Receptor Antagonist, Mineralocorticoid)) OR TS=(Mineralocorticoid Antagonist)) OR TS=(Antagonist, Mineralocorticoid)) OR TS=(Aldosterone Receptor Antagonist)) OR TS=(Antagonist, Aldosterone Receptor)) OR TS=(Receptor Antagonist, Aldosterone)) OR TS=(Mineralocorticoid Receptor Antagonist)) OR TS=(Aldosterone Antagonist)) OR TS=(Antagonist, Aldosterone)) OR TS=(Finerenone)) OR TS=(kerendia)) OR TS=(BAY 94-8862)) OR TS=(esaxerenone)) OR TS=(CS-3150)) OR TS=(Apararenone )) OR TS=(MT-3995)) OR TS=(Eplerenone)) OR TS=(Eplerenon)) OR TS=(Inspra)) OR TS=(Spironolactone)) OR TS=(Spirolactone)) OR TS=(Veroshpiron)) OR TS=(Verospirone)) OR TS=(Spiractin)) OR TS=(Spirogamma)) OR TS=(Spirolang)) OR TS=(Aldactone)) OR TS=(Verospiron)) OR TS=(Aldactone A) |
| #6 | ((((((((((((((((((((((((((((((((((((TS=(Sodium-Glucose Transporter 2 Inhibitors)) OR TS=(Sodium Glucose Transporter 2 Inhibitors)) OR TS=(SGLT-2 Inhibitors)) OR TS=(SGLT 2 Inhibitors)) OR TS=(SGLT2 Inhibitors)) OR TS=(Sodium-Glucose Transporter 2 Inhibitor)) OR TS=(Sodium Glucose Transporter 2 Inhibitor)) OR TS=(SGLT2 Inhibitor)) OR TS=(Inhibitor, SGLT2)) OR TS=(sodium glucose transporter ii inhibitor)) OR TS=(Gliflozins)) OR TS=(Gliflozin)) OR TS=(SGLT-2 Inhibitor)) OR TS=(Inhibitor, SGLT-2)) OR TS=(SGLT 2 Inhibitor )) OR TS=(Dapagliflozin)) OR TS=(Farxiga)) OR TS=(Forxiga)) OR TS=(Canagliflozin)) OR TS=(Invokana)) OR TS=(Canagliflozin Hemihydrate)) OR TS=(Canagliflozin, Anhydrous)) OR TS=(Empagliflozin)) OR TS=(Jardiance)) OR TS=(Ipragliflozin)) OR TS=(Suglat)) OR TS=(luseogliflozin)) OR TS=(Lusefi)) OR TS=(remogliflozin)) OR TS=(Topogliflozin)) OR TS=(Sergliflozin)) OR TS=(ertugliflozin)) OR TS=(tofogliflozin)) OR TS=(Bexagliflozin)) OR TS=(henagliflozin)) OR TS=(licogliflozin)) OR TS=(sotagliflozin) |
| #7 | #6 OR #5 OR #4 OR #3 OR #2 OR #1 |
| #8 | (((((((((TS=(Diabetic Nephropathies)) OR TS=(Nephropathies, Diabetic)) OR TS=(Nephropathy, Diabetic)) OR TS=(Diabetic Nephropathy)) OR TS=(Diabetic Kidney Disease)) OR TS=(Diabetic Kidney Diseases)) OR TS=(Kidney Disease, Diabetic)) OR TS=(Kidney Diseases, Diabetic)) OR TS=(Diabetic Glomerulosclerosis)) OR TS=(Glomerulosclerosis, Diabetic) |
| #9 | ((TS=(Diabetes Mellitus)) OR TS=(Diabetes)) OR TS=(Diabetic) |
| #10 | (TS=(Albuminuria)) OR TS=( Proteinuria) |
| #11 | #10 AND #9 |
| #12 | #11 OR #8 |
| #13 | ((((((((((((TS=(randomized controlled trial )) OR TS=(controlled clinical trial )) OR TS=(randomized controlled trial)) OR TS=(random)) OR TS=(randomly)) OR TS=(randomized )) OR TS=(randomised)) OR TS=(trial )) OR TS=(clinical trial)) OR TS=(Clinical Trials as Topic)) OR TS=(RCT)) OR TS=(RCTs)) OR TS=(Placebo) |
| #14 | #13 AND #12 AND #7 |

| **Scopus** | |
| --- | --- |
| #1 | TITLE-ABS-KEY ( randomized AND controlled AND trial ) OR TITLE-ABS-KEY ( controlled AND clinical AND trial ) OR TITLE-ABS-KEY ( randomized AND controlled AND trial ) OR TITLE-ABS-KEY ( random ) OR TITLE-ABS-KEY ( randomly ) OR TITLE-ABS-KEY ( randomized ) OR TITLE-ABS-KEY ( randomised ) OR TITLE-ABS-KEY ( trial ) OR TITLE-ABS-KEY ( clinical AND trial ) OR TITLE-ABS-KEY ( clinical AND trials ) OR TITLE-ABS-KEY ( rct ) OR TITLE-ABS-KEY ( rcts ) OR TITLE-ABS-KEY ( placebo ) |
| #2 | TITLE-ABS-KEY ( albuminuria ) OR TITLE-ABS-KEY ( proteinuria ) |
| #3 | TITLE-ABS-KEY ( diabetes AND mellitus ) OR TITLE-ABS-KEY ( diabetes ) OR TITLE-ABS-KEY ( diabetic ) |
| #4 | #2 AND #3 |
| #5 | TITLE-ABS-KEY ( diabetic AND nephropathies ) OR TITLE-ABS-KEY ( nephropathies, AND diabetic ) OR TITLE-ABS-KEY ( nephropathy, AND diabetic ) OR TITLE-ABS-KEY ( diabetic AND nephropathy ) OR TITLE-ABS-KEY ( diabetic AND kidney AND disease ) OR TITLE-ABS-KEY ( diabetic AND kidney AND diseases ) OR TITLE-ABS-KEY ( kidney AND disease, AND diabetic ) OR TITLE-ABS-KEY ( kidney AND diseases, AND diabetic ) OR TITLE-ABS-KEY ( diabetic AND glomerulosclerosis ) OR TITLE-ABS-KEY ( glomerulosclerosis, AND diabetic ) |
| #6 | #4 OR #5 |
| #7 | TITLE-ABS-KEY ( renin AND inhibitors ) OR TITLE-ABS-KEY ( renin ) OR TITLE-ABS-KEY ( aliskiren ) OR TITLE-ABS-KEY ( rasilez ) OR TITLE-ABS-KEY ( tekturna ) OR TITLE-ABS-KEY ( ciprokiren ) OR TITLE-ABS-KEY ( ditekiren ) OR TITLE-ABS-KEY ( enalkiren ) OR TITLE-ABS-KEY ( remikiren ) OR TITLE-ABS-KEY ( terlakiren ) |
| #8 | TITLE-ABS-KEY ( angiotensin-converting AND enzyme AND inhibitors ) OR TITLE-ABS-KEY ( angiotensin AND converting AND enzyme AND inhibitors ) OR TITLE-ABS-KEY ( enzyme AND inhibitors, AND angiotensin-converting ) OR TITLE-ABS-KEY ( inhibitor, AND angiotensin-converting AND enzyme ) OR TITLE-ABS-KEY ( inhibitor, AND angiotensin AND converting AND enzyme ) OR TITLE-ABS-KEY ( inhibitor, AND kininase AND ii ) OR TITLE-ABS-KEY ( kininase AND ii AND antagonists ) OR TITLE-ABS-KEY ( kininase AND ii AND inhibitor ) OR TITLE-ABS-KEY ( angiotensin-converting AND enzyme AND antagonists ) OR TITLE-ABS-KEY ( angiotensin AND converting AND enzyme AND antagonists ) OR TITLE-ABS-KEY ( enzyme AND antagonists, AND angiotensin-converting ) OR TITLE-ABS-KEY ( ii AND inhibitor, AND kininase ) OR TITLE-ABS-KEY ( antagonists, AND kininase AND ii ) OR TITLE-ABS-KEY ( inhibitor, AND ace ) OR TITLE-ABS-KEY ( ace AND inhibitor ) OR TITLE-ABS-KEY ( angiotensin AND i-converting AND enzyme AND inhibitor ) OR TITLE-ABS-KEY ( angiotensin AND i AND converting AND enzyme AND inhibitor ) OR TITLE-ABS-KEY ( angiotensin AND converting AND enzyme AND inhibitor ) OR TITLE-ABS-KEY ( angiotensin-converting AND enzyme AND inhibitor ) OR TITLE-ABS-KEY ( enzyme AND inhibitor, AND angiotensin-converting ) OR TITLE-ABS-KEY ( antagonists, AND angiotensin-converting AND enzyme ) OR TITLE-ABS-KEY ( antagonists, AND angiotensin AND converting AND enzyme ) OR TITLE-ABS-KEY ( captopril ) OR TITLE-ABS-KEY ( capoten ) OR TITLE-ABS-KEY ( enalapril ) OR TITLE-ABS-KEY ( renitec ) OR TITLE-ABS-KEY ( renitek ) OR TITLE-ABS-KEY ( enalapril AND maleate ) OR TITLE-ABS-KEY ( lisinopril ) OR TITLE-ABS-KEY ( lysinopril ) OR TITLE-ABS-KEY ( prinivil ) OR TITLE-ABS-KEY ( zestril ) OR TITLE-ABS-KEY ( perindopril ) OR TITLE-ABS-KEY ( pirindopril ) OR TITLE-ABS-KEY ( perindopril AND erbumine ) OR TITLE-ABS-KEY ( perstarium ) OR TITLE-ABS-KEY ( quinapril ) OR TITLE-ABS-KEY ( accupril ) OR TITLE-ABS-KEY ( quinapril AND hydrochloride ) OR TITLE-ABS-KEY ( ramipril ) OR TITLE-ABS-KEY ( vesdil ) OR TITLE-ABS-KEY ( triatec ) OR TITLE-ABS-KEY ( altace ) OR TITLE-ABS-KEY ( ramace ) OR TITLE-ABS-KEY ( tritace ) OR TITLE-ABS-KEY ( acovil ) OR TITLE-ABS-KEY ( delix ) OR TITLE-ABS-KEY ( carasel ) OR TITLE-ABS-KEY ( delapril ) OR TITLE-ABS-KEY ( derapril ) OR TITLE-ABS-KEY ( benazepril ) OR TITLE-ABS-KEY ( benzazepril ) OR TITLE-ABS-KEY ( labopal ) OR TITLE-ABS-KEY ( lotensin ) OR TITLE-ABS-KEY ( cibacen ) OR TITLE-ABS-KEY ( briem ) OR TITLE-ABS-KEY ( benazepril AND hydrochloride ) OR TITLE-ABS-KEY ( cilazapril ) OR TITLE-ABS-KEY ( cilazapril AND monohydrobromide ) OR TITLE-ABS-KEY ( cilazapril ) OR TITLE-ABS-KEY ( anhydrous ) OR TITLE-ABS-KEY ( cilazapril AND anhydrous ) OR TITLE-ABS-KEY ( cilazapril AND monohydrate ) OR TITLE-ABS-KEY ( cilazapril AND hydrate ) OR TITLE-ABS-KEY ( inhibace ) OR TITLE-ABS-KEY ( fosinopril ) OR TITLE-ABS-KEY ( fosenopril ) OR TITLE-ABS-KEY ( fosinil ) OR TITLE-ABS-KEY ( fosinopril AND sodium ) OR TITLE-ABS-KEY ( tenso AND stop ) OR TITLE-ABS-KEY ( monopril ) OR TITLE-ABS-KEY ( fosinorm ) OR TITLE-ABS-KEY ( newace ) OR TITLE-ABS-KEY ( staril ) OR TITLE-ABS-KEY ( moexipril ) OR TITLE-ABS-KEY ( fempress ) OR TITLE-ABS-KEY ( moexipril AND hydrochloride ) OR TITLE-ABS-KEY ( univasc ) OR TITLE-ABS-KEY ( moex ) OR TITLE-ABS-KEY ( perdix ) OR TITLE-ABS-KEY ( spirapril ) OR TITLE-ABS-KEY ( spirapril AND hydrochloride ) OR TITLE-ABS-KEY ( renpress ) OR TITLE-ABS-KEY ( quadropril ) OR TITLE-ABS-KEY ( pentopril ) OR TITLE-ABS-KEY ( trandolapril ) OR TITLE-ABS-KEY ( odrik ) OR TITLE-ABS-KEY ( mavik ) OR TITLE-ABS-KEY ( gopten ) OR TITLE-ABS-KEY ( temocapril ) OR TITLE-ABS-KEY ( imidapril ) OR TITLE-ABS-KEY ( imidapril AND hydrochloride ) OR TITLE-ABS-KEY ( alacepril ) OR TITLE-ABS-KEY ( aracepril ) OR TITLE-ABS-KEY ( altiopril ) OR TITLE-ABS-KEY ( ceranapril ) OR TITLE-ABS-KEY ( idrapril ) OR TITLE-ABS-KEY ( indolapril ) OR TITLE-ABS-KEY ( indolapril AND hydrochloride ) OR TITLE-ABS-KEY ( libenzapril ) OR TITLE-ABS-KEY ( rentiapril ) OR TITLE-ABS-KEY ( utibapril ) OR TITLE-ABS-KEY ( zabicipril ) OR TITLE-ABS-KEY ( zofenopril ) OR TITLE-ABS-KEY ( zofenil ) OR TITLE-ABS-KEY ( tanatril ) |
| #9 | TITLE-ABS-KEY ( angiotensin AND receptor AND antagonists ) OR TITLE-ABS-KEY ( antagonist, AND angiotensin AND receptor ) OR TITLE-ABS-KEY ( receptor AND antagonist, AND angiotensin ) OR TITLE-ABS-KEY ( angiotensin AND receptor AND blocker ) OR TITLE-ABS-KEY ( receptor AND blocker, AND angiotensin ) OR TITLE-ABS-KEY ( blocker, AND angiotensin AND receptor ) OR TITLE-ABS-KEY ( angiotensin AND receptor AND antagonist ) OR TITLE-ABS-KEY ( angiotensin AND ii AND receptor AND antagonist ) OR TITLE-ABS-KEY ( angiotensin AND ii AND receptor AND blocker ) OR TITLE-ABS-KEY ( azilsartan ) OR TITLE-ABS-KEY ( candesartan ) OR TITLE-ABS-KEY ( eprosartan ) OR TITLE-ABS-KEY ( teveten ) OR TITLE-ABS-KEY ( irbesartan ) OR TITLE-ABS-KEY ( avapro ) OR TITLE-ABS-KEY ( karvea ) OR TITLE-ABS-KEY ( aprovel ) OR TITLE-ABS-KEY ( losartan ) OR TITLE-ABS-KEY ( cozaar ) OR TITLE-ABS-KEY ( losartan AND potassium ) OR TITLE-ABS-KEY ( potassium ) OR TITLE-ABS-KEY ( losartan ) OR TITLE-ABS-KEY ( losartan AND monopotassium AND salt ) OR TITLE-ABS-KEY ( monopotassium AND salt ) OR TITLE-ABS-KEY ( losartan ) OR TITLE-ABS-KEY ( salt, AND losartan AND monopotassium ) OR TITLE-ABS-KEY ( olmesartan ) OR TITLE-ABS-KEY ( omesartan ) OR TITLE-ABS-KEY ( tasosartan ) OR TITLE-ABS-KEY ( telmisartan ) OR TITLE-ABS-KEY ( pritor ) OR TITLE-ABS-KEY ( micardis ) OR TITLE-ABS-KEY ( valsartan ) OR TITLE-ABS-KEY ( diovan ) OR TITLE-ABS-KEY ( tareg ) OR TITLE-ABS-KEY ( nisis ) OR TITLE-ABS-KEY ( provas ) OR TITLE-ABS-KEY ( vals ) OR TITLE-ABS-KEY ( saprisartan AND potassium ) OR TITLE-ABS-KEY ( pratosartan ) OR TITLE-ABS-KEY ( milfasartan ) OR TITLE-ABS-KEY ( fimasartan ) |
| #10 | TITLE-ABS-KEY ( mineralocorticoid AND receptor AND antagonists ) OR TITLE-ABS-KEY ( antagonist, AND mineralocorticoid AND receptor ) OR TITLE-ABS-KEY ( receptor AND antagonist, AND mineralocorticoid ) OR TITLE-ABS-KEY ( mineralocorticoid AND antagonist ) OR TITLE-ABS-KEY ( antagonist, AND mineralocorticoid ) OR TITLE-ABS-KEY ( aldosterone AND receptor AND antagonist ) OR TITLE-ABS-KEY ( antagonist, AND aldosterone AND receptor ) OR TITLE-ABS-KEY ( receptor AND antagonist, AND aldosterone ) OR TITLE-ABS-KEY ( mineralocorticoid AND receptor AND antagonist ) OR TITLE-ABS-KEY ( aldosterone AND antagonist ) OR TITLE-ABS-KEY ( antagonist, AND aldosterone ) OR TITLE-ABS-KEY ( finerenone ) OR TITLE-ABS-KEY ( kerendia ) OR TITLE-ABS-KEY ( bay 94-8862 ) OR TITLE-ABS-KEY ( esaxerenone ) OR TITLE-ABS-KEY ( cs-3150 ) OR TITLE-ABS-KEY ( apararenone ) OR TITLE-ABS-KEY ( mt-3995 ) OR TITLE-ABS-KEY ( eplerenone ) OR TITLE-ABS-KEY ( eplerenon ) OR TITLE-ABS-KEY ( inspra ) OR TITLE-ABS-KEY ( spironolactone ) OR TITLE-ABS-KEY ( spirolactone ) OR TITLE-ABS-KEY ( veroshpiron ) OR TITLE-ABS-KEY ( verospirone ) OR TITLE-ABS-KEY ( spiractin ) OR TITLE-ABS-KEY ( spirogamma ) OR TITLE-ABS-KEY ( spirolang ) OR TITLE-ABS-KEY ( aldactone ) OR TITLE-ABS-KEY ( verospiron ) OR TITLE-ABS-KEY ( aldactone AND a ) |
| #11 | TITLE-ABS-KEY ( sodium-glucose AND transporter 2 inhibitors ) OR TITLE-ABS-KEY ( sodium AND glucose AND transporter 2 inhibitors ) OR TITLE-ABS-KEY ( sglt-2 AND inhibitors ) OR TITLE-ABS-KEY ( sglt 2 inhibitors ) OR TITLE-ABS-KEY ( sglt2 AND inhibitors ) OR TITLE-ABS-KEY ( sodium-glucose AND transporter 2 inhibitor ) OR TITLE-ABS-KEY ( sodium AND glucose AND transporter 2 inhibitor ) OR TITLE-ABS-KEY ( sglt2 AND inhibitor ) OR TITLE-ABS-KEY ( inhibitor, AND sglt2 ) OR TITLE-ABS-KEY ( sodium AND glucose AND transporter AND ii AND inhibitor ) OR TITLE-ABS-KEY ( gliflozins ) OR TITLE-ABS-KEY ( gliflozin ) OR TITLE-ABS-KEY ( sglt-2 AND inhibitor ) OR TITLE-ABS-KEY ( inhibitor, AND sglt-2 ) OR TITLE-ABS-KEY ( sglt 2 inhibitor ) OR TITLE-ABS-KEY ( dapagliflozin ) OR TITLE-ABS-KEY ( farxiga ) OR TITLE-ABS-KEY ( forxiga ) OR TITLE-ABS-KEY ( canagliflozin ) OR TITLE-ABS-KEY ( invokana ) OR TITLE-ABS-KEY ( canagliflozin AND hemihydrate ) OR TITLE-ABS-KEY ( canagliflozin, AND anhydrous ) OR TITLE-ABS-KEY ( empagliflozin ) OR TITLE-ABS-KEY ( jardiance&#65292;ipragliflozin ) OR TITLE-ABS-KEY ( suglat ) OR TITLE-ABS-KEY ( luseogliflozin ) OR TITLE-ABS-KEY ( lusefi ) OR TITLE-ABS-KEY ( remogliflozin ) OR TITLE-ABS-KEY ( topogliflozin ) OR TITLE-ABS-KEY ( sergliflozin ) OR TITLE-ABS-KEY ( ertugliflozin ) OR TITLE-ABS-KEY ( tofogliflozin ) OR TITLE-ABS-KEY ( bexagliflozin ) OR TITLE-ABS-KEY ( henagliflozin ) OR TITLE-ABS-KEY ( licogliflozin ) OR TITLE-ABS-KEY ( sotagliflozin ) |
| #12 | #7 OR #8 OR #9 OR #10 OR #11 |
| #13 | #1 AND #6 AND #12 |

**Supplementary Table S2: selection criteria**

| **Inclusion criteria** | **Exclusion criteria** |
| --- | --- |
| Randomized controlled trial, post hoc analysis or pooled study | Randomized cross-over clinical trial; Single-arm trial |
| Patients diagnosed with diabetic kidney disease | No diagnosis of diabetic kidney disease; History of nephrectomy, renal transplant, or dialysis treatment; People unable to give informed consent and pregnant, potentially pregnant women; uncontrolled hypertension |
| Comparison between SGLT2i, ACEI, ARB, RI, MRA or placebo administered either as monotherapy or in combination. | Intervention compared drugs of the same category |
| Primary outcome: hyperkalemia^*^  Secondary outcome: serum potassium | Zero event trials; No report of hyperkalemia and serum potassium |
| Minimum follow-up 12 weeks | Full text unavailable |
| Participants aged ≥18 years | Duplicate publications |
| Publication in English |  |

^*^: The definition of hyperkalemia adhered to the standard definition that was previously employed in the original studies. SGLT-2i: sodium-glucose cotransporter-2 inhibitors, ACEI: angiotensin-converting enzyme inhibitors, ARB: angiotensin receptor blockers, MRA: mineralocorticoid receptor antagonists, RI: renin inhibitors.

**Supplementary Table S3:** **Summary of characteristics of included studies**

| Site | Study (Author year) | Register number | Length of follow-up (Weeks) | Definition of hyperkalemia (Serum potassium, mmol/L) | Diabetes type | Stage of CKD |
| --- | --- | --- | --- | --- | --- | --- |
| Egypt | Mokadem 2020^[1]^ | NCT04143412 | 24 weeks | >5.5 | 2 | Microalbuminuria between 30 and 300 mg/g |
| USA | Bauer 1992^[2]^ | NO | 72 weeks | >5.7 | 1/2 | Persistent proteinuria greater than 500 mg/24 h; creatinine clearance greater than 20 mL/ min/1.73 m2 |
| USA | Lewis 1993^[3]^ | NO | 144 weeks | >6 | 1 | Diabetic nephropathy, urinary protein excretion of ≥ 500 mg/24 h and serum creatinine ≤ 2.5mg/dl |
| 210 clinical centers | Lewis 2001^[4]^ | NO | 124.8 weeks (2.6 years) | NO | 2 | Urinary protein excretion of at least 900 mg/24 h, the serum creatinine concentration was between 1.0 and 3.0 mg/dl (88 and 265 μmol/L in women and 1.2 and 3.0 mg/dl (106 and 265 μmol/L) in men |
| Netherlands | Meiracker 2006^[5]^ | NO | 48 weeks | >5.5 | 2 | Macroalbuminuria (24-h urinary albumin excretion >300 mg or UACR >20 mg/mmol) |
| China | Tong 2006^[6]^ | NO | 96 weeks | >5.5 | 2 | Mean plasma creatinine concentration between 130 and 300 mmol/l |
| 43 sites | Epstein 2006^[7]^ | NO | 12 weeks | >5.5 on two consecutive occasions 1 to 3 day apart | 2 | Albuminuria (UACR ≥ 50 mg/g) |
| 250 centers in 28 countries in Asia, Europe, Central America, South America, and North America | Winkelmayer 2006^[8]^ | NO | 163.2 weeks (3.4 years) | NO | 2 | Proteinuria (UACR≥300 mg/g) or 24-hour urine protein >500 mg, serum creatinine ≥1.5 to 3.0 mg/dL (≥1.3 mg/dL for female) |
| 15 countries and 150 centers | Parving 2008^[9]^ | NCT00097955 | 24 weeks | NO | 2 | UACR >300 mg /g, or >200 mg/g |
| USA | Mehdi 2009^[10]^ | NCT00381134 | 52 weeks | >6.0 | 1/2 | Proteinuria (24-h UACR >300 mg/g) |
| Japan and Hong Kong | Imai 2009^[11]^ | NCT00141453 | 153.6 weeks (3.2 years) | NO | 2 | UACR >300 mg/g in the first morning urine sample and serum creatinine concentration of 88.40–221.00 μmol/l (1.0– 2.5 mg/dl) in women and 106.08–221.00 μmol/l (1.2–2.5 mg/ dl) in men |
| 853 centers in 36 countries | Parving 2012^[12]^ | NCT00549757 | 131.6 weeks (32.9 months) | ≥6 | 2 | Persistent macroalbuminuria (UACR ≥ 200 mg/g or 22.6 mg/mmol) in 2 out of three first morning void urine samples and an eGFR ≥ 30 mL/min/1.73m2; Persistent microalbuminuria (UACR ≥ 20 mg/g and < 200 mg/g or [UACR ≥ 2.26 mg/mmol and <22.6 mg/mmol]) in 2 out of three first morning void urine samples and an eGFR ≥ 30 and < 60 mL/min/1.73m2 |
| Iran | Esteghamati 2013^[13]^ | NCT01667614 | 72 weeks | ≥5.5 | 2 | Urinary albumin excretion (UAE) ≥30 mg/24 h in at least two out of three |
| 17 centers in Spain | Juarez 2013^[14]^ | EudraCT; study number: 2004-002470-31 | 128 weeks | >5.5 | 2 | Stage 2 or 3 chronic kidney disease, and UPCR> 300 mg/g on a morning urine spot sample on 2 separate occasions |
| 241 centers in eight countries. | The EMPA-KIDNEY Collaborative Group 2022^[15]^ | NCT03594110 | 96 weeks | NO | 1/2 | eGFR 20-45 ml/min/1.73 m2, or eGFR 45-90 ml/min/1.73 m2 with UACR> 200 |
| 32 Department of Veterans Affairs (VA) medical centers | Fried 2013^[16]^ | NCT00555217 | 105.6 weeks (2.2 years) | > 6.0 or required an emergency room visit, hospitalization, or dialysis | 2 | UACR at least 300, and eGFR 30.0-89.9 ml/min/1.73 m2 |
| 111 sites in United States, Argentina, Canada, India, Mexico, Peru, Italy, Australia, France, Spain, Denmark, Puerto Rico, and Singapore | Kohan 2013^[17]^ | NCT00663260 | 104 weeks | ≥6 | 2 | eGFR 30 to 59 ml/min/1.73 m2 |
| 148 sites in 23 countries | Bakris 2015^[18]^ | NCT1874431 | 12 weeks | ≥5.6 | 2 | Albuminuria (UACR ≥30 mg/g), and eGFR>30 mL/min/1.73 m2 |
| China | Chen 2018^[19]^ | NO | 72 weeks | >5.5 | 2 | UAER 20-199 μg/min |
| Italy | Ruggenenti 2019^[20]^ | NCT00494715 | 216 weeks (4.5 years) | ≥6 | 2 | Serum creatinine concentration between 159 and 309 μmol/L and spot morning UACR >1000 mg/g for participants not receiving RAS inhibition therapy, or > 500 mg/g for participants given ACE inhibitor or ARB therapy |
| Japan | Ito (Esaxerenone) 2019^[21]^ | NCT02345057/JapicCTI-152774 | 12 weeks | ≥6.0 or two consecutive measurements ≥5.5 | 2 | UACR 45-300 mg/g, and eGFR ≥30 ml/min/1.73 m2 |
| 34 countries | Perkovic 2019^[22]^ | NCT02065791 | 125.76 weeks (2.62 years) | >5.5 | 2 | GFR 30-90 ml/ min/1.73 m2 and albuminuria (>300 to 5000) |
| Japan | Wada 2020^[23]^ | NCT02517320 (dose–response study) | 24 weeks | NO | 2 | UACR in a casual urine sample ≥ 50 mg/gCr (≥ 5.655 mg/mmol); median UACR ≥ 50 mg/g Cr and < 300 mg/gCr (≥ 5.655 mg/ mmol and < 33.93 mg/mmol) |
| 48 countries | Rossing 2021^[24]^ | NCT02540993 | 124.8 weeks (2.6 years) | > 5.5 | 2 | Persistent, moderately elevated albuminuria (UACR 30 -300 mg/g), an eGFR of 25 -60 ml/min/1.73 m2, and a history of diabetic retinopathy or persistent, severely elevated albuminuria (UACR 300–5000 mg/g) with an eGFR of 25-75 ml/min/1.73 m2. |
| 48 countries | Pitt 2021^[25]^ | NCT02545049 | 163.2 weeks (3.4 years) | > 5.5 | 2 | Persistent albuminuria UACR 30-300 mg/g and eGFR 25 -90 mL/min/1.73 m2 or persistent albuminuria UACR 300-5000mg/g and eGFR ≥60 mL/min/1.73 m2 |
| Japan | Shikata 2022^[26]^ | JapicCTI-173695, JapicCTI-173696 | 52 weeks, 28 weeks | ≥5.5 | 2 | UACR 45-300 mg/g or UACR 300-1,000 mg/g, and eGFR ≥30 mL/min/ 1.73 m2 |
| Multicenter | Tuttle 2022^[27]^ | NCT03594110, NCT00885118, NCT00789035, NCT00558571, NCT00749190, NCT01011868, NCT01193218, NCT01210001, NCT01177813, NCT01159600, NCT01289990, NCT01131676, NCT01164501, NCT01370005, NCT01306214, NCT01649297, NCT01947855, NCT02589639 | 4-78 weeks | NO | 2 | eGFR <60 mL/min/1.73 m2 |
| Iran | Makhlough 2014^[28]^ | IRCT138806211241N2 | 12 weeks | > 5.5 | 2 | UACR 20-200 mg/gr Cr in two random measurements with a month interval |
| Tel Aviv | Ravid 1993^[29]^ | NO | 144 weeks | NO | 2 | Serum creatinine<123 /Limol/L (1.4 mg/dL); and microalbuminuria (urinary protein excretion of 30 to 300 mg/24 h) on two consecutive visits without evidence of urinary tract infection |
| 14 hospital-based diabetes centers in northeastern Italy | Trevisan 1995^[30]^ | NO | 24 weeks | NO | 2 | Persistent microalbuminuria (ie, AER had to be 20 to 200 ~g/min at screening and in at least two of three consecutive sterile urine samples collected overnight) |
| Thailand | Vongterapak 1997^[31]^ | NO | 12 weeks | NO | 2 | Microalbuminuria least 2 in 3 tests demonstrated positive response |
| Bangladesh | Hoque 2009^[32]^ | NO | 16 weeks | ≥5.5 | 2 | Proteinuria ≥ 0.5 gm/d and serum creatinine ≤3 mg/dL |
| Iran | Ziaee 2013^[33]^ | IRCT201105084849N2 | 12 weeks | NO | 2 | Microalbuminuria |
| Italy | Imbalzano 2014^[34]^ | NO | 24 weeks | NO | 2 | Urinary albumin excretion ≥30 mg/24 h but ≤300 mg/24 h |
| 87 sites in Japan | Ito (Imarikiren) 2019^[35]^ | NCT02332824 | 12 weeks | ≥5.0 | 2 | UACR of the first morning urine of 30-300 mg/g creatinine on two or more of three measurements and serum creatinine >45 ml/min/1.73 m2 |
| France | Marre 1988^[36]^ | NO | 48 weeks | NO | 1/2 | persistent microalbuminuria (30-300mg/24 h) |

| Study (Author year) | Interventions | Total, n | Age (Mean ± SD) | Sex (n, male/female) | Baseline serum potassium (mmol/L) | Hyperkalemia (n) |
| --- | --- | --- | --- | --- | --- | --- |
| Mokadem 2020 | Ramipril 10 mg | 25 | 50.4 ± 6.70 | 13/12 | 3.98 ± 0.3 | 1 |
|  | Eplerenone 50 mg | 25 | 50.4 ± 6.63 | 15/10 | 3.97 ± 0.27 | 1 |
|  | Eplerenone 50 mg + Ramipril 10 mg | 25 | 48.48 ± 6.12 | 15/10 | 3.92 ± 0.36 | 2 |
| Bauer 1992 | Enalapril 5-40 mg daily | 18 | 44.1 ± 10 | 11/7 | 4.2 ± 0.42 | 2 |
|  | Placebo | 15 | 57.0 ± 5.75 | 13/12 | 4.0 ± 0.39 | 0 |
| Lewis 1993 | Captopril 25 mg tid | 207 | 35 ± 7 | 52/155 | NO | 3 |
|  | Placebo tablets tid | 202 | 34 ± 8 | 54/148 | NO | 0 |
| Lewis 2001 | Irbesartan 300 mg daily | 579 | 59.3 ± 7.1 | 378/201 | NO | 11 |
|  | Placebo | 569 | 58.3 ± 8.2 | 403/166 | NO | 2 |
| Meiracker 2006 | Spironolactone 25-50 mg qd + ACEI/ARB | 29 | 55.2 ± 10 | 16/7 | 4.1 ± 0.3 | 5 |
|  | Placebo + ACEI/ARB | 30 | 55.2 ± 11.5 | 17/12 | 4.2 ± 0.3 | 1 |
| Tong 2006 | Fosinopril 20 mg daily | 18 | 65.9±5.5 | 11/7 | 4.3 ± 0.5 | 8 |
|  | Placebo | 20 | 65.7±6.5 | 14/6 | 4.4 ± 0.6 | 11 |
| Epstein 2006 | Eplerenone 50-100 mg +  Enalapril 20 mg qd | 177 | 58 ± 10.4 | 116/61 | NO | 12 |
|  | Placebo + Enalapril 20 mg qd | 91 | 60 ± 9.6 | 50/41 | NO | 4 |
| Winkelmayer 2006 | Losartan 50 mg | 751 | 60 ± 7 | 462/289 | NO | 183 |
|  | Placebo | 762 | 60 ± 7 | 494/268 | NO | 94 |
| Parving 2008 | Aliskiren 150/300 mg + Losartan 100 mg daily | 301 | 59.8 ± 9.6 | 206 /95 | 4.5 ± 0.5 | 15 |
|  | Placebo + Losartan100 mg daily | 298 | 61.8 ± 9.6 | 221/77 | 4.5 ± 0.5 | 17 |
| Mehdi 2009 | Lisinopril 80 mg + Losartan 100 mg qd | 26 | 52.3 ± 9.1 | 13/13 | 4.5 ± 0.4 | 10 |
|  | Lisinopril 80 mg + Spironolactone 25 mg qd | 27 | 51.7 ± 9.3 | 13/14 | 4.5 ± 0.7 | 14 |
|  | Lisinopril 80 mg + Placebo qd | 27 | 49.3 ± 8.8 | 12/15 | 4.5 ± 0.7 | 2 |
| Imai 2009 | Olmesartan 10 mg qd + ACEI | 282 | 59.1 ± 8.1 | 199/83 | 4.61 ± 0.43 | 26 |
|  | Placebo + ACEI | 284 | 59.2 ± 8.1 | 192/92 | 4.61 ± 0.41 | 15 |
| Parving 2012 | Aliskiren 300 mg daily + ACEI/ARB | 4274 | 64.6 ± 9.6 | 2881/1393 | NO | 1670 |
|  | Placebo + ACEI/ARB | 4287 | 64.4 ± 9.9 | 2945/1342 | NO | 1244 |
| Esteghamati 2013 | Spironolactone 25 mg + Losartan 50–100 mg daily | 74 | 57.80 ± 8.91 | 51/23 | 4.40 ± 0.43 | 3 |
|  | Enalapril 30–40 mg + Losartan 50–100 mg daily | 62 | 58.33 ± 9.33 | 40/22 | 4.32 ± 0.44 | 0 |
| Juarez 2013 | Lisinopril 40 mg qd | 35 | 68.7 ± 6.8 | 25/10 | 4.46 ± 0.6 | 13 |
|  | Irbesartan 600 mg qd | 28 | 67.9 ± 8.0 | 21/7 | 4.42 ± 0.5 | 11 |
|  | Lisinopril 20 mg + irbesartan 300 mg qd | 70 | 63.0 ± 8.5 | 55/25 | 4.45 ± 0.5 | 23 |
| The EMPA-KIDNEY Collaborative Group 2022 | Empagliflozin 10 mg qd + ACEI/ARB | 3304 | 63.9 ± 13.9 | 2207/1097 | NO | 92 |
|  | Placebo + ACEI/ARB | 3305 | 63.8 ± 13.9 | 2210/1095 | NO | 109 |
| Fried 2013 | Losartan 100 mg + Lisinopril 10-40 mg + Losartan daily | 724 | 64.7 ± 7.7 | 715/9 | 4.3 ± 0.5 | 72 |
|  | Losartan 100 mg + Placebo + Losartan daily | 724 | 64.5 ± 7.9 | 721/3 | 4.3 ± 0.5 | 32 |
| Kohan 2013 | Dapagliflozin 5-10 mg | 168 | 66 ± 8.3 | 111/57 | NO | 18 |
|  | Placebo | 84 | 67 ± 8.6 | 53/31 | NO | 13 |
| Bakris 2015 | Finerenone 7.5~20mg + ACEI/ARB qd | 727 | 63.26 ± 8.68 | 570/157 | NO | 8 |
|  | Placebo + ACEI/ARB | 94 | 64.34 ± 9.23 | 69/25 | NO | 0 |
| Chen 2018 | Irbesartan 150-300mg daily | 109 | 67.50 ± 4.01 | 55/54 | 4.19 ± 0.34 | 1 |
|  | Irbesartan 150-300mg + Spironolactone 20mg daily | 109 | 67 ± 4.49 | 51/58 | 4.16 ± 0.33 | 7 |
| Ruggenenti 2019 | Valsartan 160 mg daily | 36 | 63.9 ± 9.2 | 31/5 | 4.52 ± 0.80 | 3 |
|  | Benazepril 10 mg daily | 34 | 66.3 ± 7.1 | 30/4 | 4.36 ± 0.54 | 1 |
|  | Valsartan 40 mg + Benazepril 2.5 mg daily | 33 | 63.1 ± 9.0 | 27/6 | 4.57 ± 0.64 | 5 |
| Ito (Esaxerenone) 2019 | Esaxerenone 0.625-5 mg + ACEI/ARB daily | 285 | 65.3 ± 9.3 | 222/63 | 4.25 ± 0.3 | 13 |
|  | Placebo + ACEI/ARB | 73 | 66 ± 10 | 57/16 | 4.3 ± 0.3 | 1 |
| Perkovic 2019 | Canagliflozin 100 mg + ACEI/ARB qd | 2202 | 62.9 ± 9.2 | 1440/762 | NO | 151 |
|  | Placebo + ACEI/ARB | 2199 | 63.2 ± 9.2 | 1467/732 | NO | 181 |
| Wada 2020 | Apararenone 2.5-10 mg + ACEI/ARB qd | 220 | 61.8 | 167/53 | 4.28 ± 0.28 | 5 |
|  | Placebo + ACEI/ARB | 72 |  | 54/19 | 4.24 ± 0.31 | 0 |
| Rossing 2021 | Finerenone 10-20mg + SGLT-2i+ACEI/ARB | 259 | 63.1 ± 9.6 | 188/71 | 4.24 ± 0.43 | 10 |
|  | Placebo+SGLT-2i + ACEI/ARB |  |  |  | 4.33 ± 0.41 | 4 |
|  | Finerenone 10-20mg+ACEI/ARB | 5415 | 65.7 ± 9.0 | 3795/1620 | 4.38 ± 0.46 | 506 |
|  | Placebo+ACEI/ARB |  |  |  | 4.38 ± 0.46 | 251 |
| Pitt 2021 | Finerenone 10-20 mg qd + ACEI/ARB | 3686 | 64.1 ± 9.7 | 2528/1158 | 4.33 ± 0.43 | 396 |
|  | Placebo + ACEI/ARB | 3666 | 64.1±10.0 | 2577/1089 | 4.33 ± 0.43 | 193 |
| Shikata 2022 | Esaxerenone 1.25–2.5 mg + SGLT-2i+ACEI/ARB daily | 61 | 59.6 ± 11 | 42/19 | 4.3 ± 0.25 | 4 |
|  | Placebo+SGLT-2i + ACEI/ARB | 56 | 61 ± 9 | 43/13 | 4.3 ± 0.3 | 3 |
|  | Esaxerenone 1.25–2.5 mg + ACEI/ARB daily | 218 | 67 ± 8 | 165/53 | 4.35 ± 0.3 | 57 |
|  | Placebo + ACEI/ARB | 171 | 67 ± 9 | 137/34 | 4.4 ± 0.3 | 11 |
| Tuttle 2022 | Empagliflozin 10 mg or 25 mg daily | 1519 | 67.4 ± 8.0 | 971/548 | NO | 53 |
|  | Placebo | 848 | 67.2 ± 8.3 | 555/293 | NO | 44 |
| Makhlough 2014 | Spironolactone 25 mg qd + half a tablet Placebo bid | 30 | 52.3 ± 10.61 | 7/23 | 4.49 ± 0.31 | 0 |
|  | Spironolactone 25 mg qd + Losartan 12.5 mg bid | 30 | 51.2 ± 12.29 | 8/22 | 4.32 ± 0.35 | 1 |
| Ravid 1993 | Enalapril 10 mg daily | 49 | 43.5 ± 3 | 21/28 | NO | NO |
|  | Placebo | 45 | 44.8 ± 3.5 | 21/24 | NO | NO |
| Trevisan 1995 | Ramipril 1.25 mg qd | 60 | 56 ± 7 | 44/16 | NO | NO |
|  | Placebo | 62 | 58 ± 7 | 50/12 | NO | NO |
| Vongterapak 1997 | Ramipril 1.25 mg | 16 | 41 ± 10.4 | 11/5 | 4.9 ± 0.7 | NO |
|  | Placebo | 12 | 54 ± 14.9 | 8/4 | 4.8 ± 0.6 | NO |
| Hoque 2009 | Enalapril 5-40 mg daily | 10 | 45-75 | NO | NO | NO |
|  | Losartan 25-200 mg daily | 8 |  |  |  |  |
| Ziaee 2013 | Spironolactone + enalapril | 29 | 53.10 ± 4.93 | 17/12 | 4.11 ± 0.23 | NO |
|  | Enalapril 25 mg | 31 | 53.03 ± 5.25 | 20/11 | 4.04 ± 0.30 | NO |
| Imbalzano 2014 | Aliskiren 300 mg | 63 | 67.2 ± 8.6 | 33/30 | 4.03 ± 0.6 | NO |
|  | Ramipril 10 mg or Losartan 100 mg | 63 | 66.4 ± 9.2 | 32/31 | 4.04 ± 0.5 | NO |
| Ito (Imarikiren) 2019 | Imarikiren | 275 | 61 ± 10 | 219/56 | 4.0 ± 0.3 | 1 |
|  | Candesartan Cilexetil 8 mg | 70 | 62 ± 9 | 56/14 | 4.1 4 ± 0.35 | 0 |
|  | Placebo | 66 | 62 ± 8 | 51/15 | 4.12 ± 0.3 | 0 |
| Marre 1988 | ACEI | 10 | 39.1 ± 10.96 | 12/8 | 4.1 ± 1.11 | NO |
|  | Placebo | 10 |  |  | 4 ± 0.52 | NO |

**Supplementary Table S4: Study level risk of bias assessment using Cochrane risk of bias tool for assessing risk of bias of**

**included trials.**

| Study (Author year) | random sequence generation | allocation sequence concealment | blinding of participants and personnel | blinding of outcome assessment | Incomplete outcome data | Selective reporting | other bias |
| --- | --- | --- | --- | --- | --- | --- | --- |
| Mokadem 2020 | low | unclear | high | unclear | high | low | low |
| Bauer 1992 | unclear | unclear | low | unclear | low | low | low |
| Lewis 1993 | low | low | low | low | low | low | high |
| Lewis 2001 | unclear | low | low | unclear | unclear | low | high |
| Meiracker 2006 | low | unclear | low | unclear | high | high | low |
| Tong 2006 | unclear | low | low | unclear | low | low | high |
| Epstein 2006 | unclear | unclear | low | low | low | low | high |
| Winkelmayer 2006 | high | high | high | unclear | unclear | high | low |
| Parving 2008 | low | unclear | low | low | low | low | high |
| Mehdi 2009 | low | unclear | low | unclear | high | low | low |
| Imai 2009 | low | low | low | low | low | low | high |
| Parving 2012 | low | low | low | low | low | low | low |
| Esteghamati 2013 | low | unclear | high | low | low | low | low |
| Juarez 2013 | low | low | high | low | low | low | low |
| The EMPA-KIDNEY Collaborative Group 2022 | low | low | low | unclear | low | low | low |
| Fried 2013 | unclear | unclear | low | low | low | low | low |
| Kohan 2013 | unclear | unclear | low | unclear | low | low | low |
| Bakris 2015 | low | low | low | low | low | low | high |
| Chen 2018 | low | unclear | high | low | low | low | high |
| Ruggenenti 2019 | low | low | high | unclear | low | low | low |
| Ito (Esaxerenone) 2019 | unclear | unclear | unclear | unclear | high | high | high |
| Perkovic 2019 | low | unclear | low | low | low | low | low |
| Wada 2020 | low | unclear | low | low | low | low | high |
| Rossing 2021 | high | high | high | low | high | high | high |
| Pitt 2021 | low | unclear | low | high | high | low | high |
| Shikata 2022 | high | high | high | unclear | high | low | low |
| Tuttle 2022 | high | high | high | low | high | low | unclear |
| Makhlough 2014 | unclear | unclear | low | unclear | low | low | low |
| Ravid 1993 | low | high | low | low | low | low | low |
| Trevisan 1995 | unclear | low | low | low | low | low | low |
| Vongterapak 1997 | unclear | unclear | low | unclear | low | high | high |
| Hoque 2009 | unclear | unclear | low | low | low | low | high |
| Ziaee 2013 | unclear | high | high | unclear | low | low | low |
| Imbalzano 2014 | high | unclear | high | unclear | unclear | unclear | low |
| Ito (Imarikiren) 2019 | low | low | low | unclear | low | low | low |
| Marre 1988 | unclear | unclear | low | unclear | unclear | low | low |

**Supplementary Table S5: Side-splitting approach to assess inconsistency for** **hyperkalemia.**

| Side | Direct | | Indirect | | Difference | | P>\|z\| | tau |
| --- | --- | --- | --- | --- | --- | --- | --- | --- |
|  | Coef. | Std. Err. | Coef. | Std. Err. | Coef. | Std. Err. | |  |
| A B | 0.1244257 | 0.4585159 | 1.338873 | 0.897978 | -1.214447 | 1.031594 | 0.239 | 0.248457 |
| A D | 0.795181 | 1.291112 | 0.8626143 | 0.555189 | -0.0674333 | 1.399608 | 0.962 | 0.290647 |
| A H | 0.172873 | 0.5306637 | 0.4641373 | 0.626913 | -0.2912643 | 0.822805 | 0.723 | 0.291726 |
| A I | -0.0444549 | 1.474109 | 0.1168053 | 1.56793 | -0.1612602 | 2.152394 | 0.94 | 0.289939 |
| A J | -0.146018 | 0.6049891 | -0.8946764 | 0.582339 | 0.7486584 | 0.837814 | 0.372 | 0.284158 |
| B C | -0.835715 | 0.1793845 | 1.298203 | 0.757198 | -2.133918 | 0.783328 | **0.006** | 1.75E-06 |
| B D | 1.342969 | 0.8063175 | 0.2968154 | 0.320959 | 1.046154 | 0.86631 | 0.227 | 0.261164 |
| B H | 0.0367635 | 0.4553591 | -0.6006375 | 0.869568 | 0.637401 | 0.99393 | 0.521 | 0.28484 |
| C D * | 0.8312823 | 0.0595029 | 2.293111 | 0.553407 | -1.461829 | 0.558001 | **0.009** | 9.49E-06 |
| C E * | -0.1312307 | 0.3878933 | 0.296273 | 0.668903 | -0.4275037 | 0.674503 | 0.526 | 3.25E-01 |
| C F * | 0.2844758 | 0.2538065 | 1.049575 | 1334.636 | -0.7650992 | 1334.636 | 1 | 2.84E-01 |
| C G * | -0.3313855 | 0.2215768 | -0.4712918 | 0.72343 | 0.1399063 | 0.719658 | 0.846 | 3.21E-01 |
| D E * | -1.214084 | 0.3734589 | -1.07418 | 0.730904 | -0.1399042 | 0.719664 | 0.846 | 3.21E-01 |
| D G * | -1.701115 | 0.4061329 | -1.254606 | 0.356623 | -0.4465092 | 0.502875 | 0.375 | 2.84E-01 |
| D H | -2.047006 | 1.106345 | -0.2021121 | 0.514591 | -1.844894 | 1.220165 | 0.131 | 2.47E-01 |
| D I * | -0.8018718 | 1.046493 | -0.9807553 | 2.689832 | 0.1788836 | 2.772494 | 0.949 | 2.91E-01 |
| E G * | -0.764342 | 0.475789 | 0.9409923 | 0.526039 | -1.705334 | 0.805581 | 0.034 | 1.62E-07 |
| H J * | -0.9192074 | 0.2963842 | -0.1705467 | 0.787972 | -0.7486606 | 0.837814 | 0.372 | 2.84E-01 |
| H K * | -0.656513 | 1.7607 | -2.593222 | 3.411602 | 1.936709 | 4.093948 | 0.636 | 2.86E-01 |
| J K * | -0.7788026 | 1.761232 | 1.157907 | 3.410779 | -1.93671 | 4.093948 | 0.636 | 2.86E-01 |
| J L * | -0.4843755 | 0.2901812 | 1.090902 | 1315.343 | -1.575277 | 1315.343 | 0.999 | 2.84E-01 |
| * Warning: all the evidence about these contrasts comes from the trials which directly compare them. | | | | | | | | |

A=ACEI, B=ACEI + ARB, C=ACEI/ARB, D=ACEI/ARB + MRA, E=ACEI/ARB + MRA + SGLT-2i, F=ACEI/ARB + RI, G=ACEI/ARB + SGLT-2i, H=ARB, I=MRA, J=Placebo, K=RI, L=SGLT-2i.

**Supplementary Table S6: Side-splitting approach to assess inconsistency for serum potassium levels.**

| Side | Direct | | Indirect | | Difference | | P>\|z\| | tau |
| --- | --- | --- | --- | --- | --- | --- | --- | --- |
|  | Coef. | Std. Err. | Coef. | Std. Err. | Coef. | Std. Err. |  |  |
| A D | 0.2339419 | 0.09623 | 0.10564 | 0.11971 | 0.1283 | 0.15466 | 0.407 | 0.07464 |
| A H | -0.2 | 0.20412 | 0.00373 | 0.08324 | -0.2037 | 0.22044 | 0.355 | 0.07526 |
| A I | 0.0601654 | 0.14688 | -0.0049 | 0.14853 | 0.06505 | 0.20877 | 0.755 | 0.07642 |
| A J | -0.1446038 | 0.05027 | -0.1082 | 0.14873 | -0.0364 | 0.15829 | 0.818 | 0.0769 |
| B C * | -0.1850767 | 0.07424 | -0.2696 | 0.40559 | 0.0845 | 0.41814 | 0.84 | 0.07581 |
| B D | 0.0277013 | 0.19847 | 0.00944 | 0.08994 | 0.01826 | 0.22098 | 0.934 | 0.07638 |
| C D * | 0.1882398 | 0.0361 | 0.46068 | 0.16832 | -0.2724 | 0.17241 | 0.114 | 0.06468 |
| C E * | 0.2 | 0.09138 | 0.27067 | 0.15502 | -0.0707 | 0.17471 | 0.686 | 0.07984 |
| C F * | 0.09 | 0.13932 | 0.03611 | 41.7269 | 0.05389 | 41.7272 | 0.999 | 0.07438 |
| C G * | 0.0350702 | 0.05928 | -0.0898 | 0.17469 | 0.12488 | 0.18199 | 0.493 | 0.07711 |
| D E * | -0.01 | 0.08836 | 0.11486 | 0.16552 | -0.1249 | 0.18199 | 0.493 | 0.07711 |
| D G * | -0.2541642 | 0.08112 | -0.098 | 0.07994 | -0.1562 | 0.1124 | 0.165 | 0.0681 |
| D H | -0.17 | 0.08078 | -0.3144 | 0.13425 | 0.14439 | 0.15668 | 0.357 | 0.07445 |
| D I * | -0.1663556 | 0.09588 | -0.0548 | 0.31468 | -0.1116 | 0.3326 | 0.737 | 0.07614 |
| E G * | -0.25 | 0.08537 | 0.07111 | 0.17794 | -0.3211 | 0.19801 | 0.105 | 0.06525 |
| H J * | -0.0999999 | 0.10293 | -0.1364 | 0.12026 | 0.03643 | 0.15829 | 0.818 | 0.0769 |
| H K * | -0.1 | 0.09384 | -0.1728 | 0.29574 | 0.07285 | 0.31659 | 0.818 | 0.0769 |
| J K * | 6.63E-09 | 0.09281 | 0.07289 | 0.2967 | -0.0729 | 0.31657 | 0.818 | 0.0769 |

A=ACEI, B=ACEI + ARB, C=ACEI/ARB, D=ACEI/ARB + MRA, E=ACEI/ARB + MRA + SGLT-2i, F=ACEI/ARB + RI, G=ACEI/ARB + SGLT-2i, H=ARB, I=MRA, J=Placebo, K=RI.

**Supplementary Table S7: Percentage contribution of each direct estimate derived from direct (blue) and indirect (red) comparisons for hyperkalemia (upper-right) and serum potassium (bottom-left).**

| SGLT-2i | | 0 | 100 | 100 | 0 | 0 | 100 | 0 | 100 | 0 | 100 | 0 | 100 | 0 | 100 | 0 | 100 | 0 | 100 | 0 | 100 | 0 | 100 |
| --- | --- | --- | --- | --- | --- | --- | --- | --- | --- | --- | --- | --- | --- | --- | --- | --- | --- | --- | --- | --- | --- | --- | --- |
| — | — | RI | | 33 | 67 | 0 | 100 | 33 | 67 | 0 | 100 | 0 | 100 | 0 | 100 | 0 | 100 | 0 | 100 | 0 | 100 | 0 | 100 |
| — | — | 44 | 56 | Placebo | | 0 | 100 | 93 | 7 | 0 | 100 | 0 | 100 | 0 | 100 | 0 | 100 | 0 | 100 | 0 | 100 | 13.2 | 86.8 |
| — | — | 0 | 100 | 0 | 100 | MRA | | 0 | 100 | 0 | 100 | 0 | 100 | 0 | 100 | 33.9 | 66.1 | 0 | 100 | 0 | 100 | 12.5 | 87.5 |
| — | — | 41.5 | 58.5 | 22.5 | 77.5 | 0 | 100 | ARB | | 0 | 100 | 0 | 100 | 0 | 100 | 5.1 | 94.9 | 0 | 100 | 42 | 58 | 35.1 | 64.9 |
| — | — | 0 | 100 | 0 | 100 | 0 | 100 | 0 | 100 | ACEI/ARB+SGLT-2i | | 0 | 100 | 9.8 | 91.2 | 8.4 | 91.6 | 78.9 | 21.1 | 0 | 100 | 0 | 100 |
| — | — | 0 | 100 | 0 | 100 | 0 | 100 | 0 | 100 | 0 | 100 | ACEI/ARB+RI | | 0 | 100 | 0 | 100 | 100 | 0 | 0 | 100 | 0 | 100 |
| — | — | 0 | 100 | 0 | 100 | 0 | 100 | 0 | 100 | 27.5 | 72.5 | 0 | 100 | ACEI/ARB+MRA+SGLT-2i | | 29 | 71 | 29.2 | 70.8 | 0 | 100 | 0 | 100 |
| — | — | 0 | 100 | 0 | 100 | 49.6 | 50.4 | 73.9 | 26.1 | 39.9 | 60.1 | 0 | 100 | 34.9 | 65.1 | ACEI/ARB+MRA | | 58.5 | 41.5 | 5 | 95 | 4.4 | 95.6 |
| — | — | 0 | 100 | 0 | 100 | 0 | 100 | 0 | 100 | 15.2 | 84.8 | 100 | 0 | 35.6 | 64.4 | 42.8 | 57.2 | ACEI/ARB | | 65.7 | 34.3 | 0 | 100 |
| — | — | 0 | 100 | 0 | 100 | 0 | 100 | 0 | 100 | 0 | 100 | 0 | 100 | 0 | 100 | 1.8 | 98.2 | 95.8 | 4.2 | ACEI+ARB | | 9.4 | 90.6 |
| — | — | 0 | 100 | 15.3 | 84.7 | 0 | 100 | 3.3 | 96.7 | 0 | 100 | 0 | 100 | 0 | 100 | 19.7 | 80.3 | 0 | 100 | 0 | 100 | ACEI | |

ACEI: angiotensin-converting enzyme inhibitors, ARB: angiotensin receptor blockers, MRA: mineralocorticoid receptor antagonists, RI: renin inhibitors.

**Table S8 Subgroup analysis presenting odds ratio with 95% CI for hyperkalemia between interventions, stratified by eGFR (upper-right): ≥ 60 mL/min/1.73 m2 and eGFR (bottom-left): < 60 mL/min/1.73 m2.**

| RI | 2.09  (0.08,52.24) | 7.25  (0.14, 383.53) | 2.01  (0.08, 50.42) | 3.12  (0.07, 138.53) | 4.87(0.12,199.64) | 3.87  (0.09, 164.72) | 16.03  (0.46, 555.49) | 5.61  (0.16, 201.66) | —— | 7.63  (0.19, 301.07) |
| --- | --- | --- | --- | --- | --- | --- | --- | --- | --- | --- |
| —— | Placebo | 3.48  (0.11, 108.73) | 0.97  (0.05, 20.06) | 1.50  (0.05, 42.11) | 2.33  (0.09,60.02) | 1.86  (0.07, 49.77) | 7.68  (0.36, 162.74) | 2.69  (0.12, 59.42) | —— | 3.66  (0.23, 57.59) |
| —— | —— | MRA | 0.28 (0.02,4.79) | 0.43  (0.04, 5.02) | 0.67  (0.07, 6.92) | 0.53  (0.05, 5.83) | 2.21  (0.28, 17.24) | 0.77  (0.09, 6.42) | —— | 1.05  (0.08, 14.61) |
| —— | —— | —— | ARB | 1.55  (0.13, 18.32) | 2.42  (0.23,25.25) | 1.92  (0.17, 21.28) | **7.96**  **(1.00, 63.04)** | 2.79  (0.33, 23.46) | —— | 3.79  (0.23, 62.73) |
| —— | —— | —— | 1.51 (0.31, 7.40) | ACEI/ARB+SGLT-2i | 1.56  (0.28, 8.59) | 1.24  (0.23, 6.69) | **5.14**  **(1.33,19.82)** | 1.80 (0.45,7.24) | —— | 2.45  (0.17, 34.88) |
| —— | —— | —— | 0.78  (0.16, 3.76) | **0.51**  **(0.42, 0.62)** | ACEI/ARB+RI | 0.80  (0.16, 3.97) | **3.29**  **(1.09, 9.95)** | 1.15  (0.43, 3.09) | —— | 1.57  (0.12, 19.92) |
| —— | —— | —— | 1.40  (0.25, 7.73) | 0.92  (0.47, 1.83) | 1.81  (0.93, 3.51) | ACEI/ARB+MRA+SGLT-2i | **4.14**  **(1.22, 14.07)** | 1.45  (0.41, 5.16) | —— | 1.97  (0.15, 26.41) |
| —— | —— | —— | 0.53  (0.11, 2.60) | **0.35**  **(0.28, 0.45)** | 0.69  (0.57, 0.83) | 0.38  (0.20, 0.73) | ACEI/ARB+MRA | **0.35**  **(0.21, 0.58)** | —— | 0.48  (0.05, 4.70) |
| —— | —— | —— | 1.22  (0.25, 5.89) | **0.80**  **(0.68, 0.96)** | **1.57**  **(1.43, 1.72)** | 0.87 (0.45, 1.68) | **2.28**  **(1.94, 2.68)** | ACEI/ARB | —— | 1.36  (0.13, 14.17) |
| —— | —— | —— | 0.51  (0.11, 2.32) | **0.34**  **(0.21, 0.53)** | 0.66  (0.42, 1.02) | **0.36 (0.17, 0.80)** | 0.96  (0.60, 1.51) | 0.42  (0.27, 0.64) | ACEI+ARB | —— |
| —— | —— | —— | 3.00  (0.30, 30.31) | 1.98  (0.21, 18.84) | 3.87  (0.41, 36.62) | 2.14  (0.21, 22.26) | 5.62  (0.59, 53.46) | 2.47  (0.26, 23.30) | 5.89(0.65,53.38) | ACEI |

ACEI: angiotensin-converting enzyme inhibitors, ARB: angiotensin receptor blockers, MRA: mineralocorticoid receptor antagonists, RI: renin inhibitors.

**Table S9 Subgroup analysis presenting odds ratio with 95% CI for hyperkalemia between interventions, stratified by sample size (upper-right): > 100 and sample size (bottom-left): ≥ 100.**

| SGLT-2i | 0.65  (0.03, 12.80) | 1.62  (0.94, 2.79) | 1.11  (0.21, 5.86) | 0.49  (0.08, 2.95) | 1.06  (0.16, 6.86) | 0.68  (0.09, 5.14) | 1.89  (0.28, 12.79) | 0.68  (0.11, 4.06) | 1.40  (0.27, 7.31) | 1.09  (0.27, 4.50) |
| --- | --- | --- | --- | --- | --- | --- | --- | --- | --- | --- |
| 0.85  (0.05, 14.04) | RI | 2.47  (0.13, 45.97) | 1.70  (0.09, 31.54) | 0.75  (0.04, 15.82) | 1.62  (0.07, 35.55) | 1.04  (0.04, 24.87) | 2.89  (0.13, 64.46) | 1.03  (0.05, 21.68) | 2.14  (0.11, 41.86) | 1.67  (0.09, 31.56) |
| **0.63**  **(0.44, 0.91)** | 0.75  (0.05, 12.08) | Placebo | 0.69  (0.14, 3.29) | 0.30  (0.05, 1.67) | 0.65  (0.11, 3.87) | 0.42  (0.06, 2.90) | 1.17  (0.19, 7.17) | 0.42  (0.08, 2.28) | 0.86  (0.18, 4.09) | 0.67  (0.18, 2.49) |
| **0.27**  **(0.17, 0.43)** | 0.32  (0.02, 5.15) | **0.43**  **(0.33, 0.56)** | ARB | 0.44  (0.15, 1.26) | 0.95  (0.30, 2.99) | 0.61  (0.16, 2.37) | 1.70  (0.53, 5.48) | 0.61  (0.22, 1.68) | 1.26  (0.56, 2.85) | 0.98  (0.37, 2.61) |
| **0.10**  **(0.01, 0.87)** | 0.12  (0.00, 3.87) | 0.16  (0.02, 1.33) | 0.37 (0.04,3.06) | ACEI/ARB+SGLT-2i | **2.17**  **(1.12, 4.18)** | 1.39  (0.53, 3.60) | **3.87**  **(1.98, 7.54)** | 1.38  (0.92, 2.06) | **2.86**  **(1.37, 5.98)** | 2.23  (0.70, 7.09) |
| **0.05**  **(0.01, 0.45)** | 0.06  (0.00, 1.99) | **0.08**  **(0.01, 0.68)** | 0.19 (0.02,1.57) | **0.52 (0.43,0.63)** | ACEI/ARB+RI | 0.64  (0.24, 1.71) | 1.78  (0.90, 3.55) | 0.64  (0.38, 1.07) | 1.32  (0.57, 3.07) | 1.03  (0.29, 3.61) |
| **0.09**  **(0.01, 0.88)** | 0.11  (0.00, 3.78) | 0.14  (0.02, 1.34) | 0.34  (0.04, 3.09) | 0.93  (0.47, 1.82) | 1.79  (0.92, 3.47) | ACEI/ARB+MRA+SGLT-2i | **2.79**  **(1.25, 6.25)** | 1.00  (0.43, 2.30) | 2.06  (0.69, 6.21) | 1.61  (0.37, 7.02) |
| **0.03**  **(0.00, 0.30)** | 0.04  (0.00, 1.36) | **0.06**  **(0.01, 0.46)** | 0.13  (0.02, 1.07) | **0.35**  **(0.29, 0.43)** | **0.68**  **(0.59, 0.79)** | **0.38**  **(0.20, 0.73)** | ACEI/ARB+MRA | **0.36**  **(0.23, 0.56)** | 0.74  (0.31, 1.74) | 0.58  (0.16, 2.14) |
| **0.08**  **(0.01, 0.69)** | 0.09  (0.00, 3.10) | 0.13  (0.01, 1.06) | 0.29  (0.04, 2.44) | **0.80**  **(0.68, 0.96)** | 1.55  (1.42, 1.70) | 0.87  (0.45, 1.67) | **2.28**  **(2.03, 2.56)** | ACEI/ARB | **2.07**  **(1.06, 4.03)** | 1.62  (0.52, 5.06) |
| **0.04**  **(0.00, 0.32)** | 0.04  (0.00, 1.43) | **0.06**  **(0.01, 0.50)** | 0.13  (0.02, 1.14) | **0.36**  **(0.24, 0.54)** | 0.71  (0.49, 1.02) | **0.39**  **(0.19, 0.83)** | 1.03  (0.71, 1.51) | **0.45**  **(0.32, 0.65)** | ACEI+ARB | 0.78  (0.31, 1.94) |
| 0.11  (0.01, 2.17) | 0.13  (0.00, 7.48) | 0.17  (0.01, 3.36) | 0.39  (0.02, 7.96) | 1.07  (0.03, 42.46) | 2.08  (0.05, 81.92) | 1.16  (0.03, 48.33) | 3.04  (0.08, 119.77) | 1.34  (0.03, 52.65) | 2.94  (0.07, 118.00) | ACEI |

ACEI: angiotensin-converting enzyme inhibitors, ARB: angiotensin receptor blockers, MRA: mineralocorticoid receptor antagonists, RI: renin inhibitors.

**Table S10 Subgroup analysis presenting odds ratio with 95% CI for hyperkalemia between interventions, stratified by duration (upper-right): weeks≥48 weeks and duration (bottom-left): weeks<48 weeks.**

| SGLT-2i | 0.69  (0.02, 21.46) | **1.51**  **(1.01, 2.28)** | 5.72 (0.09, 370.09) | 1.34  (0.03, 69.99) | —— | 5.44 (0.08, 355.51) | —— | 12.77 (0.23, 708.83) | 6.28 (0.10, 385.67) | —— | 5.86 (0.24, 144.27) |
| --- | --- | --- | --- | --- | --- | --- | --- | --- | --- | --- | --- |
| —— | RI | 2.18  (0.07, 65.68) | 8.24 (0.04, 1766.51) | 1.93 (0.06, 58.06) | —— | 7.83 (0.04, 1693.18) | —— | 18.37 (0.10, 3503.62) | 9.04 (0.04, 1862.36) | —— | 8.44 (0.08, 889.39) |
| 0.53  (0.17, 1.65) | —— | Placebo | 3.78 (0.06, 239.62) | 0.88 (0.02, 45.27) | —— | 3.60 (0.06, 230.19) | —— | 8.43 (0.16, 458.59) | 4.15 (0.07, 249.65) | —— | 3.87(0.16,92.84) |
| —— | —— | —— | MRA | 0.23  (0.00, 71.26) | —— | 0.95  (0.09, 9.63) | —— | 2.23 (0.30,16.60) | 1.10  (0.12, 9.92) | —— | 1.02 (0.07, 14.79) |
| **0.23**  **(0.06, 0.85)** | —— | **0.43**  **(0.22, 0.85)** | —— | ARB | —— | 4.06 (0.01, 1245.85) | —— | 9.53 (0.03, 2598.20) | 4.69 (0.02, 1374.36) | —— | 4.38 (0.03, 687.92) |
| 0.55  (0.10, 2.98) | —— | 1.03  (0.30, 3.62) | —— | 2.42 (0.80,7.33) | ACEI/ARB+SGLT-2i | —— | —— | —— | —— | —— | —— |
| 0.25  (0.04, 1.51) | —— | 0.47  (0.11, 1.90) | —— | 1.09 (0.30,3.91) | 0.45 (0.18,1.14) | ACEI/ARB+RI | —— | 2.35  (0.74, 7.43) | 1.15  (0.43, 3.09) | —— | 1.08 (0.07, 15.79) |
| 0.38  (0.06, 2.35) | —— | 0.71  (0.17, 2.98) | —— | 1.67  (0.46, 6.08) | 0.69  (0.28, 1.71) | 1.53  (0.50, 4.66) | ACEI/ARB+MRA+SGLT-2i | **——** | —— | —— | —— |
| **0.11**  **(0.02, 0.61)** | —— | **0.20**  **(0.05, 0.75)** | —— | 0.47  (0.15, 1.50) | **0.20**  **(0.09, 0.41)** | 0.43  (0.17, 1.13) | **0.28**  **(0.13, 0.63)** | ACEI/ARB+MRA | 0.49  (0.20, 1.22) | —— | 0.46 (0.04, 5.19) |
| 0.39  (0.08, 1.99) | —— | 0.73  (0.23, 2.36) | —— | 1.71  (0.62, 4.71) | 0.71  (0.43, 1.16) | 1.57  (0.72, 3.41) | 1.03  (0.46, 2.28) | **3.61**  **(2.07, 6.30)** | ACEI/ARB | —— | 0.93 (0.07, 12.43) |
| 0.20  (0.04, 0.95) | —— | 0.38  (0.13, 1.08) | —— | 0.89  (0.37, 2.13) | **0.37**  **(0.17, 0.79)** | 0.81  (0.31, 2.16) | 0.53  (0.20, 1.42) | 1.87  (0.85, 4.11) | **0.52**  **(0.29, 0.94)** | ACEI+ARB | —— |
| 0.33  (0.08, 1.45) | —— | 0.62  (0.24, 1.60) | —— | 1.45  (0.59, 3.56) | 0.60  (0.18, 1.99) | 1.33  (0.34, 5.16) | 0.87  (0.22, 3.47) | 3.06 (0.86, 10.88) | 0.85  (0.28, 2.57) | 1.63 (0.63, 4.28) | ACEI |

ACEI: angiotensin-converting enzyme inhibitors, ARB: angiotensin receptor blockers, MRA: mineralocorticoid receptor antagonists, RI: renin inhibitors.

**Table S11 Network league table of MRA subgroups regarding the risk of hyperkalemia**

| SP | 0.09(0.00,8.87) | 0.27(0.00, 15.30) | 2.10(0.07,65.12) | 0.06(0.00,2.40) | 0.11(0.00,4.68) | 0.29(0.01,11.54) | 0.11(0.00,5.35) | 0.57(0.01,23.78) | 0.21(0.00,9.87) | 0.45(0.00,48.58) | 0.13(0.00,5.20) | 0.63(0.02,25.65) | 0.10(0.00,9.28) |
| --- | --- | --- | --- | --- | --- | --- | --- | --- | --- | --- | --- | --- | --- |
| —— | EP | 2.98(0.07, 119.95) | **23.09(1.12,478.04)** | 0.61(0.04,10.58) | 1.23(0.07,20.45) | 3.21(0.21,49.48) | 1.25(0.06,24.24) | 6.30(0.38,103.66) | 2.30(0.19,27.28) | 4.89(0.09,266.53) | 1.45(0.09,22.29) | 6.93(0.31,155.40) | 1.05(0.06,17.78) |
| —— | —— | ARB | 7.74(0.94,64.12) | 0.21(0.02,2.80) | 0.41(0.03,5.40) | 1.08(0.09,12.97) | 0.42(0.03,6.49) | 2.11(0.16,27.35) | 0.77(0.05,11.99) | 1.64(0.04,75.85) | 0.49(0.04,5.84) | 2.33(0.19,29.22) | 0.35(0.01,14.12) |
| —— | —— | —— | ACEI/ARB+SP | **0.03(0.01,0.12)** | **0.05(0.01,0.23)** | **0.14(0.04,0.52)** | **0.05(0.01,0.31)** | 0.27(0.06,1.16) | **0.10(0.02,0.57)** | 0.21(0.01,5.19) | **0.06(0.02,0.23)** | 0.30(0.07,1.21) | **0.05(0.00,0.94)** |
| —— | —— | —— | —— | ACEI/ARB+SGLT-2i | 2.00(0.72,5.56) | **5.22(2.36,11.57)** | 2.03(0.55,7.49) | 10.25(4.30,24.45) | 3.74(0.92,15.27) | 7.95(0.39,163.79) | **2.36(1.07,5.21)** | 11.28(2.10,60.69) | 1.70(0.10,29.31) |
| —— | —— | —— | —— | —— | ACEI/ARB+FI+SGLT-2i | **2.61(1.36,5.01)** | 1.01(0.27,3.81) | 5.12(2.09,12.54) | 1.87(0.49,7.09) | 3.97(0.20,79.12) | 1.18(0.61,2.27) | 5.63(1.11,28.53) | 0.85(0.05,14.13) |
| —— | —— | —— | —— | —— | —— | ACEI/ARB+FI | 0.39(0.12,1.24) | 1.96(1.05,3.66) | 0.72(0.22,2.30) | 1.52(0.08,28.28) | **0.45(0.40,0.51)** | 2.16(0.49,9.57) | 0.33(0.02,5.03) |
| —— | —— | —— | —— | —— | —— | —— | ACEI/ARB+ES+SGLT-2i | **5.04(1.75,14.50)** | 1.84(0.36,9.45) | 3.91(0.17,90.25) | 1.16(0.37,3.67) | 5.55(0.85,36.32) | 0.84(0.04,16.26) |
| —— | —— | —— | —— | —— | —— | —— | —— | ACEI/ARB+ES | 0.36(0.10,1.36) | 0.78(0.04,15.32) | **0.23(0.12,0.42)** | 1.10(0.22,5.48) | 0.17(0.01,2.73) |
| —— | —— | —— | —— | —— | —— | —— | —— | —— | ACEI/ARB+EP | 2.13(0.09,49.23) | 0.63(0.20,2.02) | 3.02(0.46,19.86) | 0.46(0.04,5.41) |
| —— | —— | —— | —— | —— | —— | —— | —— | —— | —— | ACEI/ARB+AP | 0.30(0.02,5.49) | 1.42(0.05,37.50) | 0.21(0.00,11.68) |
| —— | —— | —— | —— | —— | —— | —— | —— | —— | —— | —— | ACEI/ARB | **4.78(1.08,21.09)** | 0.72(0.05,11.10) |
| —— | —— | —— | —— | —— | —— | —— | —— | —— | —— | —— | —— | ACEI+ARB | 0.15(0.01,3.38) |
| —— | —— | —— | —— | —— | —— | —— | —— | —— | —— | —— | —— | —— | ACEI |

ACEI: angiotensin-converting enzyme inhibitors, ARB: angiotensin receptor blockers, MRA: mineralocorticoid receptor antagonists, SP: Spironolactone, EP: Eplerenone, FI: Finerenone, ES: Esaxerenone, AP: Apararenone.

**Credibility of evidence**

**1. Hyperkalemia**

**1.1 Risk of Bias Chart showing the contribution of low, moderate, or high RoB comparisons to each network estimate.**

**
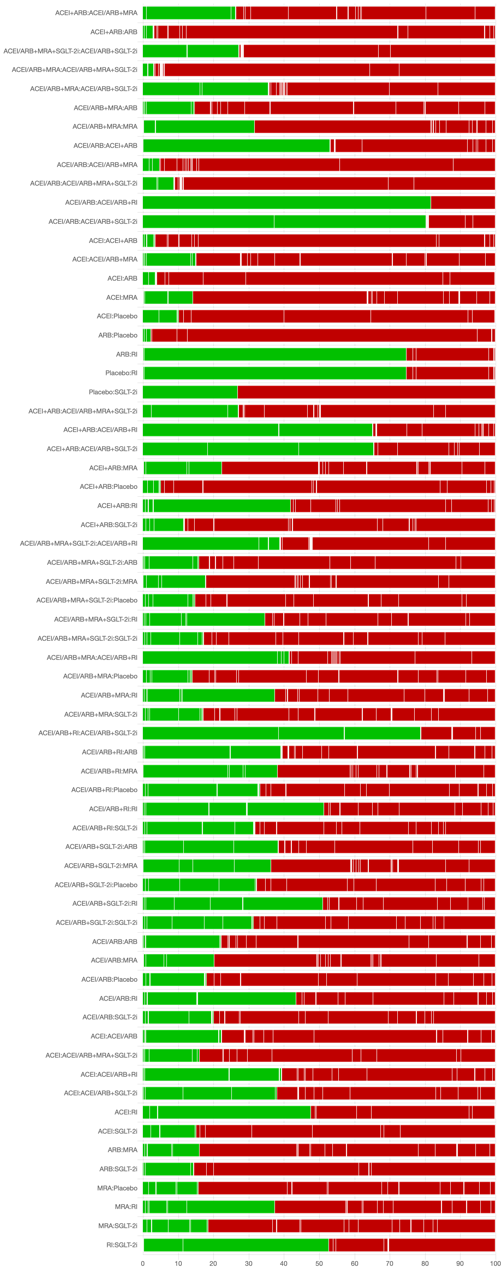
**

ACEI: angiotensin-converting enzyme inhibitors, ARB: angiotensin receptor blockers, MRA: mineralocorticoid receptor antagonists, RI: renin inhibitors.

**1.2 Supplementary Table S12: Evaluation of the Certainty of evidence Using CINEMA Framework.**

| Comparison | Number of studies | Within-study bias | Reporting bias | Indirectness | Imprecision | Heterogeneity | Incoherence | Confidence rating |
| --- | --- | --- | --- | --- | --- | --- | --- | --- |
| ACEI:ACEI+ARB | 2 | Major concerns | Low risk | No concerns | Major concerns | No concerns | No concerns | Very low |
| ACEI:ACEI/ARB+MRA | 1 | Major concerns | Low risk | No concerns | Some concerns | Some concerns | No concerns | Very low |
| ACEI:ARB | 2 | Major concerns | Low risk | No concerns | Major concerns | No concerns | No concerns | Very low |
| ACEI:MRA | 1 | Major concerns | Low risk | No concerns | Major concerns | No concerns | No concerns | Very low |
| ACEI:Placebo | 3 | Major concerns | Some concerns | No concerns | Major concerns | No concerns | No concerns | Very low |
| ACEI/ARB:ACEI+ARB | 3 | Some concerns | Low risk | No concerns | No concerns | No concerns | Major concerns | Very low |
| ACEI+ARB:ACEI/ARB+MRA | 1 | Some concerns | Some concerns | No concerns | Some concerns | Some concerns | Some concerns | Low |
| ACEI+ARB:ARB | 2 | Major concerns | Low risk | No concerns | Major concerns | No concerns | No concerns | Very low |
| ACEI/ARB:ACEI/ARB+MRA | 10 | Major concerns | Some concerns | No concerns | No concerns | No concerns | No concerns | Very low |
| ACEI/ARB:ACEI/ARB+MRA+SGLT-2i | 2 | Major concerns | Some concerns | No concerns | Major concerns | No concerns | No concerns | Very low |
| ACEI/ARB:ACEI/ARB+RI | 2 | No concerns | Low risk | No concerns | Some concerns | No concerns | Major concerns | Very low |
| ACEI/ARB:ACEI/ARB+SGLT-2i | 4 | No concerns | Low risk | No concerns | Some concerns | No concerns | No concerns | Moderate |
| ACEI/ARB+MRA:ACEI/ARB+MRA+SGLT-2i | 2 | Major concerns | Some concerns | No concerns | No concerns | No concerns | No concerns | Very low |
| ACEI/ARB+MRA:ACEI/ARB+SGLT-2i | 2 | Some concerns | Some concerns | No concerns | No concerns | No concerns | No concerns | Moderate |
| ACEI/ARB+MRA:ARB | 1 | Major concerns | Low risk | No concerns | Major concerns | No concerns | No concerns | Very low |
| ACEI/ARB+MRA:MRA | 2 | Some concerns | Low risk | No concerns | Major concerns | No concerns | No concerns | Very low |
| ACEI/ARB+MRA+SGLT-2i:ACEI/ARB+SGLT-2i | 2 | Some concerns | Some concerns | No concerns | Major concerns | No concerns | No concerns | Very low |
| ARB:Placebo | 3 | Major concerns | Some concerns | No concerns | No concerns | No concerns | No concerns | Very low |
| ARB:RI | 1 | Some concerns | Low risk | No concerns | Major concerns | No concerns | No concerns | Very low |
| Placebo:RI | 1 | Some concerns | Low risk | No concerns | Major concerns | No concerns | No concerns | Very low |
| Placebo:SGLT-2i | 2 | Some concerns | Low risk | No concerns | No concerns | Some concerns | Major concerns | Very low |
| ACEI:ACEI/ARB | 0 | Major concerns | Low risk | No concerns | Major concerns | No concerns | Major concerns | Very low |
| ACEI:ACEI/ARB+MRA+SGLT-2i | 0 | Major concerns | Low risk | No concerns | Major concerns | No concerns | Major concerns | Very low |
| ACEI:ACEI/ARB+RI | 0 | Some concerns | Low risk | No concerns | Major concerns | No concerns | Major concerns | Very low |
| ACEI:ACEI/ARB+SGLT-2i | 0 | Some concerns | Low risk | No concerns | Major concerns | No concerns | Major concerns | Very low |
| ACEI:RI | 0 | Some concerns | Low risk | No concerns | Major concerns | No concerns | Major concerns | Very low |
| ACEI:SGLT-2i | 0 | Major concerns | Low risk | No concerns | No concerns | Some concerns | Major concerns | Very low |
| ACEI+ARB:ACEI/ARB+MRA+SGLT-2i | 0 | Some concerns | Low risk | No concerns | No concerns | Some concerns | Major concerns | Very low |
| ACEI+ARB:ACEI/ARB+RI | 0 | Some concerns | Low risk | No concerns | Some concerns | Some concerns | Major concerns | Very low |
| ACEI+ARB:ACEI/ARB+SGLT-2i | 0 | Some concerns | Low risk | No concerns | No concerns | No concerns | Major concerns | Very low |
| ACEI+ARB:MRA | 0 | Major concerns | Low risk | No concerns | Major concerns | No concerns | Major concerns | Very low |
| ACEI+ARB:Placebo | 0 | Major concerns | Low risk | No concerns | No concerns | Some concerns | Major concerns | Very low |
| ACEI+ARB:RI | 0 | Some concerns | Low risk | No concerns | Major concerns | No concerns | Major concerns | Very low |
| ACEI+ARB:SGLT-2i | 0 | Major concerns | Low risk | No concerns | No concerns | No concerns | Major concerns | Very low |
| ACEI/ARB:ARB | 0 | Major concerns | Low risk | No concerns | Some concerns | Some concerns | Major concerns | Very low |
| ACEI/ARB:MRA | 0 | Major concerns | Low risk | No concerns | Major concerns | No concerns | Major concerns | Very low |
| ACEI/ARB:Placebo | 0 | Major concerns | Low risk | No concerns | Major concerns | No concerns | Major concerns | Very low |
| ACEI/ARB:RI | 0 | Some concerns | Low risk | No concerns | Major concerns | No concerns | Major concerns | Very low |
| ACEI/ARB:SGLT-2i | 0 | Major concerns | Low risk | No concerns | Major concerns | No concerns | Major concerns | Very low |
| ACEI/ARB+MRA:ACEI/ARB+RI | 0 | Some concerns | Low risk | No concerns | No concerns | No concerns | Major concerns | Very low |
| ACEI/ARB+MRA:Placebo | 0 | Major concerns | Low risk | No concerns | No concerns | No concerns | Major concerns | Very low |
| ACEI/ARB+MRA:RI | 0 | Some concerns | Low risk | No concerns | Major concerns | No concerns | Major concerns | Very low |
| ACEI/ARB+MRA:SGLT-2i | 0 | Major concerns | Low risk | No concerns | No concerns | No concerns | Major concerns | Very low |
| ACEI/ARB+MRA+SGLT-2i:ACEI/ARB+RI | 0 | Some concerns | Low risk | No concerns | Major concerns | No concerns | Major concerns | Very low |
| ACEI/ARB+MRA+SGLT-2i:ARB | 0 | Major concerns | Low risk | No concerns | Major concerns | No concerns | Major concerns | Very low |
| ACEI/ARB+MRA+SGLT-2i:MRA | 0 | Major concerns | Low risk | No concerns | Major concerns | No concerns | Major concerns | Very low |
| ACEI/ARB+MRA+SGLT-2i:Placebo | 0 | Major concerns | Low risk | No concerns | Major concerns | No concerns | Major concerns | Very low |
| ACEI/ARB+MRA+SGLT-2i:RI | 0 | Some concerns | Low risk | No concerns | Major concerns | No concerns | Major concerns | Very low |
| ACEI/ARB+MRA+SGLT-2i:SGLT-2i | 0 | Major concerns | Low risk | No concerns | Major concerns | No concerns | Major concerns | Very low |
| ACEI/ARB+RI:ACEI/ARB+SGLT-2i | 0 | No concerns | Low risk | No concerns | No concerns | No concerns | Major concerns | Very low |
| ACEI/ARB+RI:ARB | 0 | Some concerns | Low risk | No concerns | Major concerns | No concerns | Major concerns | Very low |
| ACEI/ARB+RI:MRA | 0 | Some concerns | Low risk | No concerns | Major concerns | No concerns | Major concerns | Very low |
| ACEI/ARB+RI:Placebo | 0 | Some concerns | Low risk | No concerns | Major concerns | No concerns | Major concerns | Very low |
| ACEI/ARB+RI:RI | 0 | Some concerns | Low risk | No concerns | Major concerns | No concerns | Major concerns | Very low |
| ACEI/ARB+RI:SGLT-2i | 0 | Some concerns | Low risk | No concerns | Some concerns | No concerns | Major concerns | Very low |
| ACEI/ARB+SGLT-2i:ARB | 0 | Some concerns | Low risk | No concerns | No concerns | Some concerns | Major concerns | Very low |
| ACEI/ARB+SGLT-2i:MRA | 0 | Some concerns | Low risk | No concerns | Major concerns | No concerns | Major concerns | Very low |
| ACEI/ARB+SGLT-2i:Placebo | 0 | Some concerns | Low risk | No concerns | Major concerns | No concerns | Major concerns | Very low |
| ACEI/ARB+SGLT-2i:RI | 0 | Some concerns | Low risk | No concerns | Major concerns | No concerns | Major concerns | Very low |
| ACEI/ARB+SGLT-2i:SGLT-2i | 0 | Some concerns | Low risk | No concerns | Major concerns | No concerns | Major concerns | Very low |
| ARB:MRA | 0 | Major concerns | Low risk | No concerns | Major concerns | No concerns | Major concerns | Very low |
| ARB:SGLT-2i | 0 | Major concerns | Low risk | No concerns | No concerns | No concerns | Major concerns | Very low |
| MRA:Placebo | 0 | Major concerns | Low risk | No concerns | Major concerns | No concerns | Major concerns | Very low |
| MRA:RI | 0 | Some concerns | Low risk | No concerns | Major concerns | No concerns | Major concerns | Very low |
| MRA:SGLT-2i | 0 | Major concerns | Low risk | No concerns | Major concerns | No concerns | Major concerns | Very low |
| RI:SGLT-2i | 0 | Some concerns | Low risk | No concerns | Major concerns | No concerns | Major concerns | Very low |

ACEI: angiotensin-converting enzyme inhibitors, ARB: angiotensin receptor blockers, MRA: mineralocorticoid receptor antagonists, RI: renin inhibitors.

**2. Serum potassium**

**2.1 Risk of Bias Chart showing the contribution of low, moderate, or high RoB comparisons to each network estimate.**

**
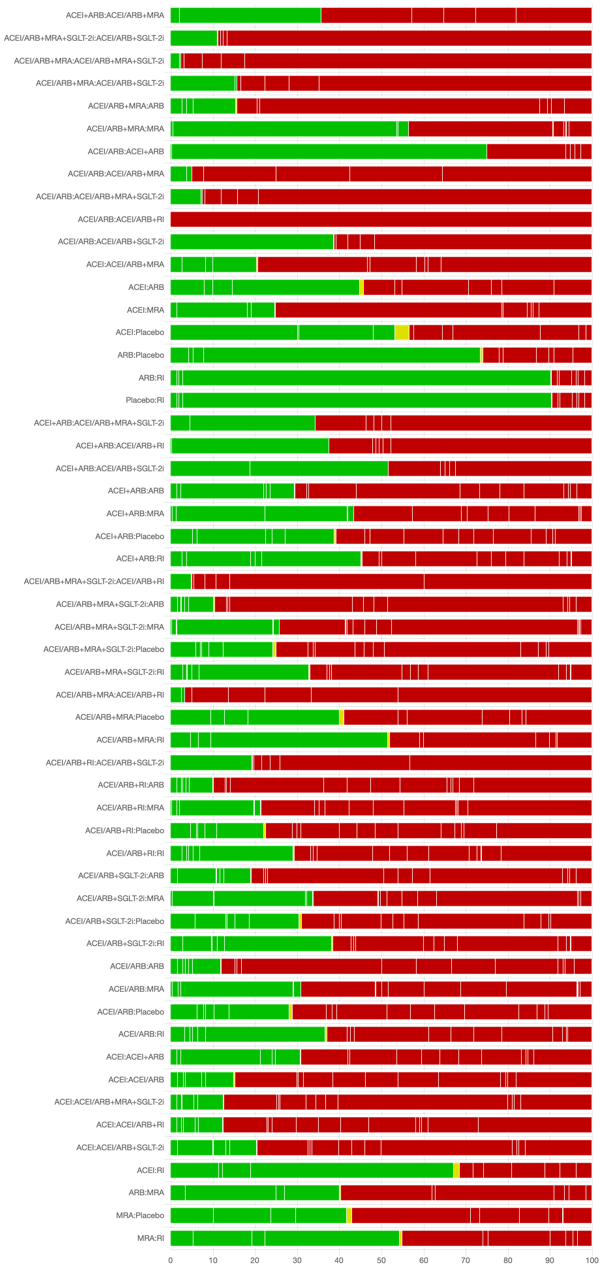
**

ACEI: angiotensin-converting enzyme inhibitors, ARB: angiotensin receptor blockers, MRA: mineralocorticoid receptor antagonists, RI: renin inhibitors.

**2.2 Supplementary Table S13: Evaluation of the Certainty of evidence Using CINEMA Framework.**

| Comparison | Number of studies | Within-study bias | Reporting bias | Indirectness | Imprecision | Heterogeneity | Incoherence | Confidence rating |
| --- | --- | --- | --- | --- | --- | --- | --- | --- |
| ACEI:ACEI/ARB+MRA | 2 | Major concerns | Low risk | No concerns | No concerns | Some concerns | No concerns | Very low |
| ACEI:ARB | 1 | Some concerns | Low risk | No concerns | No concerns | Major concerns | No concerns | Very low |
| ACEI:MRA | 1 | Major concerns | Low risk | No concerns | Some concerns | Some concerns | No concerns | Very low |
| ACEI:Placebo | 6 | Some concerns | Some concerns | No concerns | No concerns | Some concerns | No concerns | Moderate |
| ACEI/ARB:ACEI+ARB | 2 | No concerns | Low risk | No concerns | No concerns | Some concerns | No concerns | Moderate |
| ACEI+ARB:ACEI/ARB+MRA | 1 | Some concerns | Low risk | No concerns | No concerns | Major concerns | No concerns | Very low |
| ACEI/ARB:ACEI/ARB+MRA | 5 | Major concerns | Low risk | No concerns | No concerns | No concerns | No concerns | Very low |
| ACEI/ARB:ACEI/ARB+MRA+SGLT-2i | 1 | Major concerns | Low risk | No concerns | No concerns | Some concerns | No concerns | Very low |
| ACEI/ARB:ACEI/ARB+RI | 1 | Major concerns | Low risk | No concerns | Some concerns | Some concerns | No concerns | Very low |
| ACEI/ARB:ACEI/ARB+SGLT-2i | 2 | Some concerns | Low risk | No concerns | No concerns | Some concerns | No concerns | Moderate |
| ACEI/ARB+MRA:ACEI/ARB+MRA+SGLT-2i | 1 | Major concerns | Low risk | No concerns | No concerns | Major concerns | No concerns | Very low |
| ACEI/ARB+MRA:ACEI/ARB+SGLT-2i | 1 | Major concerns | Low risk | No concerns | No concerns | Some concerns | No concerns | Very low |
| ACEI/ARB+MRA:ARB | 1 | Major concerns | Low risk | No concerns | No concerns | Some concerns | No concerns | Very low |
| ACEI/ARB+MRA:MRA | 2 | Some concerns | Low risk | No concerns | Some concerns | No concerns | No concerns | Moderate |
| ACEI/ARB+MRA+SGLT-2i:ACEI/ARB+SGLT-2i | 1 | Major concerns | Low risk | No concerns | No concerns | Some concerns | No concerns | Very low |
| ARB:Placebo | 1 | Some concerns | Low risk | No concerns | Some concerns | No concerns | No concerns | Moderate |
| ARB:RI | 1 | No concerns | Low risk | No concerns | Some concerns | No concerns | No concerns | Moderate |
| Placebo:RI | 1 | No concerns | Low risk | No concerns | No concerns | Major concerns | No concerns | Very low |
| ACEI:ACEI+ARB | 0 | Some concerns | Low risk | No concerns | Some concerns | No concerns | No concerns | Moderate |
| ACEI:ACEI/ARB | 0 | Major concerns | Low risk | No concerns | No concerns | Major concerns | No concerns | Very low |
| ACEI:ACEI/ARB+MRA+SGLT-2i | 0 | Major concerns | Low risk | No concerns | Some concerns | No concerns | No concerns | Very low |
| ACEI:ACEI/ARB+RI | 0 | Major concerns | Low risk | No concerns | Major concerns | No concerns | No concerns | Very low |
| ACEI:ACEI/ARB+SGLT-2i | 0 | Major concerns | Low risk | No concerns | Some concerns | Some concerns | No concerns | Very low |
| ACEI:RI | 0 | Some concerns | Low risk | No concerns | Some concerns | No concerns | No concerns | Moderate |
| ACEI+ARB:ACEI/ARB+MRA+SGLT-2i | 0 | Some concerns | Low risk | No concerns | Some concerns | Some concerns | No concerns | Moderate |
| ACEI+ARB:ACEI/ARB+RI | 0 | Some concerns | Low risk | No concerns | Major concerns | No concerns | No concerns | Very low |
| ACEI+ARB:ACEI/ARB+SGLT-2i | 0 | Some concerns | Low risk | No concerns | Some concerns | No concerns | No concerns | Moderate |
| ACEI+ARB:ARB | 0 | Some concerns | Low risk | No concerns | Some concerns | No concerns | No concerns | Moderate |
| ACEI+ARB:MRA | 0 | Some concerns | Low risk | No concerns | Some concerns | No concerns | No concerns | Moderate |
| ACEI+ARB:Placebo | 0 | Some concerns | Low risk | No concerns | No concerns | Some concerns | No concerns | Moderate |
| ACEI+ARB:RI | 0 | Some concerns | Low risk | No concerns | No concerns | Some concerns | No concerns | Moderate |
| ACEI/ARB:ARB | 0 | Major concerns | Low risk | No concerns | No concerns | Major concerns | No concerns | Very low |
| ACEI/ARB:MRA | 0 | Some concerns | Low risk | No concerns | Some concerns | Some concerns | No concerns | Moderate |
| ACEI/ARB:Placebo | 0 | Some concerns | Low risk | No concerns | Some concerns | No concerns | No concerns | Moderate |
| ACEI/ARB:RI | 0 | Some concerns | Low risk | No concerns | Some concerns | No concerns | No concerns | Moderate |
| ACEI/ARB+MRA:ACEI/ARB+RI | 0 | Major concerns | Low risk | No concerns | Some concerns | Some concerns | No concerns | Very low |
| ACEI/ARB+MRA:Placebo | 0 | Some concerns | Low risk | No concerns | No concerns | No concerns | No concerns | Moderate |
| ACEI/ARB+MRA:RI | 0 | Some concerns | Low risk | No concerns | No concerns | No concerns | No concerns | Moderate |
| ACEI/ARB+MRA+SGLT-2i:ACEI/ARB+RI | 0 | Major concerns | Low risk | No concerns | Some concerns | Some concerns | No concerns | Very low |
| ACEI/ARB+MRA+SGLT-2i:ARB | 0 | Major concerns | Low risk | No concerns | No concerns | Some concerns | No concerns | Very low |
| ACEI/ARB+MRA+SGLT-2i:MRA | 0 | Some concerns | Low risk | No concerns | Some concerns | No concerns | No concerns | Moderate |
| ACEI/ARB+MRA+SGLT-2i:Placebo | 0 | Major concerns | Low risk | No concerns | No concerns | No concerns | No concerns | Very low |
| ACEI/ARB+MRA+SGLT-2i:RI | 0 | Some concerns | Low risk | No concerns | No concerns | Some concerns | No concerns | Moderate |
| ACEI/ARB+RI:ACEI/ARB+SGLT-2i | 0 | Major concerns | Low risk | No concerns | Major concerns | No concerns | No concerns | Very low |
| ACEI/ARB+RI:ARB | 0 | Major concerns | Low risk | No concerns | Major concerns | No concerns | No concerns | Very low |
| ACEI/ARB+RI:MRA | 0 | Major concerns | Low risk | No concerns | Major concerns | No concerns | No concerns | Very low |
| ACEI/ARB+RI:Placebo | 0 | Major concerns | Low risk | No concerns | Some concerns | No concerns | No concerns | Very low |
| ACEI/ARB+RI:RI | 0 | Some concerns | Low risk | No concerns | Some concerns | Some concerns | No concerns | Moderate |
| ACEI/ARB+SGLT-2i:ARB | 0 | Major concerns | Low risk | No concerns | Some concerns | Some concerns | No concerns | Very low |
| ACEI/ARB+SGLT-2i:MRA | 0 | Some concerns | Low risk | No concerns | Major concerns | No concerns | No concerns | Very low |
| ACEI/ARB+SGLT-2i:Placebo | 0 | Some concerns | Low risk | No concerns | Some concerns | No concerns | No concerns | Moderate |
| ACEI/ARB+SGLT-2i:RI | 0 | Some concerns | Low risk | No concerns | Some concerns | No concerns | No concerns | Moderate |
| ARB:MRA | 0 | Some concerns | Low risk | No concerns | Some concerns | Some concerns | No concerns | Moderate |
| MRA:Placebo | 0 | Some concerns | Low risk | No concerns | Some concerns | No concerns | No concerns | Moderate |
| MRA:RI | 0 | Some concerns | Low risk | No concerns | Some concerns | No concerns | No concerns | Moderate |

ACEI: angiotensin-converting enzyme inhibitors, ARB: angiotensin receptor blockers, MRA: mineralocorticoid receptor antagonists, RI: renin inhibitors.

**
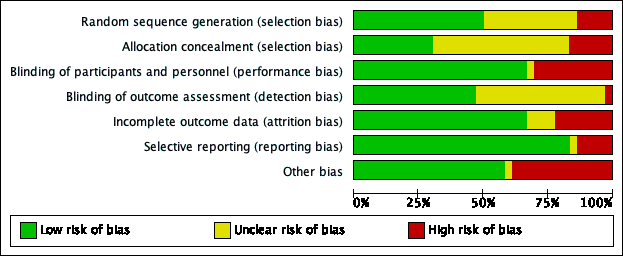
**

Supplementary Figure S1: Risk of bias graph: authors’ judgments about each risk of bias item presented as percentages across all included studies.

**
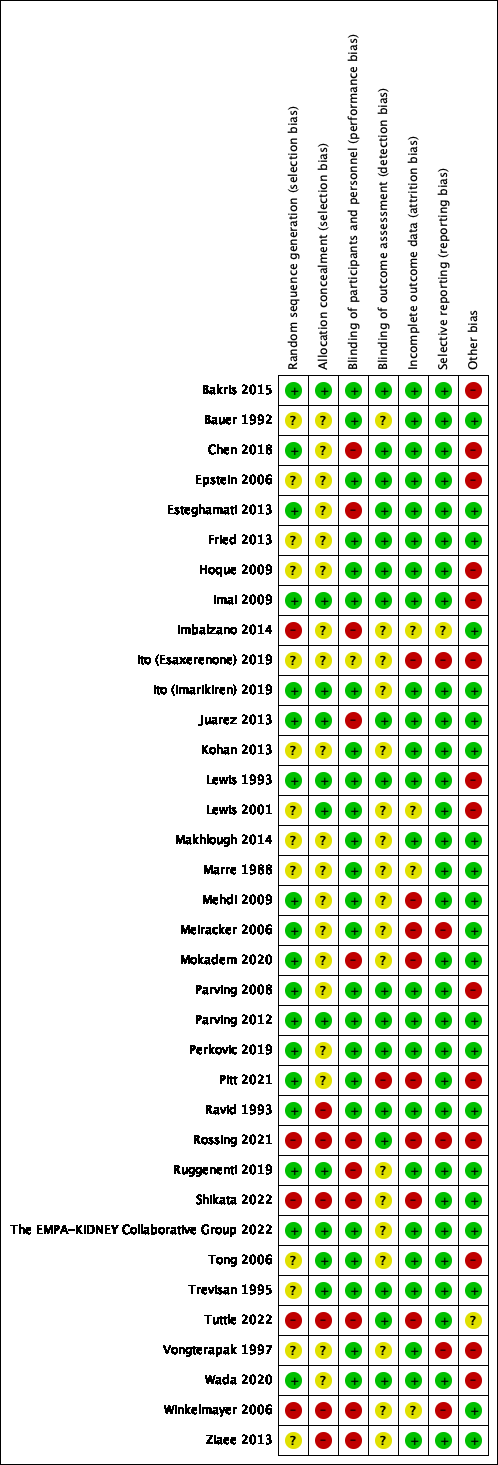
**

Supplementary Figure S2: Risk of bias summary: authors’ judgments about each risk of bias item for each included study.

**
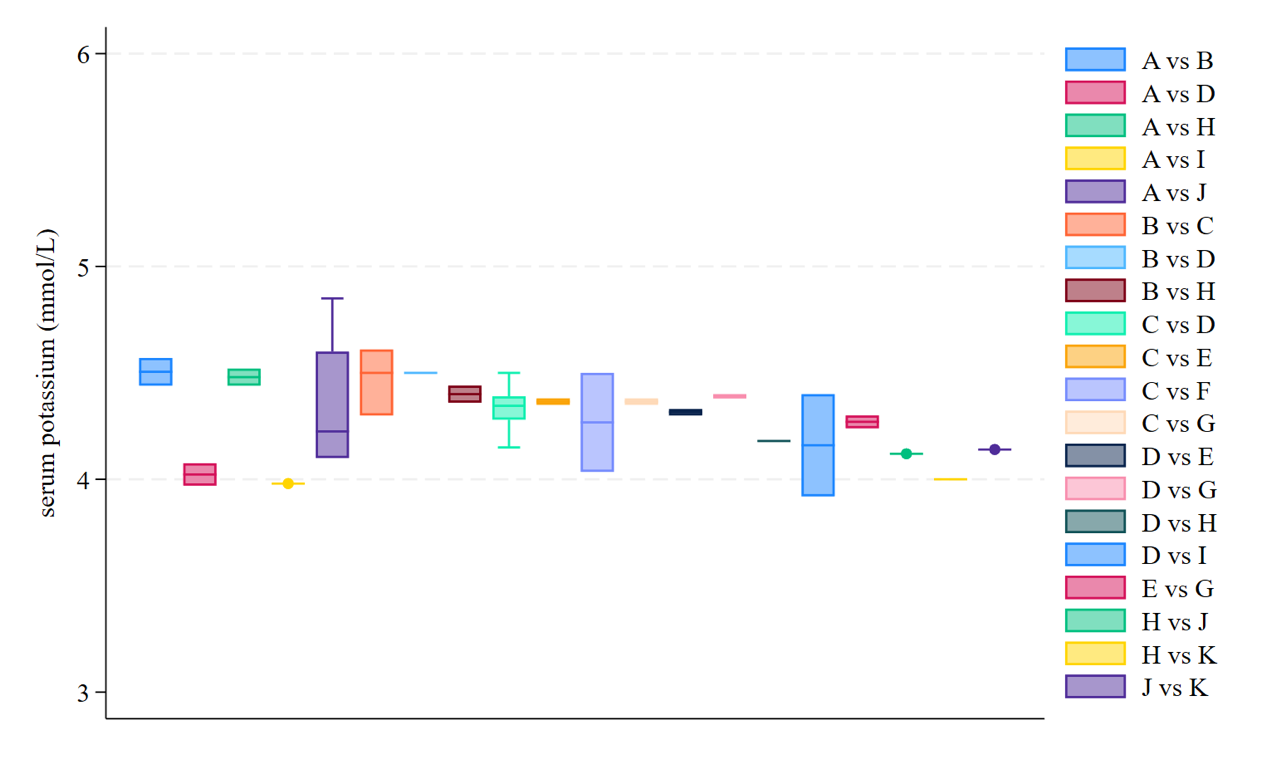
**

Supplementary Figure S3：Box plots showing the distribution of the baseline serum potassium (mmol/L) in the trials across the available direct comparisons. A=ACEI, B=ACEI + ARB, C=ACEI/ARB, D=ACEI/ARB + MRA, E=ACEI/ARB + MRA + SGLT-2i, F=ACEI/ARB + RI, G=ACEI/ARB + SGLT-2i, H=ARB, I=MRA, J=Placebo, K=RI, L=SGLT-2i

**
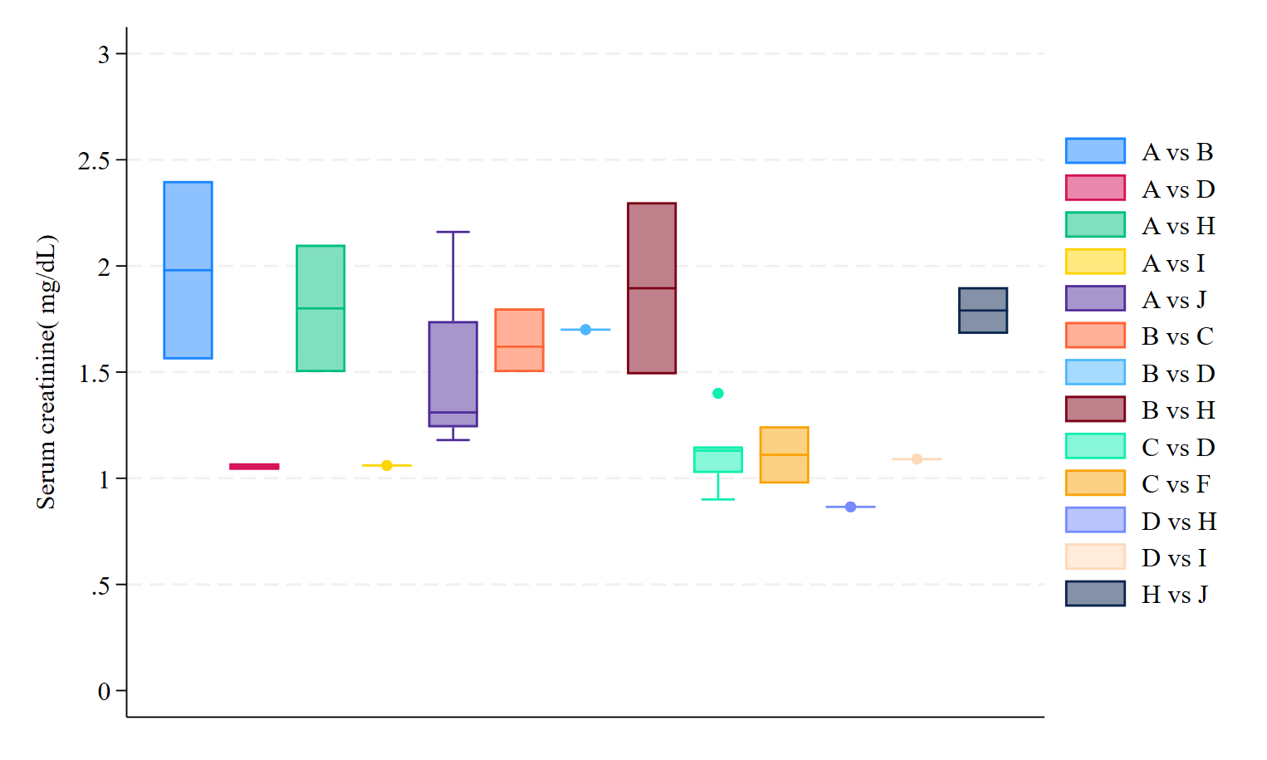
**

Supplementary Figure S4：Box plots showing the distribution of the baseline serum creatinine (mg/dL) in the trials across the available direct comparisons. A=ACEI, B=ACEI + ARB, C=ACEI/ARB, D=ACEI/ARB + MRA, E=ACEI/ARB + MRA + SGLT-2i, F=ACEI/ARB + RI, G=ACEI/ARB + SGLT-2i, H=ARB, I=MRA, J=Placebo, K=RI, L=SGLT-2i

**
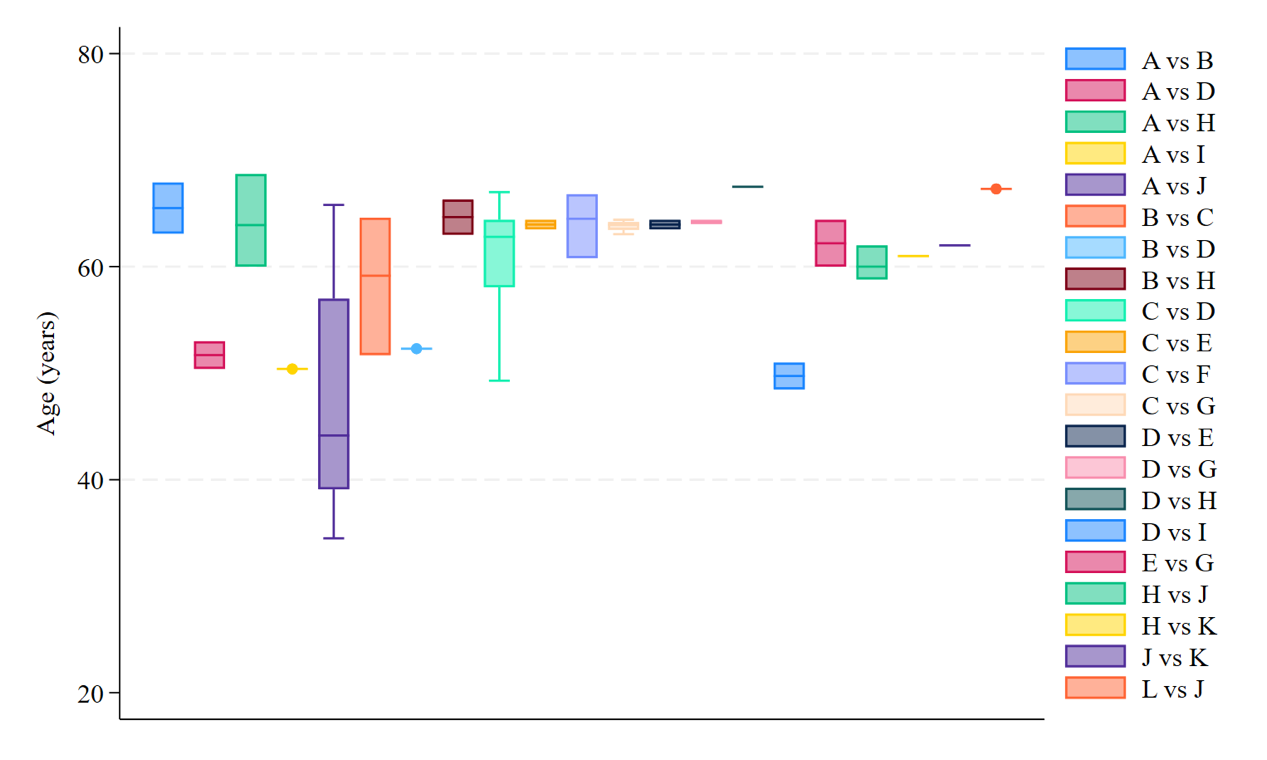
**

Supplementary Figure S5：Box plots showing the distribution of the age (years) in the trials across the available direct comparisons. A=ACEI, B=ACEI + ARB, C=ACEI/ARB, D=ACEI/ARB + MRA, E=ACEI/ARB + MRA + SGLT-2i, F=ACEI/ARB + RI, G=ACEI/ARB + SGLT-2i, H=ARB, I=MRA, J=Placebo, K=RI, L=SGLT-2i

**
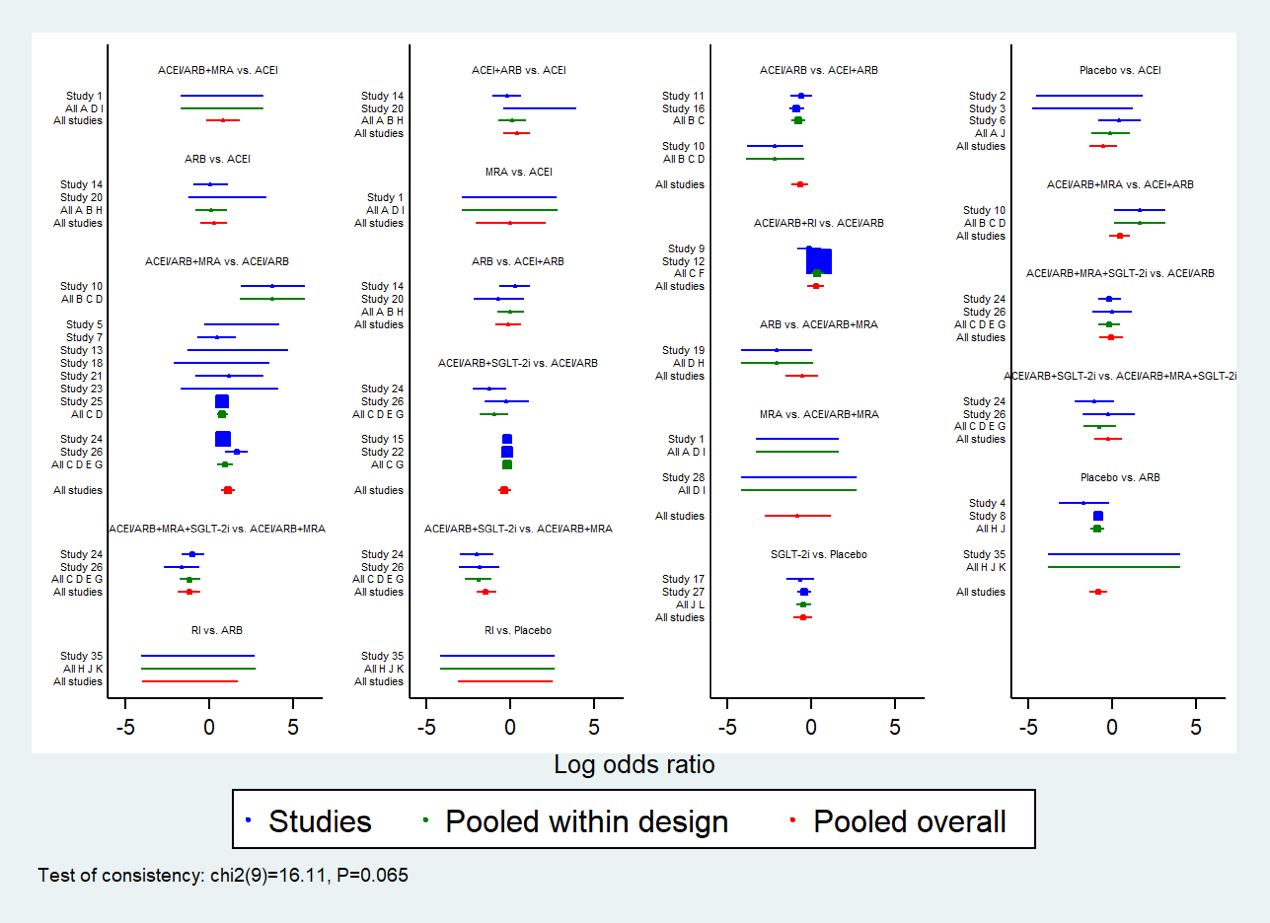
**

Supplementary Figure S6: Forest plot of eligible treatment comparisons for hyperkalemia. ACEI: angiotensin-converting enzyme inhibitors, ARB: angiotensin receptor blockers, MRA: mineralocorticoid receptor antagonists, RI: renin inhibitors.

**
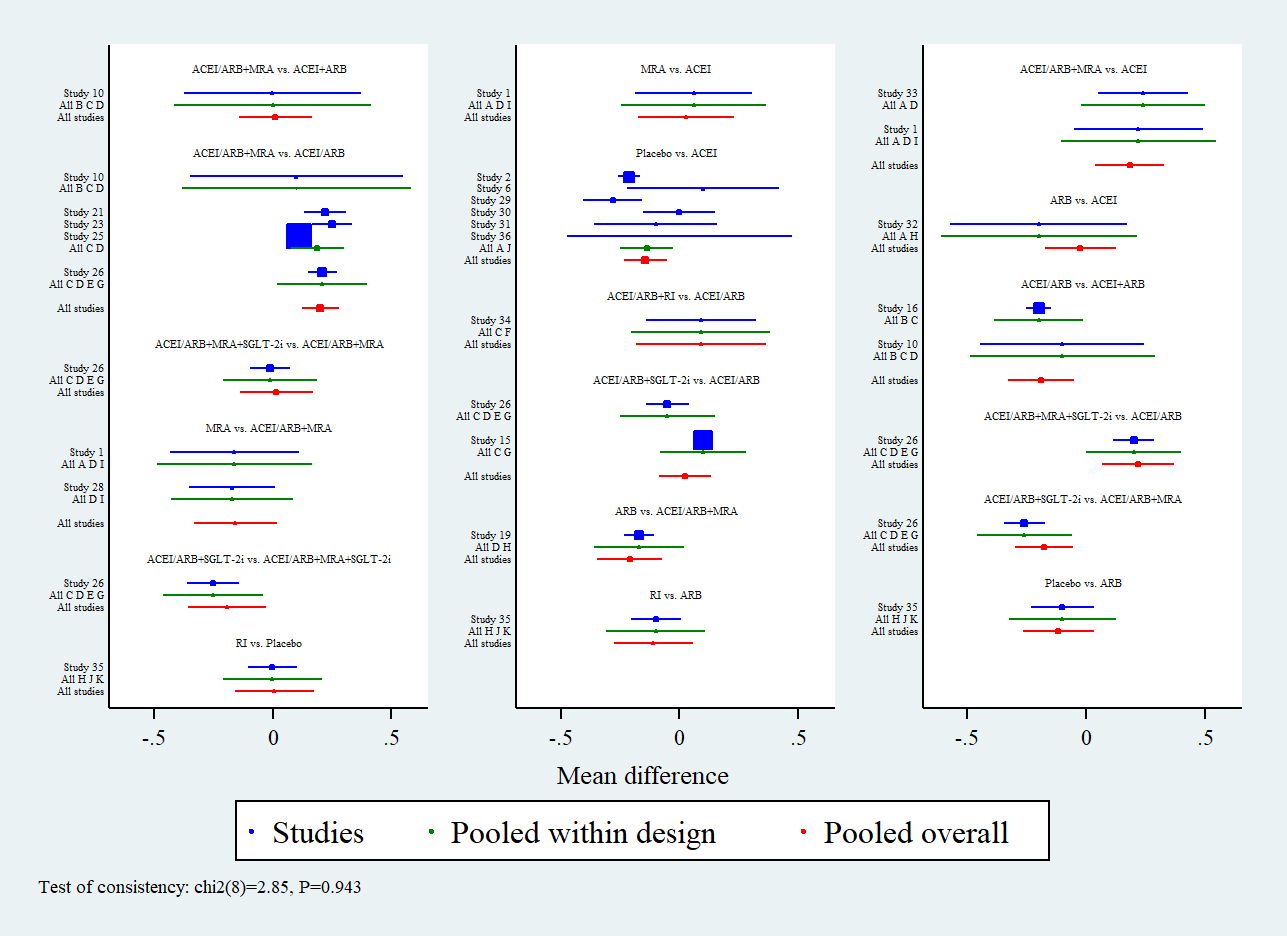
**

Supplementary Figure S7: Forest plot of eligible treatment comparisons for serum potassium. ACEI: angiotensin-converting enzyme inhibitors, ARB: angiotensin receptor blockers, MRA: mineralocorticoid receptor antagonists, RI: renin inhibitors.

**
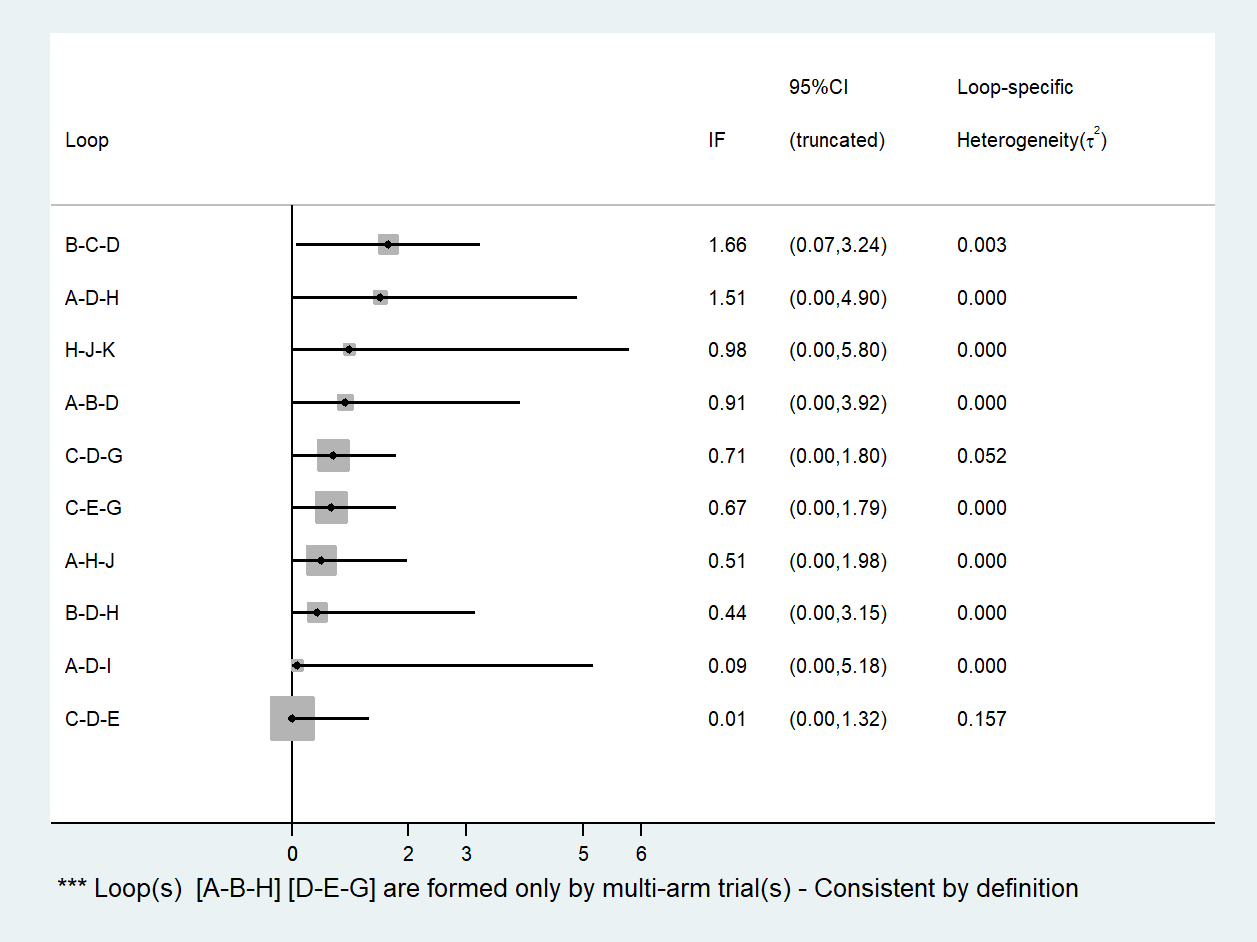
**

Supplementary Figure S8: Evaluation of inconsistency using loop-specific approach for hyperkalemia. A=ACEI, B=ACEI + ARB, C=ACEI/ARB, D=ACEI/ARB + MRA, E=ACEI/ARB + MRA + SGLT-2i, G=ACEI/ARB + SGLT-2i, H=ARB, I=MRA, J=Placebo, K=RI.

**
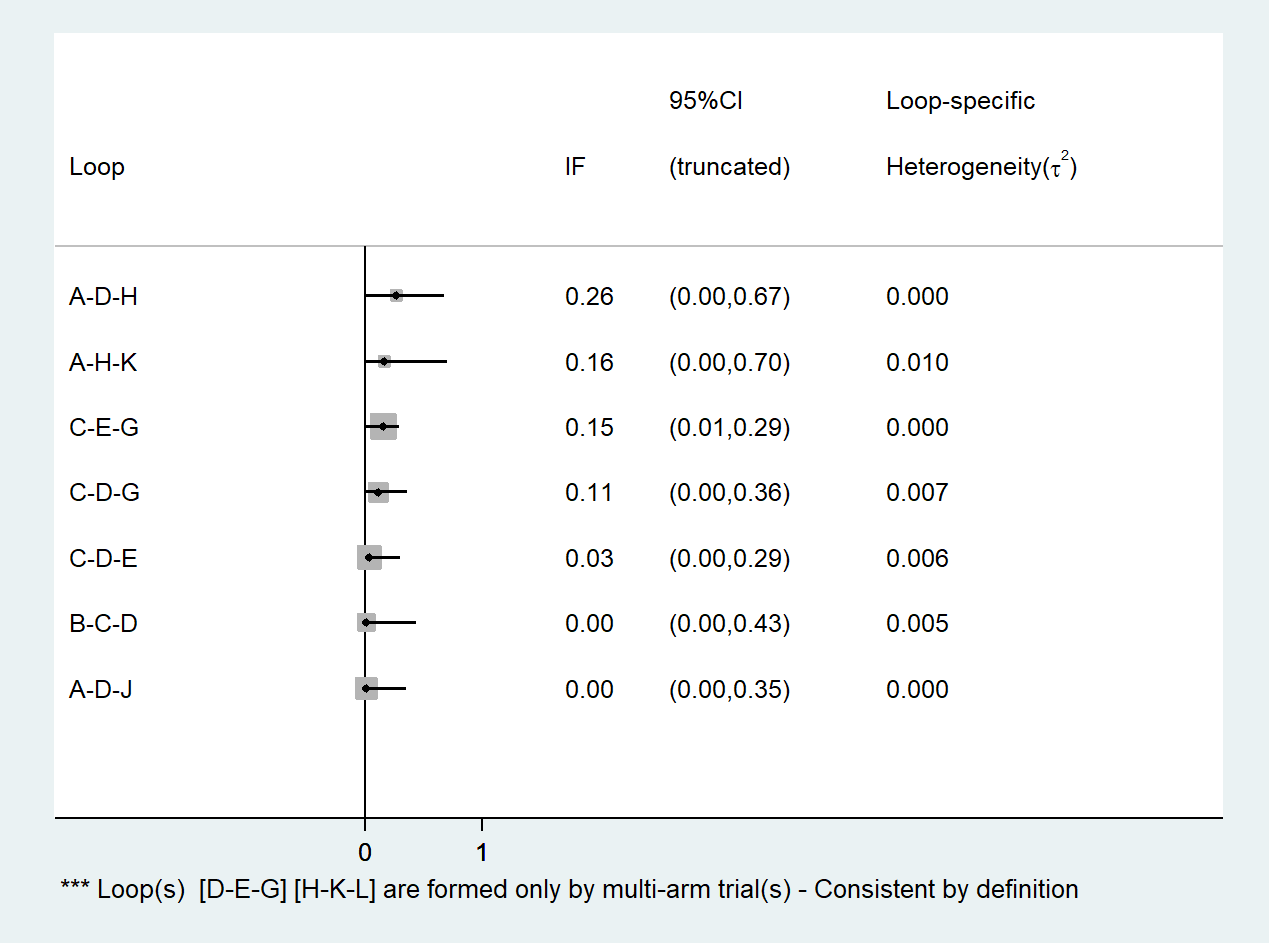
**

Supplementary Figure S9: Evaluation of inconsistency using loop-specific approach for serum potassium. A=ACEI, B=ACEI + ARB, C=ACEI/ARB, D=ACEI/ARB + MRA, E=ACEI/ARB + MRA + SGLT-2i, G=ACEI/ARB + SGLT-2i, H=ARB, J=Placebo, K=RI.

**
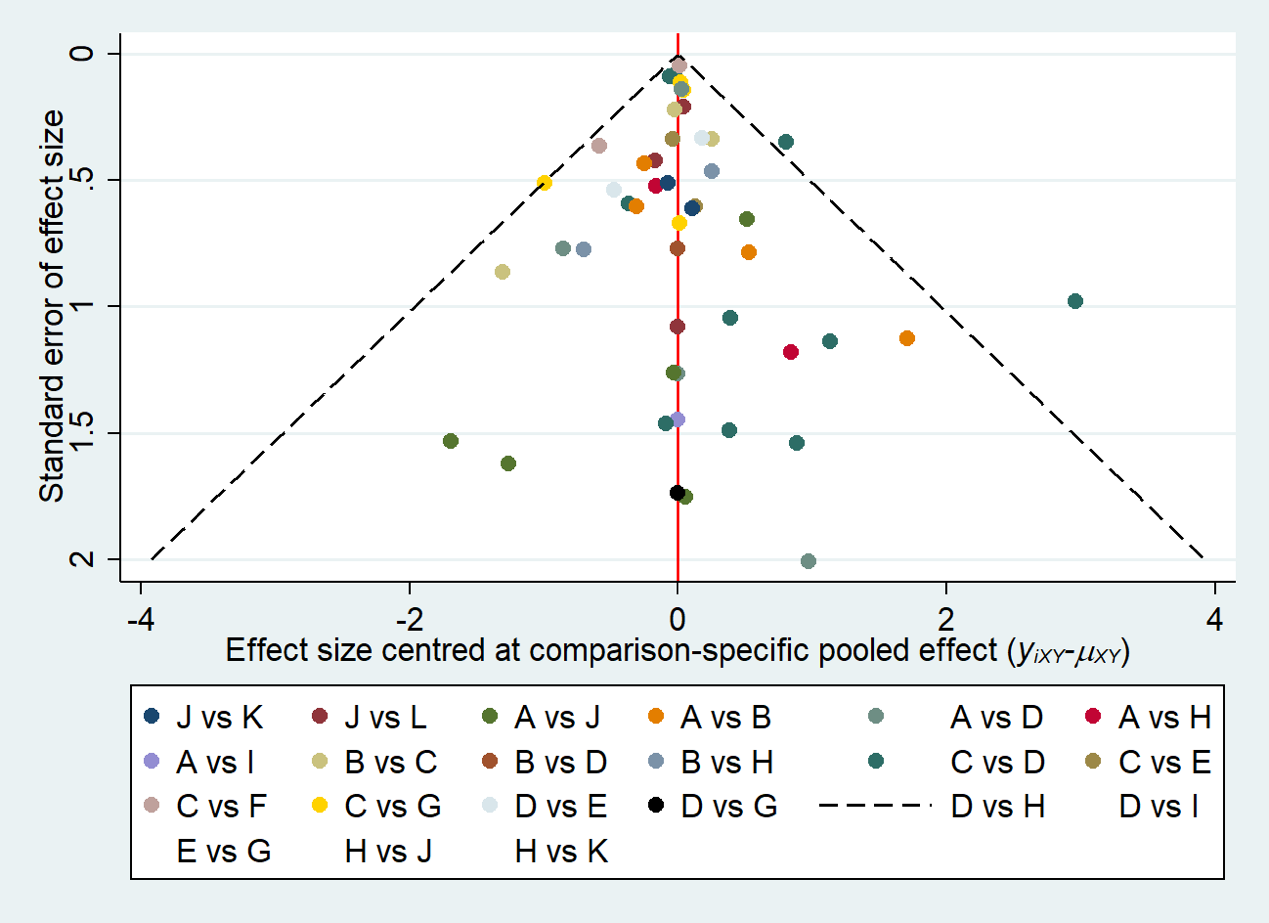
**

Supplementary Figure S10: Comparison-adjusted funnel plot for hyperkalemia. A=ACEI, B=ACEI + ARB, C=ACEI/ARB, D=ACEI/ARB + MRA, E=ACEI/ARB + MRA + SGLT-2i, F=ACEI/ARB + RI, G=ACEI/ARB + SGLT-2i, H=ARB, I=MRA, J=Placebo, K=RI, L=SGLT-2i.

**
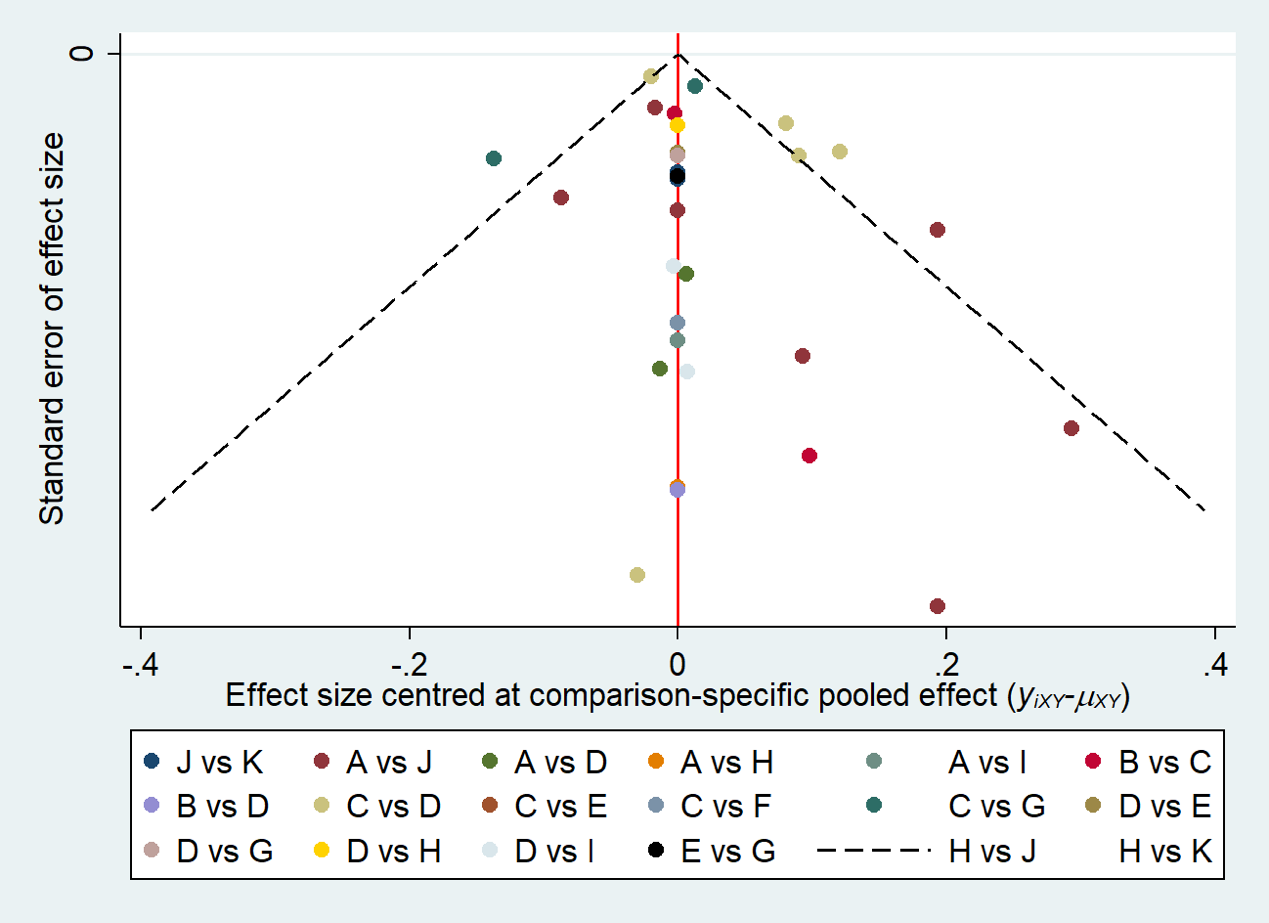
**

Supplementary Figure S11: Comparison-adjusted funnel plot for serum potassium. A=ACEI, B=ACEI + ARB, C=ACEI/ARB, D=ACEI/ARB + MRA, E=ACEI/ARB + MRA + SGLT-2i, F=ACEI/ARB + RI, G=ACEI/ARB + SGLT-2i, H=ARB, I=MRA, J=Placebo, K=RI.

**Reference**

[1] El Mokadem M, Abd El Hady Y, Aziz A.A Prospective Single-Blind Randomized Trial of Ramipril, Eplerenone and Their Combination in Type 2 Diabetic Nephropathy[J].Cardiorenal Med,2020, 10 (6): 392-401.

[2] Bauer J H, Reams G P, Hewett J, et al.A randomized, double-blind, placebo-controlled trial to evaluate the effect of enalapril in patients with clinical diabetic nephropathy[J].Am J Kidney Dis,1992, 20 (5): 443-57.

[3] Lewis E J, Hunsicker L G, Bain R P, et al.The effect of angiotensin-converting-enzyme inhibition on diabetic nephropathy[J].New England Journal of Medicine,1993, 329 (20): 1456-1462.

[4] Lewis E J, Hunsicker L G, Clarke W R, et al.Renoprotective effect of the angiotensin-receptor antagonist irbesartan in patients with nephropathy due to type 2 diabetes[J].N Engl J Med,2001, 345 (12): 851-60.

[5] Van Den Meiracker A H, Baggen R G A, Pauli S, et al.Spironolactone in type 2 diabetic nephropathy: Effects on proteinuria, blood pressure and renal function[J].Journal of Hypertension,2006, 24 (11): 2285-2292.

[6] Tong P C, Ko G T, Chan W B, et al.The efficacy and tolerability of fosinopril in Chinese type 2 diabetic patients with moderate renal insufficiency[J].Diabetes Obes Metab,2006, 8 (3): 342-7.

[7] Epstein M, Williams G H, Weinberger M, et al.Selective aldosterone blockade with eplerenone reduces albuminuria in patients with type 2 diabetes[J].Clin J Am Soc Nephrol,2006, 1 (5): 940-51.

[8] Winkelmayer W C, Zhang Z, Shahinfar S, et al.Efficacy and safety of angiotensin II receptor blockade in elderly patients with diabetes[J].Diabetes Care,2006, 29 (10): 2210-7.

[9] Parving H-H, Persson F, Lewis J B, et al.Aliskiren combined with losartan in type 2 diabetes and nephropathy[J].NEW ENGLAND JOURNAL OF MEDICINE,2008, 358 (23): 2433-2446.

[10] Mehdi U F, Adams-Huet B, Raskin P, et al.Addition of angiotensin receptor blockade or mineralocorticoid antagonism to maximal angiotensin-converting enzyme inhibition in diabetic nephropathy[J].J Am Soc Nephrol,2009, 20 (12): 2641-50.

[11] Imai E, Chan J C N, Ito S, et al.Effects of olmesartan on renal and cardiovascular outcomes in type 2 diabetes with overt nephropathy: a multicentre, randomised, placebo-controlled study[J].DIABETOLOGIA,2011, 54 (12): 2978-2986.

[12] Parving H H, Brenner B M, Mcmurray J J, et al.Cardiorenal end points in a trial of aliskiren for type 2 diabetes[J].N Engl J Med,2012, 367 (23): 2204-13.

[13] Esteghamati A, Noshad S, Jarrah S, et al.Long-term effects of addition of mineralocorticoid receptor antagonist to angiotensin II receptor blocker in patients with diabetic nephropathy: a randomized clinical trial[J].Nephrol Dial Transplant,2013, 28 (11): 2823-33.

[14] Fernandez Juarez G, Luño J Fau - Barrio V, Barrio V Fau - De Vinuesa S G, et al.Effect of dual blockade of the renin-angiotensin system on the progression of type 2 diabetic nephropathy: a randomized trial[J] (1523-6838 (Electronic)).

[15] Herrington W G, Staplin N, Wanner C, et al.Empagliflozin in Patients with Chronic Kidney Disease[J].N Engl J Med,2023, 388 (2): 117-127.

[16] Fried L F, Emanuele N Fau - Zhang J H, Zhang Jh Fau - Brophy M, et al.Combined angiotensin inhibition for the treatment of diabetic nephropathy[J] (1533-4406 (Electronic)).

[17] Kohan D E, Fioretto P, Tang W, et al.Long-term study of patients with type 2 diabetes and moderate renal impairment shows that dapagliflozin reduces weight and blood pressure but does not improve glycemic control[J].Kidney Int,2014, 85 (4): 962-71.

[18] Bakris G L, Agarwal R, Chan J C, et al.Effect of Finerenone on Albuminuria in Patients With Diabetic Nephropathy A Randomized Clinical Trial[J].JAMA-JOURNAL OF THE AMERICAN MEDICAL ASSOCIATION,2015, 314 (9): 884-894.

[19] Chen Y, Liu P, Chen X, et al.Effects of Different Doses of Irbesartan Combined With Spironolactone on Urinary Albumin Excretion Rate in Elderly Patients With Early Type 2 Diabetic Nephropathy[J].Am J Med Sci,2018, 355 (5): 418-424.

[20] Ruggenenti P, Trillini M, P. Barlovic D, et al.Effects of valsartan, benazepril and their combination in overt nephropathy of type 2 diabetes: A prospective, randomized, controlled trial[J].Diabetes, Obesity and Metabolism,2019, 21 (5): 1177-1190.

[21] Ito S, Shikata K, Nangaku M, et al.Efficacy and Safety of Esaxerenone (CS-3150) for the Treatment of Type 2 Diabetes with Microalbuminuria: A Randomized, Double-Blind, Placebo-Controlled, Phase II Trial[J].Clin J Am Soc Nephrol,2019, 14 (8): 1161-1172.

[22] Perkovic V, Jardine M J, Neal B, et al.Canagliflozin and Renal Outcomes in Type 2 Diabetes and Nephropathy[J].N Engl J Med,2019, 380 (24): 2295-2306.

[23] Wada T, Inagaki M, Yoshinari T, et al.Apararenone in patients with diabetic nephropathy: results of a randomized, double-blind, placebo-controlled phase 2 dose-response study and open-label extension study[J].Clin Exp Nephrol,2021, 25 (2): 120-130.

[24] Rossing P, Filippatos G, Agarwal R, et al.Finerenone in Predominantly Advanced CKD and Type 2 Diabetes With or Without Sodium-Glucose Cotransporter-2 Inhibitor Therapy[J].Kidney international reports,2022, 7 (1): 36‐45.

[25] Pitt B, Filippatos G, Agarwal R, et al.Cardiovascular Events with Finerenone in Kidney Disease and Type 2 Diabetes[J].NEW ENGLAND JOURNAL OF MEDICINE,2021, 385 (24): 2252-2263.

[26] Shikata K, Ito S, Kashihara N, et al.Reduction in the magnitude of serum potassium elevation in combination therapy with esaxerenone (CS-3150) and sodium-glucose cotransporter 2 inhibitor in patients with diabetic kidney disease: Subanalysis of two phase III studies[J].J Diabetes Investig,2022, 13 (7): 1190-1202.

[27] Tuttle K R, Levin A, Nangaku M, et al.Safety of Empagliflozin in Patients With Type 2 Diabetes and Chronic Kidney Disease: Pooled Analysis of Placebo-Controlled Clinical Trials[J].Diabetes Care,2022, 45 (6): 1445-1452.

[28] Makhlough A, Kashi Z, Akha O, et al.Effect of spironolactone on diabetic nephropathy compared to the combination of spironolactone and losartan[J].Nephrourol Mon,2014, 6 (1): e12148.

[29] Ravid M, Savin H, Jutrin I, et al.Long-term stabilizing effect of angiotensin-converting enzyme inhibition on plasma creatinine and on proteinuria in normotensive type II diabetic patients[J].Ann Intern Med,1993, 118 (8): 577-81.

[30] Trevisan R, Tiengo A.Effect of low-dose ramipril on microalbuminuria in normotensive or mild hypertensive non-insulin-dependent diabetic patients. North-East Italy Microalbuminuria Study Group[J] (0895-7061 (Print)).

[31] Vongterapak S, Dahlan W, Nakasatien S, et al.Impediment of the progressions of microalbuminuria and hyperlipidemia in normotensive type 2 diabetes by low-dose ramipril[J].Chotmaihet thangphaet [Journal of the Medical Association of Thailand],1998, 81 (9): 671‐681.

[32] Hoque R, Rahman M S, Iqbal M.Effect of enalapril and losartan on proteinuria in type 2 diabetic nephropathy patients[J].Bangladesh Med Res Counc Bull,2009, 35 (2): 44-8.

[33] Ziaee A, Vaezi A A, Oveisi S, et al.Effects of additive therapy with spironolactone on albuminuria in diabetes mellitus: a pilot randomized clinical trial[J].Caspian journal of internal medicine,2013, 4 (2): 648‐653.

[34] Imbalzano E, Scarpelli M, Mandraffino G, et al.Combination therapy with aliskiren versus ramipril or losartan added to conventional therapy in patients with type 2 diabetes mellitus, uncontrolled hypertension and microalbuminuria[J].Journal of the renin-angiotensin-aldosterone system : JRAAS,2015, 16 (4): 956‐964.

[35] Ito S, Kagawa T, Saiki T, et al.Efficacy and Safety of Imarikiren in Patients with Type 2 Diabetes and Microalbuminuria: A Randomized, Controlled Trial[J].Clin J Am Soc Nephrol,2019, 14 (3): 354-363.

[36] Marre M, Chatellier G Fau - Leblanc H, Leblanc H Fau - Guyene T T, et al.Prevention of diabetic nephropathy with enalapril in normotensive diabetics with microalbuminuria[J] (0959-8138 (Print)).
